# Tracking Active Site Formation during Oxidative Activation of Copper‐Exchanged Zeolites for Methane‐to‐Methanol Conversion

**DOI:** 10.1002/advs.202413870

**Published:** 2025-02-14

**Authors:** Andreas Brenig, Jörg W. A. Fischer, Daniel Klose, Gunnar Jeschke, Jeroen A. van Bokhoven, Vitaly L. Sushkevich

**Affiliations:** ^1^ Institute for Chemical and Bioengineering ETH Zurich Vladimir‐Prelog‐Weg 1–5/10 Zurich 8093 Switzerland; ^2^ Paul Scherrer Institute Center for Energy and Environmental Sciences Forschungsstrasse 111 Villigen 5232 Switzerland; ^3^ Institute for Molecular Physical Science ETH Zurich Vladimir‐Prelog‐Weg 1–5/10 Zurich 8093 Switzerland

**Keywords:** copper, heterogeneous catalysis, methane‐to‐methanol, operando spectroscopy, zeolites

## Abstract

The evolution of active sites in Cu‐zeolites for the CH_4_‐to‐CH_3_OH conversion has been investigated during oxidative treatment in O_2_. Three samples with different frameworks but comparable Cu loadings and Si/Al ratios have been prepared to assess the influence of topology on material oxidizability and the nature of the generated Cu(II) species. Complementary spectroscopic studies highlight that isomeric Cu(II) centers hosted within different topologies are characterized by distinct formation rates. In turn, the framework‐specific kinetics of Cu(II) site generation regulate the overall oxidation potential of the individual zeolites. Apart from the topology, the formation rate of different Cu(II) species is governed by their specific structure, with dimeric Cu(II) centers ([Cu_2_(µ‐O)]^2+^) being generated faster than monomeric ([CuOH]^+^, Cu^2+^) ones. Elevated temperatures accelerate the evolution of Cu(II) monomers but cause [Cu_2_(µ‐O)]^2+^ to undergo autoreduction. The reversibility of this process is framework‐dependent. Consequently, even though two types of [Cu_2_(µ‐O)]^2+^ form at low temperatures in each material, only specific ones remain after high‐temperature treatment. The autoreduction of [Cu_2_(µ‐O)]^2+^ is accompanied by its transient reduction by hydrocarbon residues, originating from the preceding treatment in CH_4_. The oxidative decomposition of these impurities yields H_2_O, which adsorbs on [Cu_2_(µ‐O)]^2+^ masks their spectroscopic fingerprints, and renders them inactive.

## Graphical abstract

The oxidative generation of Cu(II) active sites in Cu‐exchanged zeolites for the CH_4_‐to‐CH_3_OH conversion has been studied. Complementary in situ/operando spectroscopic techniques highlight that the formation rate and the stability of different Cu(II) species are governed by their structure and the zeolite topology. At elevated temperatures, the Cu centers are involved in a series of parallel oxidation and (auto‐)reduction processes.

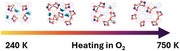

## 1 Introduction

Cu‐zeolites can be employed in a variety of redox reactions such as the selective catalytic reduction (SCR) of NO_x_ with NH_3_ or the oxidative dehydrogenation of ethane and propane.^[^ ^1^, ^2^ ^]^ Moreover, they are capable of selectively oxidizing CH_4_ into CH_3_OH at mild temperatures and hence could be employed in scale‐flexible CH_4_ abatement units for off‐grid oil extraction sites and landfills.^[^ ^3^, ^4^, ^5^ ^]^ This reaction is frequently performed in a stepwise and stoichiometric chemical looping procedure, involving a Cu(II)/Cu(I) redox cycle.^[^ ^3^, ^6^, ^7^, ^8^ ^]^ An initial treatment of the material in an oxidizing environment results in the formation of Cu(II) active sites, which are reduced to Cu(I) throughout the subsequent reaction with CH_4_.^[^ ^7^, ^8^ ^]^ Owing to the temporal separation of the redox half‐reactions and the chemisorption of intermediates on the zeolite, the generated CH_3_OH is physically and chemically protected, thus mitigating its thermodynamically favorable overoxidation.^[^ ^9^, ^10^, ^11^ ^]^

The Cu‐zeolite's CH_3_OH productivity and selectivity are governed by the distinct redox and kinetic properties of the incorporated Cu(II) species.^[^ ^12^, ^13^, ^14^, ^15^, ^16^ ^]^ Therefore, special emphasis has been placed on identifying their structural features and determining the influence of material composition (i.e., Cu loading, Si/Al ratio) and zeolite topology on their formation. So far, a multitude of active Cu(II) centers with varying nuclearity and number of extra‐framework O‐ligands (O_ef_) has been proposed, including monomeric,^[^ ^15^, ^17^, ^18^ ^]^ dimeric,^[^ ^19^, ^20^ ^]^ trimeric,^[^ ^21^, ^22^ ^]^ and oligomeric^[^ ^23^, ^24^ ^]^ Cu(II) motifs. However, the majority of studies have focused on the behavior of these Cu(II) sites during their interaction with CH_4_, thereby disregarding the processes occurring during the preceding oxidative treatment. Considering that material activation has a profound impact on CH_3_OH productivity as well as the type and fraction of formed Cu(II) centers, an in‐depth understanding of this step is essential to obtain a holistic picture of the overall redox cycle.^[^ ^6^, ^25^, ^26^, ^27^ ^]^ This is further emphasized by the fact that a more controllable Cu(II) active site generation represents a key opportunity for the rational design of materials with precisely tailored properties.

Oxidative activation can be performed by subjecting the material to synthetic air,^[^ ^5^ ^]^ O_2_,^[^ ^20^ ^]^ H_2_O,^[^ ^28^ ^]^ NO,^[^ ^29^ ^]^ N_2_O,^[^ ^20^ ^]^ and H_2_O_2_,^[^ ^30^ ^]^ whereby the first two options are the only economically viable ones.^[^ ^4^, ^5^, ^31^ ^]^ In the case of activation by O_2_, the CH_3_OH productivity of most Cu‐zeolites increases monotonically upon raising the activation temperature to ≈773 K and then quickly levels off.^[^ ^25^, ^32^, ^33^, ^34^, ^35^, ^36^, ^37^ ^]^ Noteworthy, a typical optimal temperature at 623–723 K has been identified for materials exhibiting a Cu content of ≥ 6 wt%, which has been attributed to the aggregation of Cu(II) into inert clusters at higher temperatures.^[^ ^32^ ^]^ The influence of activation time on sample performance is less established. Most studies report a logarithmic relationship between CH_3_OH productivity and activation duration at temperatures between 473 and 723 K.^[^ ^25^, ^38^, ^39^ ^]^ In contrast, Ikuno et al. noticed a constant CH_3_OH output in the regime from 623 to 773 K when prolonging the activation period.^[^ ^34^ ^]^ Therefore, it is not clear whether material activation is thermodynamically or kinetically controlled. Similarly, opposing observations regarding the effect of O_2_ partial pressure on CH_3_OH productivity have been made at otherwise comparable activation conditions.^[^ ^25^, ^35^, ^38^, ^40^ ^]^

Relating these process parameters not only to the Cu‐zeolite's performance but also to the chemical processes occurring during oxidative treatment is particularly challenging. This is also highlighted by the fact that Cu(II) active site formation likely proceeds in a different manner depending on the initial state of the material. In the case of as‐prepared hydrated materials, Cu is already present as Cu(II) in the form of mobile [Cu(H_2_O)_6_]^2+^ and [Cu(H_2_O)_5_OH]^+^ complexes.^[^ ^41^, ^42^ ^]^ Dehydration induces migration of Cu(II) toward specific ion exchange positions and yields bare Cu^2+^, [CuOH]^+^ and [Cu_2_(µ‐O)]^2+^.^[^ ^41^, ^42^, ^43^, ^44^, ^45^, ^46^, ^47^ ^]^ The latter is believed to form via the self‐organization of two proximal [CuOH]^+^ species into [Cu_2_(µ‐OH)_2_]^2+^, which transforms into [Cu_2_(µ‐O)]^2+^ by dehydration.^[^ ^39^, ^43^, ^44^, ^45^, ^46^, ^48^ ^]^ Under these conditions, the generation of the former three Cu(II) centers, which all have been demonstrated to engage in partial CH_4_ oxidation, does not strictly necessitate the involvement of an oxidant.^[^ ^14^, ^15^, ^17^, ^18^, ^20^ ^]^ However, the presence of O_2_ minimizes the temperature‐induced autoreduction of [Cu_2_(µ‐O)]^2+^, which otherwise leads to the spontaneous release of O_2_ accompanied with the formation of Cu(I).^[^ ^43^, ^44^, ^45^, ^46^, ^49^, ^50^, ^51^, ^52^ ^]^ Notably, the creation of other frequently proposed Cu(II) active site motifs (e.g., [Cu_3_(µ‐O)_3_]^2+^,^[^ ^34^, ^48^ ^]^ [Cu(η^1^‐O_2_•)]^+^,^[^ ^25^, ^39^, ^53^ ^]^ [Cu(η^2^‐O_2_•)]^+^,^[^ ^25^, ^39^, ^53^ ^]^ [Cu_2_(µ‐1,2‐O_2_)]^2+ [^ ^25^, ^47^, ^53^ ^]^) requires a certain degree of Cu(II) autoreduction. Here, the homolytic elimination of •OH from [CuOH]^+^ is commonly assumed to be the source of Cu(I).^[^ ^25^, ^34^, ^39^, ^47^, ^53^, ^54^, ^55^ ^]^ However, this reaction is partially based on a previously refuted autoreduction mechanism, which challenges the role of [CuOH]^+^ in the creation of these Cu(II) centers.^[^ ^56^, ^57^, ^58^, ^59^, ^60^ ^]^ This is additionally emphasized by the obscure fate of the •OH radical and the disparate reports concerning the autoreducibility of [CuOH]^+^ compared to that of other Cu‐oxo sites.^[^ ^15^, ^25^, ^55^, ^61^, ^62^ ^]^

Conversely, activation of dry and reduced Cu‐zeolites, present after material reaction with CH_4_, involves an actual oxidation of Cu(I) by O_2_. Depending on the specific Cu‐Cu distance, adsorption of O_2_ on adjacent Cu(I) pairs can result in the formation of [Cu_2_(µ‐1,2‐O_2_)]^2+^ or [Cu_2_(µ‐η^2^:η^2^‐O_2_)]^2+^.^[^ ^25^, ^47^, ^63^, ^64^, ^65^ ^]^ Noteworthy, these reactions are strongly exothermic (ΔE_f_ ranges from −142 to −268 kJ mol^−1^), suggesting that the generation of those Cu(II) species is disfavored at the high activation temperatures required to optimize CH_3_OH productivity.^[^ ^35^, ^63^, ^65^ ^]^ This is especially remarkable considering that [Cu_2_(µ‐η^2^:η^2^‐O_2_)]^2+^ has been reported to act as a precursor for [Cu_2_(µ‐O)]^2+^ via O─O bond cleavage.^[^ ^63^, ^64^ ^]^ Whether the resulting second O moiety yields another [Cu_2_(µ‐O)]^2+^ center or is incorporated into the zeolite lattice in the form of a [Si_2_(µ‐1,2‐O_2_)]^6+^ site remains a matter of debate.^[^ ^63^ ^]^ Notably, this issue does not arise in the case of the transformation of [Cu_3_(µ_3_‐O)]^2+^ into [Cu_3_(µ‐O)_3_]^2+^ since the latter can accommodate two O^2−^ ions.^[^ ^63^ ^]^ Interaction of O_2_ with isolated Cu(I) sites results in the creation of both [Cu(η^1^‐O_2_•)]^+^ and [Cu(η^2^‐O_2_•)]^+^, but it is not obvious if and how these centers can convert into Cu^2+^ and [CuOH]^+^.^[^ ^66^, ^67^ ^]^

Irrespective of the initial state of the material, the nature of the created Cu(II) active sites is ultimately governed by the framework of the corresponding Cu‐zeolite and its elemental composition.^[^ ^6^, ^23^, ^68^ ^]^ Consequently, the Cu speciation is controlled by the spatial arrangement of the particular ion exchange positions, the distribution of Al over the tetrahedral sites (T‐sites), the Cu loading, and the nature of the co‐cations.^[^ ^13^, ^54^, ^65^, ^69^, ^70^ ^]^ The influence of the topology on the oxidative formation of distinct Cu‐oxo motifs, such as [Cu_2_(µ‐O)]^2+^, has been highlighted by Vilella et al.^[^ ^69^ ^]^ These authors demonstrated that the adsorption energy of an O atom on proximal Cu(I) centers situated in the eight‐membered ring (MR) of CHA is lower than the one in the ten‐MR of MFI at the optimal Al‐Al spacing necessary to stabilize [Cu_2_(µ‐O)]^2+^. This was attributed to the shorter Cu–Cu separation in the former case, which requires a higher deformation energy to fit the O atom in between the two Cu(I) ions. However, the direct consequences of the framework on the oxidizability of Cu‐zeolites with different topologies as well as the formation rates of distinct Cu(II) species within these materials remain elusive.

**Scheme 1:**
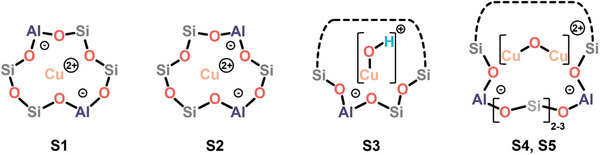
Representative illustrations of the identified monomeric (S1–S3) and dimeric (S4, S5) Cu(II) species in the examined Cu‐zeolites. Dashed lines correspond to the remaining part of an ion exchange position with variable dimensions. The Cu(II) dimers S4 and S5 differ in their Cu–Cu distance (S4: 2.9 Å, S5: 3.2 Å) and redox properties.^[^ ^12^, ^14^ ^]^

In this contribution, the oxidative re‐activation of Cu‐zeolites with O_2_ after reduction in CH_4_ is investigated by means of O_2_ temperature‐programmed oxidation (O_2_‐TPO) and isothermal re‐activation experiments. Variation of the sample topology (MOR, MFI, CHA) at similar Cu loading and Si/Al ratio provides insight into the framework‐dependence of material oxidizability and Cu(II) species formation. In situ/operando X‐ray absorption near edge structure (XANES), electron paramagnetic resonance (EPR), and ultraviolet–visible (UV–vis) spectroscopy demonstrate that specific monomeric and dimeric Cu(II) centers are generated in each Cu‐zeolite during treatment in O_2_. As summarized in **Scheme** **1**, this includes two types of bare Cu^2+^ ions characterized by specific arrangements of the surrounding Al T‐site pairs (S1, S2), [CuOH]^+^ species (S3) as well as two [Cu_2_(µ‐O)]^2+^ motifs, exhibiting different Cu‐Cu distances and featuring distinct redox properties (S4, S5).^[^ ^12^, ^16^ ^]^ The formation rate and high‐temperature stability of the individual Cu(II) sites are controlled by their distinct structure and the zeolite topology. Additionally, the characteristic electronic absorption bands of [Cu_2_(µ‐O)]^2+^ are affected by the reversible adsorption of H_2_O, arising from the oxidative decomposition of hydrocarbon residues formed after material reduction with CH_4_.

## 2 Results and Discussion

The oxidative formation of S1–S5 in three different Cu‐exchanged zeolites was monitored. Samples are labeled as Cu*_X_* ZEO*_Y_* where *_X_*, ZEO, and *_Y_* correspond to the Cu loading in wt%, the zeolite topology (MOR, MFI, CHA), and the Si/Al ratio. Results of the physicochemical characterization, including elemental analysis, N_2_ physisorption, and powder X‐ray diffraction (PXRD) are presented in Table S1 and Figure S1 (Supporting Information). Each sample was subjected to an in situ reductive treatment in CH_4_ at 753 K before any measurement.

**Figure 1:**
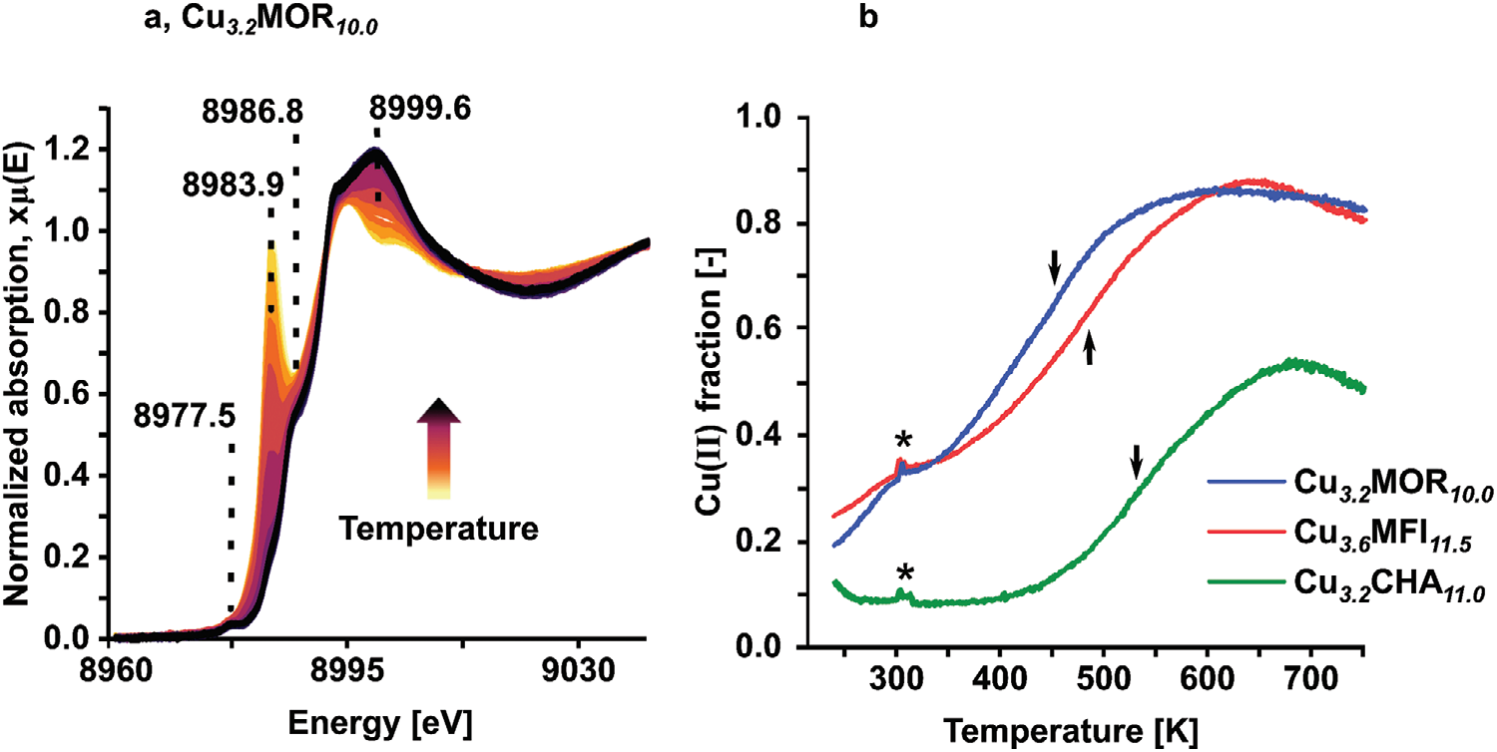
Exemplary in situ Cu K‐edge XANES spectra of Cu_3.2_MOR_10.0_ during O_2_‐TPO a). The fraction of Cu(II) in Cu_3.2_MOR_10.0_, Cu_3.6_MFI_11.5_, and Cu_3.2_CHA_11.0_ as a function of temperature as determined by LCF b). Arrows indicate the inflection points in the temperature range between 300 and 753 K. Glitches (indicated by *) originate from the manual switch between the cryostream and the heating jacket.

**Figure** **1**a shows an exemplary series of Cu K‐edge XANES spectra of Cu*_3.2_* MOR*_10.0_* recorded throughout O_2_‐TPO between 240 and 750 K (6 K min^−1^). The corresponding spectra of Cu*_3.6_* MFI*_11.5_* and Cu*_3.2_* CHA*_11.0_* are provided in Figure S2 (Supporting Information). In the initial reduced state, the spectrum is dominated by a rising‐edge feature at ≈8983.9 eV, corresponding to a dipole‐allowed 1s→4p_x,y_ transition in quasi‐linear Cu(I).^[^ ^19^, ^71^, ^72^, ^73^, ^74^ ^]^ Introduction of O_2_ at 240 K results in an instantaneous development of a small shoulder at ≈8986.8 eV, which has been attributed to a dipole‐allowed 1s→4p transition in Cu(II) combined with a shakedown charge transfer from a ligand p valence orbital into the metal 3d^9^ orbital.^[^ ^75^, ^76^, ^77^ ^]^ Simultaneously, a pre‐edge signal at ≈8977.5 eV emerges, which arises from a quadrupole‐allowed 1s→3d transition in Cu(II) situated within a distorted coordination environment.^[^ ^78^ ^]^ These spectral changes are indicative of the oxidation of Cu(I) into Cu(II), which starts at temperatures as low as 240 K. The extent of material oxidation increases upon raising the temperature, which is highlighted by the progressive amplification of the intensity of the signals at ≈8977.5 and 8986.8 eV at the expense of the feature at ≈8983.9 eV. This is accompanied by a rise of the white line signal at ≈8999.6 eV, suggesting an increase in the first shell coordination number.^[^ ^53^ ^]^

The development of the fraction of Cu(II) in the different materials throughout the temperature ramp (Figure 1b) has been determined by linear combination fitting (LCF). Details concerning the fitting procedure are illustrated in Figures S3 and S4 (Supporting Information). As already implied by the trends of the spectra in Figure 1a, the fraction of Cu(II) surges to 19, 25, and 12% in Cu*_3.2_* MOR*_10.0_*, Cu*_3.6_* MFI*_11.5_*, and Cu*_3.2_* CHA*_11.0_* upon contacting the reduced samples with O_2_ at 240 K and continues to grow when increasing the temperature. Notably, the gain in the fraction of Cu(II) at temperatures in the range from 240 to 370 K is significantly more steep in Cu*_3.2_* MOR*_10.0_* and Cu*_3.6_* MFI*_11.5_* than in Cu*_3.2_* CHA*_11.0_*. The framework‐dependent nature of the overall oxidizability is also highlighted by the temperature shift at the inflection point of the Cu(II) profiles above 300 K, which progresses in the order of Cu*_3.2_* MOR*_10.0_* (450 K) < Cu*_3.6_* MFI*_11.5_* (490 K) < Cu*_3.2_* CHA*_11.0_* (530 K). Figure 1b further demonstrates that the fraction of Cu(II) remains below unity in each of the samples during O_2_‐TPO. This is particularly noticeable in Cu*_3.2_* CHA*_11.0_*, where the maximal Cu(II) fraction amounts to only ≈52%. In contrast, the highest achievable Cu(II) fraction in Cu*_3.2_* MOR*_10.0_* and Cu*_3.6_* MFI*_11.5_* is ≈87%. This peculiar behavior suggests that the Cu(II) fraction of the individual samples does not match the one of the corresponding fully oxidized reference materials at any point of the O_2_‐TPO. The spectra of these references were acquired at room temperature after a cool‐down in the presence of the oxidant following a high‐temperature activation. Consequently, the incomplete Cu(I) re‐oxidation throughout O_2_‐TPO signifies a thermodynamically‐driven autoreduction of the Cu‐zeolites at elevated temperatures despite the presence of O_2_ (10 mbar). The temperature‐induced shift of the redox equilibrium toward autoreduction is additionally illustrated by the subsequent decline of the Cu(II) profile of each Cu‐zeolite after surpassing the maximal Cu(II) fraction, which is especially pronounced in Cu*_3.6_* MFI*_11.5_* and Cu*_3.2_* CHA*_11.0_*. Based on the development of the Cu(II) profiles, Cu*_3.2_* CHA*_11.0_* appears to be more prone to autoreduction than Cu*_3.2_* MOR*_10.0_* and Cu*_3.6_* MFI*_11.5_*. Noteworthy, Cu(II) autoreduction at high temperatures in the presence of O_2_ has also been reported by Andersen et al.^[^ ^79^ ^]^

**Figure 2:**
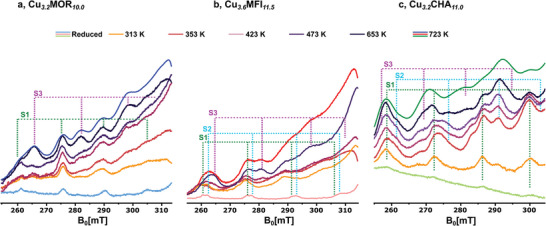
In situ cw X‐band EPR spectra of Cu_3.2_MOR_10.0_ a) Cu_3.6_MFI_11.5_ b) and Cu_3.2_CHA_11.0_ c) after reduction and re‐activation at different temperatures. A comparison between the spectra after a regular activation and re‐activation at 723 K is shown in Figure S17 (Supporting Information). All spectra were recorded at 293 K in vacuum. The hyperfine patterns of different Cu(II) species and their corresponding assignments are indicated. The line shape simulations are shown in Figures S6–S8 (Supporting Information).

**Figure** **2** illustrates the g_II_ region of the in situ continuous wave (cw) X‐band EPR spectra of Cu*_3.2_* MOR*_10.0_*, Cu*_3.6_* MFI*_11.5_*, and Cu*_3.2_* CHA*_11.0_* after the preceding reduction and the subsequent isothermal re‐activation at selected temperatures. EPR spectra of monomeric Cu(II) feature a characteristic hyperfine quartet, resulting from the coupling of the unpaired electron spin (S = 1/2, 3d^9^ configuration) with the Cu nucleus (I = 3/2). On the contrary, Cu(I) does not give rise to an EPR signal due to its closed shell 3d^10^ configuration. The hyperfine coupling constant as well as the g_II_ value are indicative of the coordination geometry as well as the number and type of the surrounding ligands.^[^ ^80^, ^81^, ^82^ ^]^ Therefore, monomeric Cu(II) species located within different ion exchange positions can be distinguished based on their distinct spectral patterns.^[^ ^83^, ^84^ ^]^ Dimeric Cu(II) centers are considered to be EPR invisible under the applied conditions due to the strong coupling of the metal centers.^[^ ^84^, ^85^ ^]^

Upon reduction in CH_4_ at 753 K, only a small fraction of Cu(II) persists in each of the Cu‐zeolites. Quantitative EPR shows that the residual fraction of Cu(II) amounts to 5%, 6%, and 8% of the total Cu amount in Cu*_3.2_* MOR*_10.0_*, Cu*_3.6_* MFI*_11.5_*, and Cu*_3.2_* CHA*_11.0_* (Figure S5, Supporting Information). The hyperfine patterns of the remaining Cu(II) sites are consistent with bare Cu^2+^ ions located in the vicinity of two Al T‐sites. The dominant species prevailing in Cu*_3.2_* MOR*_10.0_* exhibits a g_II_ and A_II_ value of 2.33 and 472 MHz, respectively (Figure S6, Table S4, Supporting Information). This center has been tentatively associated with bare Cu^2+^ located in a six‐MR where the Al T‐sites are arranged in a *para* (‐Al‐Si‐Si‐Al‐) configuration (S1).^[^ ^17^, ^83^, ^86^ ^]^ In reduced Cu*_3.6_* MFI*_11.5_*, two Cu(II) sites are distinguishable, which are characterized by g_II = _2.33 and A_II = _492 MHz as well as g_II = _2.31 and A_II = _498 MHz (Figure S7, Table S5, Supporting Information). The first species can be putatively assigned to S1, whereas the latter corresponds to bare Cu^2+^ adjacent to two Al T‐sites positioned in a *meta* (‐Al‐Si‐Al‐) sequence (S2).^[^ ^67^, ^86^ ^]^ In Cu*_3.2_* CHA*_11.0_*, a Cu(II) center can be identified with g_II = _2.35 and A_II = _460 MHz, arising from S1 (Figure S8, Table S6, Supporting Information).^[^ ^16^, ^87^ ^]^ Note that the spin Hamiltonian parameters of iso‐compositional Cu(II) motifs may still vary in different zeolite frameworks.^[^ ^80^ ^]^

After isothermal re‐activation at 313 K, the signal intensity of the features corresponding to bare Cu^2+^ centers increases in the spectra of all three samples. This is accompanied by the appearance of new features resulting from additional Cu(II) species. A complete data set showing the temporal evolution of the spectra is provided in Figures S9–S14 (Supporting Information). In Cu*_3.2_* MOR*_10.0_*, a center exhibiting a g_II_ and A_II_ value of 2.28 and 510 MHz becomes visible, which has been related to Cu(II) charge‐balanced by one framework O‐atom (O_f_) and an OH‐ligand in the form of [CuOH]^+^ (S3) (Figure S6, Table S4, Supporting Information).^[^ ^17^, ^83^, ^86^, ^88^ ^]^ Likewise, a site with g_II = _2.28 and A_II = _522 MHz can be identified in Cu*_3.6_* MFI*_11.5_*, which also originates from S3 (Figure S7, Table S5, Supporting Information).^[^ ^86^ ^]^ Furthermore, two new species can be detected in Cu*_3.2_* CHA*_11.0_*. The first one features a g_II_ and A_II_ value of 2.33 and 490 MHz and can be associated with S2, whereas the second one is characterized by g_II = _2.4 and A_II = _410 MHz and has been assigned to S3 (Figure S8, Table S6, Supporting Information).^[^ ^16^, ^87^ ^]^ It should be noted that an opposing assignment regarding S1 and S2 in Cu‐exchanged CHA has been suggested.^[^ ^80^, ^84^ ^]^ Fourier‐transform infrared (FTIR) spectra (Figure S15, Supporting Information) indicate an increase in Brønsted acid site (BAS) signal intensity after the preceding material reduction in CH_4_, suggesting BAS as a H^+^ source for S3. Notably, the spectra after re‐activation at increasing temperatures are characterized by a varying relative increase in signal intensity of specific Cu(II) centers. In particular, the formation of S3 in all materials and the generation of S2 in Cu*_3.2_* CHA*_11.0_* requires significantly higher temperatures compared to S1. After re‐activation at < 473 K, a spectral component ≈257 mT can be observed in the spectra of Cu*_3.2_* MOR*_10.0_* and Cu*_3.6_* MFI*_11.5_*, which corresponds to partially hydrated Cu(II) (Figure S16, Supporting Information). At higher re‐activation temperatures, this shoulder vanishes due to the apparent desorption of H_2_O. Throughout re‐activation at elevated temperatures (Figures S12–S14, Supporting Information), a slow decline of the overall signal intensity can be observed after prolonged reaction time, suggesting that monomeric Cu(II) species are marginally affected by autoreduction.^[^ ^15^, ^17^, ^88^ ^]^ Isothermal re‐activation at 723 K leads to an almost identical spectral line shape for all three materials compared to their spectra after a regular activation (Figure S17, Supporting Information). The fraction of detectable monomeric Cu(II) relative to the total Cu loading amounts to 47%, 68%, and 51% in Cu*_3.2_* MOR*_10.0_*, Cu*_3.6_* MFI*_11.5_*, and Cu*_3.2_* CHA*_11.0_* after a regular activation as shown by quantitative measurements (Figure S5, Supporting Information). The differences in Cu‐speciation highlight the distinct local Al T‐site arrangement within the parent commercial zeolites, which is governed by their specific synthesis method and the nature of the potentially employed post‐synthetic modifications.^[^ ^70^ ^]^ The particular experimental procedure can induce a shift in the ratios between certain Cu(II) sites, which in turn affects the redox properties of the individual materials.^[^ ^89^ ^]^

In order to trace the development of monomeric Cu(II) sites throughout the oxidative treatment, each sample was subjected to an O_2_‐TPO (Figures S18–S20, Supporting Information). Similar to the XANES‐derived Cu(II) profiles (Figure 1b), a rise in the signal intensity of Cu*_3.2_* MOR*_10.0_* and Cu*_3.6_* MFI*_11.5_* can already be observed in the temperature range from 200 to 300 K, implying that monomeric Cu(II) centers form at low temperature in these materials. On the contrary, the intensity increase in this regime is negligible in Cu*_3.2_* CHA*_11.0_*. The subsequent evolution of the signal intensity in the range from 300 to 753 K in Cu*_3.2_* MOR*_10.0_* and Cu*_3.6_* MFI*_11.5_* is very similar. A reliable determination of the evolution of the signal intensity in Cu*_3.2_* CHA*_11.0_* at temperatures above 300 K was not possible due to the appearance of a strongly superimposed and temperature‐dependent feature stemming from EPR‐visible Cu nanoparticles (Figure S21, Supporting Information). Notably, the evolution of the EPR‐based signal intensity of Cu*_3.2_* MOR*_10.0_* and Cu*_3.6_* MFI*_11.5_* deviates from the development of their corresponding XANES‐derived Cu(II) profiles, whose inflection points in the same temperature regime are separated by ≈40 K. This discrepancy may arise from the fact that exclusively monomeric Cu(II) species are traced by the EPR‐based O_2_‐TPO.

**Figure 3:**
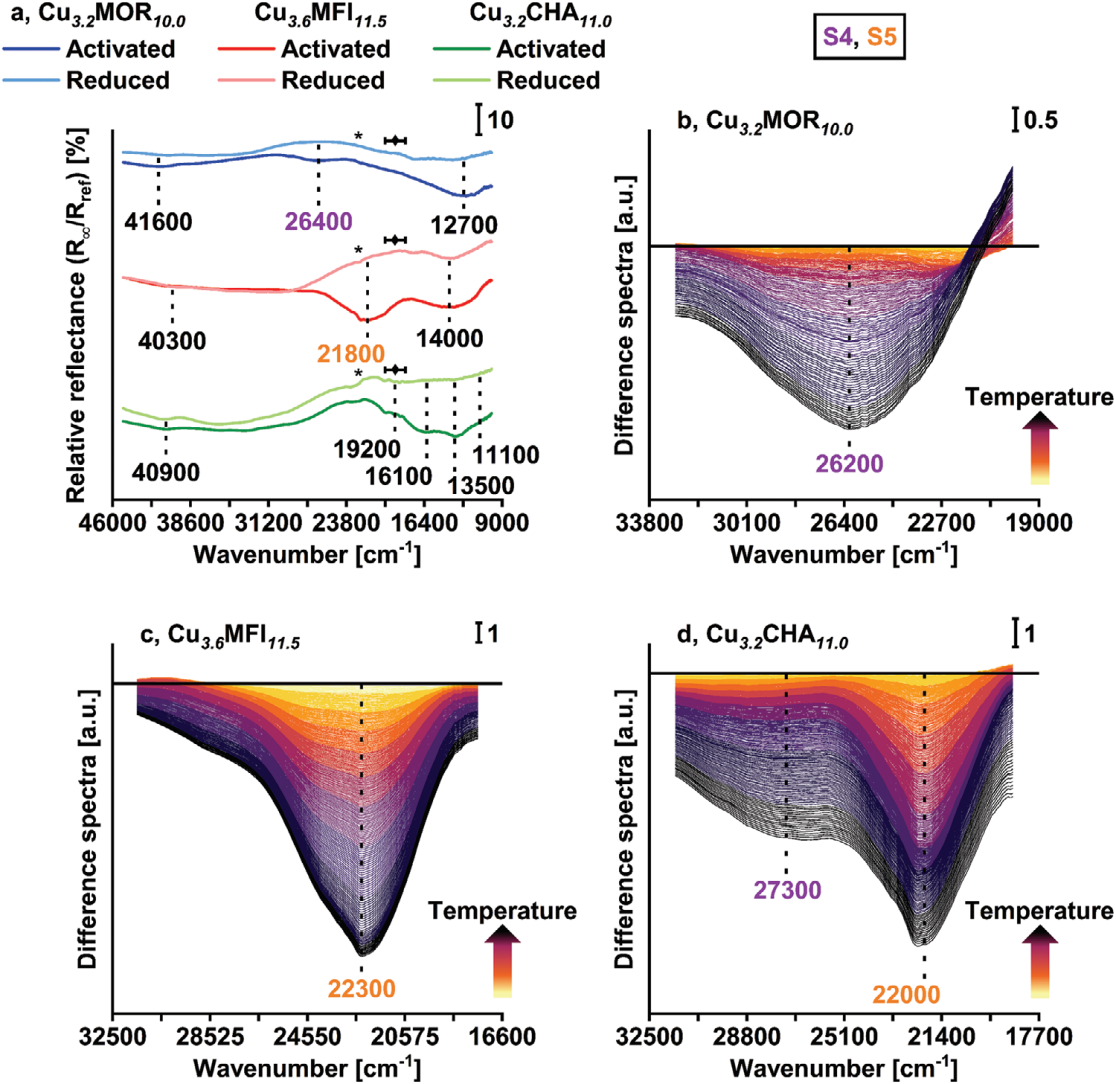
In situ UV–vis spectra of regularly activated (recorded at 365 K in vacuum) and reduced (recorded at 300 K in vacuum) Cu_3.2_MOR_10.0_, Cu_3.6_MFI_11.5_, and Cu_3.2_CHA_11.0_ (a). The characteristic bands of S4 and S5 are highlighted in purple and orange. The feature characterized by an inflection point at ≈22700 (indicated by *) arises from the deuterium/halogen lamp switch. Weak superimposed signals in the range from 20200 to 18500 cm^−1^ (indicated by 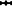) originate from a 3d^9^4s/4p→3d^10^ photoluminescence process in Cu(I).^[^ ^86^ ^]^ Spectra in the region of the characteristic O_ef_→Cu(II) LMCT transition of S4 and S5 in Cu_3.2_MOR_10.0_ (b), Cu_3.6_MFI_11.5_ (c), and Cu_3.2_CHA_11.0_ (d) during CH_4_‐TPR in the temperature range from 300 to 753 K. A spectral profile below zero (black solid line) indicates a loss in absorbance, whereas a gain in absorbance exhibits the opposite behavior.

**Figure** **3** depicts the in situ UV–vis spectra of the Cu‐zeolites after regular activation and reduction as well as during temperature‐programmed reduction with CH_4_ (CH_4_‐TPR). Spectra in Figure 3a are given in relative reflectance, while Figure 3b–d report difference spectra. The spectrum of activated Cu*_3.2_* MOR*_10.0_* (Figure 3a) displays an absorption band (local minima in relative reflectance) at ≈12700 cm^−1^, which predominantly originates from non‐resolved d‐d transitions in S1 and S2.^[^ ^17^, ^83^, ^90^, ^91^ ^]^ Electronic excitations between non‐degenerate d levels in S1 and S2 also give rise to the signal at ≈14000 cm^−1^ in the spectrum of activated Cu*_3.6_* MFI*_11.5_*.^[^ ^92^, ^93^, ^94^ ^]^ The broad nature of both features suggests additional contributions from other Cu(II) sites such as S3 and multimeric Cu‐oxo moieties. On the contrary, the spectrum of activated Cu*_3.2_* CHA*_11.0_* is characterized by four well‐resolved bands at ≈19200, 16100, 13500, and 11100 cm^−1^. Considering that these signals were found to be absent in the spectra of Cu‐exchanged CHA primarily hosting S1, S2, and S3, this quadruplet has been attributed to d‐d transitions in two types of [Cu_2_(µ‐O)]^2+^ centers (S4 and S5).^[^ ^16^, ^54^, ^70^ ^]^ Notably, the associated O_ef_→Cu(II) ligand‐to‐metal charge transfer (LMCT) transitions of S4 and S5 are not clearly evident in the spectrum of activated Cu*_3.2_* CHA*_11.0_*. Nevertheless, they become discernible in the spectra collected during CH_4_‐TPR (Figure 3d), yielding features at ≈27300 (S4) and 22000 cm^−1^ (S5). In contrast, two distinct features are present in the spectra of activated Cu*_3.2_* MOR*_10.0_* and Cu*_3.6_* MFI*_11.5_* at ≈26400 and 21800 cm^−1^, respectively. These bands can also be identified in the corresponding spectra acquired during CH_4_‐TPR (Figure 3b,c). The signal positions in relative reflectance and difference spectra do not necessarily need to precisely coincide. The former corresponds to an actual maximum in absorbance, whereas the latter indicates a change in absorbance relative to a specific reference spectrum. Importantly, both features have been attributed to a µ‐oxo 2p→Cu(II) 3d/4s charge transfer in different [Cu_2_(µ‐O)]^2+^ species characterized by different Cu–Cu distances.^[^ ^12^, ^14^, ^17^, ^20^, ^95^, ^96^ ^]^ Based on the similar spectral positions, it can be argued that the bands at 26400 and 21800 cm^−1^ in the spectra of Cu*_3.2_* MOR*_10.0_* and Cu*_3.6_* MFI*_11.5_* correlate to S4 and S5, respectively. Thus, two different [Cu_2_(µ‐O)]^2+^ motifs appear to be present in the activated Cu‐zeolites, whose relative proportions are likely governed by the distinct structural properties of the specific topologies as well as the characteristic Al T‐site distribution of the different zeolites. The fraction of S4 decreases in the order of Cu*_3.2_* MOR*_10.0_* > Cu*_3.2_* CHA*_11.0_* > Cu*_3.6_* MFI*_11.5_*, whereas the one of S5 decreases in the order of Cu*_3.6_* MFI*_11.5_* > Cu*_3.2_* CHA*_11.0_* > Cu*_3.2_* MOR*_10.0_*. At last, the spectra of all activated materials exhibit broad signals with ill‐defined local maxima in the range from 40300 to 41600 cm^−1^, arising from O_f_→Cu(II) LMCT transitions.^[^ ^97^ ^]^ As highlighted by Figure 3a, reduction results in a pronounced absorbance loss in the spectral range of the d‐d transitions due to the conversion of Cu(II) (3d^9^) into Cu(I) (3d^10^) as well as a diminished intensity of the O_ef_→Cu(II) LMCT transitions owing to a change in the nature of the frontier orbitals.^[^ ^59^, ^95^ ^]^

**Figure 4:**
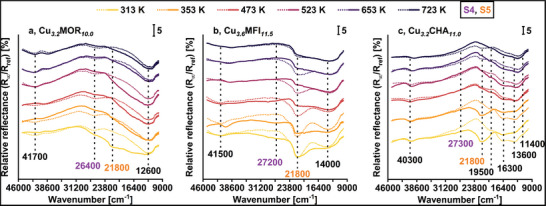
In situ UV–vis spectra of Cu_3.2_MOR_10.0_ a), Cu_3.6_MFI_11.5_ b), and Cu_3.2_CHA_11.0_ c) after a regular activation (dotted lines, recorded in O_2_) and isothermal re‐activation (solid lines, recorded in O_2_) at temperatures in the range from 313 to 723 K. Spectra of the activated and re‐activated materials were measured at the same specific temperature at which the corresponding re‐activation took place. The characteristic bands of S4 and S5 are highlighted in purple and orange.

**Figure** **4** summarizes the spectra of the materials after regular activation and isothermal re‐activation at specific temperatures. The full set of spectra is illustrated in Figure S22 (Supporting Information). The spectra of the activated samples recorded at 313 K closely resemble the ones displayed in Figure 3a. Noteworthy, an increase in the activation temperature causes a loss in the absorbance of the S4 and S5 O_ef_→Cu(II) LMCT transitions, which is particularly pronounced in the spectra of Cu*_3.2_* MOR*_10.0_* (Figure 4a) and Cu*_3.6_* MFI*_11.5_* (Figure 4b). In the latter case, this is accompanied with a decrease in intensity of the feature at ≈14000 cm^−1^, which moreover shifts to ≈13400 cm^−1^. Similarly, the absorbance of the quadruplet bands in the spectra of Cu*_3.2_* CHA*_11.0_* (Figure 4c) diminishes upon raising the activation temperature. Above 523 K, mostly an ill‐defined signal with a maximum at ≈12300 cm^−1^ remains in this zeolite. These transformations are indicative of a temperature‐induced autoreduction of S4 and S5, which takes place despite the presence of O_2_. This behavior is in good agreement with the fact that the XANES‐derived fractions of Cu(II) (Figure 1b) remain below unity. Moreover, it coincides with the reported exothermic nature of O_2_ adsorption on paired Cu(I) sites.^[^ ^35^, ^63^, ^65^ ^]^ As a result, the observed changes in the d‐d spectral range of Cu*_3.6_* MFI*_11.5_* and Cu*_3.2_* CHA*_11.0_* stem from a decrease in the fraction of dimeric Cu(II) sites compared to monomeric ones at high temperatures. In this context, it is important to mention that spectra of low Cu‐loaded MFI and CHA, which exclusively host S1, S2, and S3, are characterized by features at ≈13400 and 12100 cm^−1^.^[^ ^16^, ^92^ ^]^

Re‐activation at temperatures between 313 and 353 K gives rise to a significant increase in signal intensity in the range of the O_ef_→Cu(II) LMCT transitions of both S4 and S5 in each material. In certain cases, the absorbance at these positions is even higher than after a regular activation. The overall growth in absorbance is also apparent from the corresponding time‐resolved operando spectra (Figures S23–S25, Supporting Information) as well as the spectra acquired throughout the isothermal equilibration period in the O_2_‐TPO experiments (Figures S26–S31, Supporting Information). The collective formation of S4, as well as S5 in Cu_3.2_MOR_10.0_ and Cu_3.6_MFI_11.5_, opposes the situation after the regular activation, upon which only one of these centers is predominantly present (Figure 3a–c). The generation of the two Cu‐oxo centers is generally possible in both reduced samples at low temperatures. On the contrary, the creation of S4 and S5 in Cu*_3.2_* CHA*_11.0_* (Figure 4c) is in line with the observed intensity decrease of both of their O_ef_→Cu(II) LMCT transitions throughout CH_4_‐TPR (Figure 3d). Within the spectra of each Cu‐zeolite, the band originating from S5 is convoluted with a broad shoulder, which is shifted to higher wavenumbers. This signal could arise from a µ‐η^2^:η^2^‐peroxo $\pi_{\sigma }^{*}$→Cu(II) 3d LMCT transition in [Cu_2_(µ‐η^2^:η^2^‐O_2_)]^2+^, which has been reported to yield a feature at 29000 cm^−1^.^[^ ^64^ ^]^ This Cu(II) species has been proposed to act as a precursor for S4 and S5 via O─O bond cleavage upon being heated above 448 K. However, the spectra measured during re‐activation (Figures S23–S25, Supporting Information) and equilibration before O_2_‐TPO (Figures S26–S31, Supporting Information) indicate that the absorbance of bands stemming from S4, S5, and the potential intermediate increases rather simultaneously.

Similar to the impact of temperature on the spectra after regular activation, Figure 4 demonstrates that the intensity of signals deriving from the two Cu(II) dimers decreases with increasing the re‐activation temperature. This is also illustrated by the spectra of each material during the temperature ramp of the O_2_‐TPO (Figures S26–S31, Supporting Information). Importantly, the spectrum of Cu*_3.2_* MOR*_10.0_* after cooling in O_2_ following a modified O_2_‐TPO procedure (Figure S29, Supporting Information) reveals that the loss in the absorbance of the feature emerging from S4 is fully reversible. This behavior is distinctive for a temperature‐induced autoreduction. On the contrary, the intensity decline of the band assigned to S5 cannot be inverted, implying a decomposition of this site upon raising the temperature. The opposite occurs in Cu*_3.6_* MFI*_11.5_*, where only the absorbance of the signal resulting from S5 can be recovered after the cool‐down in O_2_ following a modified O_2_‐TPO (Figure S30, Supporting Information). The initial intensity of the feature corresponding to S4 can, however, not be restored. Thus, even though both dimeric Cu‐oxo centers can form in reduced Cu*_3.2_* MOR*_10.0_* and Cu*_3.6_* MFI*_11.5_* at low temperatures, their high‐temperature stability is framework‐dependent. S4 is particularly stabilized in Cu*_3.2_* MOR*_10.0_*, whereas S5 is predominantly sustained in Cu*_3.6_* MFI*_11.5_* after oxidative treatment at elevated temperatures. In Cu*_3.2_* CHA*_11.0_*, both Cu(II) dimers persist to a certain degree after this procedure.

Noteworthy, Figure 4 indicates that the loss in the absorbance of bands associated with temperature‐stable Cu(II) dimers is slightly greater after re‐activation at intermediate temperatures than after a regular activation. The extent of this disparity gradually decreases upon increasing the re‐activation temperature. A similar alternation in the intensity of signals caused by temperature‐stable S4 and S5 can also be observed in the spectra collected during the temperature ramp of the O_2_‐TPO (Figures S26–S31, Supporting Information). Here, the initial reduction in absorbance of their features is partially reversed when raising the temperature above ≈580 K. Hence, the observed intensity decline of bands arising from temperature‐stable dimeric Cu(II) species cannot exclusively originate from autoreduction. Considering the temperature‐dependent nature of the variation in absorbance, the additional process likely corresponds to an adsorption phenomenon. This is further emphasized by the fact that the intensity of signals associated with temperature‐stable dimeric Cu‐oxo centers is lower in the spectra of all samples after a cool‐down in O_2_ following a regular O_2_‐TPO than in the spectra after a regular activation (Figures S25–S27, Supporting Information). As mentioned before, their initial absorbance can be restored after a modified O_2_‐TPO procedure (Figures S28–S30, Supporting Information), which involves an additional high‐temperature evacuation and re‐activation step. Considering the appearance of the feature of partially hydrated Cu(II) in the EPR spectra (Figure 2), the excessive reduction in the intensity of signals ascribed to S4 and S5 after re‐activation at intermediate temperatures can be attributed to a combination of autoreduction and H_2_O adsorption. Indeed, the interaction of H_2_O with dimeric Cu(II) sites has been demonstrated to induce a decline in the absorbance of their O_ef_→Cu(II) LMCT transitions.^[^ ^16^, ^27^, ^98^ ^]^ Even more important, this process is completely reversible but requires remarkably high temperatures.^[^ ^98^ ^]^

A potential source of H_2_O could be the oxidation of hydrocarbon impurities during re‐activation and O_2_‐TPO. These residues may form throughout the initial high‐temperature reduction by CH_4_. Indeed, FTIR spectra recorded after material reduction show the presence of adsorbed surface species (Figure S34, Supporting Information). To further verify this hypothesis, Cu*_3.6_* MFI*_11.5_* was subjected to a regular O_2_‐TPO after reduction by CO (Figure S32, Supporting Information) since material treatment with CO should only yield CO_2_ instead of hydrocarbon deposits. Indeed, the absorbance of the band ascribed to S5 decreases monotonously throughout the temperature ramp in O_2_ and does not re‐increase upon surpassing ≈580 K. Moreover, the intensity of this band does not diminish when lowering the temperature again in O_2_, suggesting that no hydrocarbon residues were formed which could have been oxidized into CO_2_ and H_2_O.

**Figure 5:**
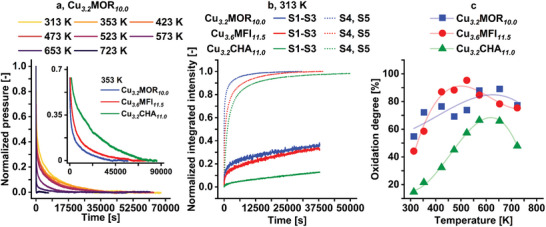
Normalized O_2_ pressure during isothermal re‐activation of Cu_3.2_MO_10.0_ in the temperature range from 313 to 723 K a). The inset shows the normalized O_2_ pressure during isothermal re‐activation of Cu_3.2_MOR_10.0_, Cu_3.6_MFI_11.5_, and Cu_3.2_CHA_11.0_ at 353 K. Normalized development of the integrated signal intensity of monomeric (solid lines) and dimeric (dotted lines) Cu(II) species in Cu_3.2_MOR_10.0_, Cu_3.6_MFI_11.5_, and Cu_3.2_CHA_11.0_ measured by operando EPR and UV–vis spectroscopy at 313 K b). Oxidation degree of Cu_3.2_MOR_10.0_, Cu_3.6_MFI_11.5_, and Cu_3.2_CHA_11.0_ after isothermal re‐activation at temperatures in the range from 313 to 723 K c). Transparent curves act as guides to the eye.

**Figure** **5**a depicts the normalized O_2_ pressure recorded during the isothermal re‐activation of Cu*_3.2_* MOR*_10.0_* at different temperatures. The normalized O_2_ isotherms of Cu*_3.6_* MFI*_11.5_* and Cu*_3.2_* CHA*_11.0_* are compiled in Figure S33 (Supporting Information). The temporal evolution of the normalized O_2_ traces highlights that the overall oxidation occurs at an exceptionally high rate. At 313 K, the half‐life of O_2_ ($t_{1/2}^{O_{2}}$), i.e., the time at which the normalized O_2_ pressure reaches 50% of its original value, amounts to just ≈400 s in Cu*_3.2_* MOR*_10.0_*. Upon increasing the temperature to 723 K, $t_{1/2}^{O_{2}}$ further declines to just ≈10 s. The fast pace of Cu‐zeolite oxidation has also been reported by Wieser et al. in a XANES‐based investigation of Cu*_4.7_* MAZ*_4.3_*.^[^ ^99^ ^]^ Upon contacting the reduced sample with O_2_ at 573 K, the authors noted a drop in the fraction of Cu(I) by ≈50% within the first ≈37 s. Moreover, a framework‐dependent increase in the rate of O_2_ consumption can be identified, which is demonstrated by the exemplary comparison of the normalized O_2_ isotherms of Cu*_3.2_* MOR*_10.0_*, Cu*_3.6_* MFI*_11.5_*, and Cu*_3.2_* CHA*_11.0_* during re‐activation at 353 K in the inset of Figure 5a. Here, $t_{1/2}^{O_{2}}$ decreases in the following sequence: Cu*_3.2_* CHA*_11.0_* (3940 s) > Cu*_3.6_* MFI*_11.5_* (1005 s) > Cu*_3.2_* MOR*_10.0_* (145 s). Similar trends are discernable at higher temperatures. Notwithstanding the non‐isothermal nature of the XANES‐based O_2_‐TPO experiments, this behavior is in agreement with the observed shift of the temperature at maximal Cu(II) formation rate (Figure 1b). Notably, the normalized O_2_ traces of each material are characterized by at least two different kinetic regimes as demonstrated by the fast initial decline of the normalized O_2_ pressure, which becomes less steep with progressing time.

The development of the normalized integrated signal intensity of monomeric and dimeric Cu(II) sites determined by EPR and UV–vis spectroscopy in the different Cu‐zeolites during isothermal re‐activation at 313 K is shown in Figure 5b. It is evident that the formation rate of Cu(II) dimers is considerably faster than the one of monomers. As a result, the initial quick decay of the normalized O_2_ pressure and its subsequent slower decline can be related to the generation of dimeric and monomeric Cu(II) sites, respectively. The operando UV–vis spectra of Cu*_3.2_* MOR*_10.0_* and Cu*_3.6_* MFI*_11.5_* in Figures S23 and S24 (Supporting Information) emphasize that the increase in the signal intensity of bands associated with either S4 or S5 becomes almost instantaneous at re‐activation temperatures greater than 423 K. This is less pronounced in the case of the spectra of Cu*_3.2_* CHA*_11.0_* (Figure S25, Supporting Information) due to the slower oxidation rate of this sample. In each material, the overall extent of the gain in absorbance is governed by the magnitude of the temperature‐induced autoreduction as well as the adsorption of H_2_O. Therefore, it appears that the creation of Cu(II) dimers, particularly in Cu*_3.2_* MOR*_10.0_* and Cu*_3.6_* MFI*_11.5_*, is primarily thermodynamically controlled. Moreover, the evolution of the normalized isotherms of both monomeric and dimeric Cu(II) species accelerates in the order of Cu*_3.2_* CHA*_11.0_* < Cu*_3.6_* MFI*_11.5_* < Cu*_3.2_* MOR*_10.0_*. Thus, the framework dependence of the overall oxidizability (Figure 5a) matches the temporal progression of the two classes of Cu(II) centers.

Figure 5c illustrates the oxidation degree of the three Cu‐zeolites after isothermal re‐activation at different temperatures. Considering that the reduction and bond cleavage of O_2_ is a four‐electron process, this parameter has been determined by:

(1) $$\mathrm{Oxidation}\;\mathrm{degree}=\left(\frac{4\;*\;N_{O_{2}}}{N_{\mathrm{Cu}}}\right)\;*\;100$$

Here, $N_{O_{2}}$ denotes the amount of substance of consumed O_2_, whereas *N*_Cu_ describes the Cu content of the samples as determined by elemental analysis (Table S1, Supporting Information). The oxidation degree of Cu*_3.2_* MOR*_10.0_* and Cu*_3.6_* MFI*_11.5_* is consistently higher than the one of Cu*_3.2_* CHA*_11.0_* throughout the studied temperature regime. Nevertheless, the oxidation degree of all materials remains below 100% and starts to decrease upon surpassing its maximum. The latter amounts to 85, 91, and 68% in Cu*_3.2_* MOR*_10.0_*, Cu*_3.6_* MFI*_11.5_*, and Cu*_3.2_* CHA*_11.0_*, respectively. These trends are in agreement with the ones of the XANES‐derived Cu(II) profiles and imply that Cu*_3.2_* CHA*_11.0_* suffers from a more extensive degree of temperature‐induced autoreduction than Cu*_3.2_* MOR*_10.0_* and Cu*_3.6_* MFI*_11.5_*. The minor discrepancies between the values obtained from the two experiments most likely arise from the transient nature of the XANES‐based O_2_‐TPO.

**Scheme 2:**
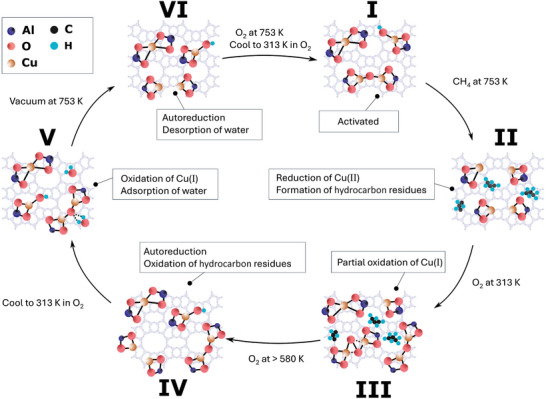
Illustration of the fate and interconversion of different Cu(II) species at various temperatures throughout the oxidative re‐activation procedure. The states (I–VI) of the material are explained in detail in the text. A summary is given in the boxes connected to the relevant states.

A summary of the processes occurring throughout oxidative re‐activation in the different Cu‐zeolites is depicted in **Scheme** **2**. Starting from the activated state (Scheme 2 I), exposure to CH_4_ at 753 K results in an almost complete reduction of Cu(II) to Cu(I) in each material (Scheme 2 II) as demonstrated by Figures 1, 2, 3. Figure S34 (Supporting Information) indicates that this is accompanied by the formation of hydrocarbon residues. Dosing O_2_ onto the reduced samples at temperatures as low as 240 K is sufficient to induce the oxidation of a fraction of Cu(I) (Figure 1b). Noteworthy, the subsequent increase in the amount of Cu(II) upon raising the temperature is more pronounced in Cu*_3.2_* MOR*_10.0_* and Cu*_3.6_* MFI*_11.5_* than in Cu*_3.2_* CHA*_11.0_*, implying that the oxidizability of the individual Cu(I) species hosted within the different Cu‐zeolites is framework‐dependent. This is further illustrated by the temperature at the inflection point above 300 K in Figure 1b, which decreases in the order of Cu*_3.2_* CHA*_11.0_* > Cu*_3.6_* MFI*_11.5_* > Cu*_3.2_* MOR*_10.0_*. Likewise, the rate of the O_2_ consumption of the different materials recorded during isothermal re‐activation at various temperatures declines in the reverse sequence (Figure 5a). The variations in the oxidation potential of the three samples might originate from differences in the stability of Cu(I) and Cu(II) species hosted within the distinct zeolite topologies. The preferential stabilization of Cu in different oxidation states is likely affected by the characteristic geometric properties of the distinction exchange positions and the specific distribution of the Al T‐sites therein.^[^ ^69^ ^]^ Interestingly, Cu(II) has indeed been found to be more stable in Cu‐MOR than in Cu‐MFI and Cu‐CHA.^[^ ^100^, ^101^, ^102^ ^]^ The diminished oxidizability of the Cu(I) sites hosted in Cu*_3.2_* CHA*_11.0_* could, in turn, indicate that the corresponding Cu(II) centers are characterized by a high reduction potential. It could be envisioned that this might relate to a higher activity of Cu*_3.2_* CHA*_11.0_* in the stepwise CH_4_‐to‐CH_3_OH conversion compared to Cu*_3.2_* MOR*_10.0_* and Cu*_3.6_* MFI*_11.5_*.^[^ ^102^ ^]^ Keeping in mind that material activation by O_2_ involves the generation of an O^2−^ moiety via O─O bond cleavage, the changes in the oxidizability of the materials may also arise from differences in the spatial density of the Cu sites. The same Cu(II) species, however in a different relative population and with varying high‐temperature stability, are identified in the distinct zeolite frameworks. The main role of the zeolite topology and structure is thus determining these characteristics.

At 313 K in O_2_ (Scheme 2 III), different monomeric and dimeric Cu(II) species as well as hydrocarbon deposits and Cu(I) centers coexist. S1 is the dominant monomeric Cu(II) site formed in all samples. Moreover, both S4 and S5 develop in each Cu‐zeolite at this temperature alongside the potential [Cu_2_(µ‐η^2^:η^2^‐O_2_)]^2+^ precursor (Figure 4).^[^ ^64^ ^]^ The simultaneous formation of the two dimeric Cu(II) species in Cu*_3.2_* MOR*_10.0_* and Cu*_3.6_* MFI*_11.5_* is surprising considering that only one of these Cu(II) centers is predominantly present in those materials after a regular activation. The evolution of the normalized signal intensity of monomeric and dimeric Cu(II) sites throughout re‐activation at 313 K shows that the formation of S4 and S5 is significantly faster than the one of S1, S2, and S3 (Figure 5b). Consequently the initial steep decrease and the subsequent slower decline of the normalized O_2_ isotherms are correlated to the generation of Cu(II) dimers and monomers, respectively. Remarkably, the formation rate of dimeric and monomeric Cu(II) species exhibits the same framework dependency as the rate of the overall O_2_ consumption. Based on the rapid formation of S4 and S5, their emergence at temperatures below 313 K can be assumed as highlighted by Figure 1a.

Increasing the temperature in O_2_ results in a continuous rise in the amount of S1. This is accompanied by the pronounced development of S3 in each sample as well as the distinct formation of S2 in Cu*_3.2_* CHA*_11.0_*. BAS arising from the preceding material reduction in CH_4_ likely acts as a source of H^+^ for the formation of S3 (Figure S15, Supporting Information). On the contrary, the generation of Cu(II) dimers is subjected to a progressively increasing degree of temperature‐induced autoreduction. This is evident from the diminishing signal intensity of S4 and S5 at elevated (re‐)activation temperatures. Further evidence is provided by Figures 1b and 5c, which demonstrate that the Cu(II) fraction and the oxidation degree of the Cu‐zeolites after re‐activation always remain below unity. Notably, the maximal Cu(II) fraction and oxidation degree of Cu*_3.2_* MOR*_10.0_* and Cu*_3.6_* MFI*_11.5_* are greater than those of Cu*_3.2_* CHA*_11.0_*, indicating that the extent of autoreduction in the latter material is more severe than in the former two samples. Cu(II) monomers are only marginally affected by autoreduction (Figures S12–S14, Supporting Information), whereas the tendency of dimeric Cu(II) centers toward autoreduction is substantially higher.^[^ ^17^, ^103^ ^]^

An increase in temperature initiates another parallel process, namely the gradual oxidation of the hydrocarbon residues, yielding CO_2_ and H_2_O. Owing to the high redox activity of dimeric Cu(II) centers, these deposits are primarily converted by S4 and S5.^[^ ^14^, ^16^, ^17^ ^]^ The latter are rapidly re‐oxidized due to their fast formation rate. However, a direct oxidation of hydrocarbon residues by O_2_ or via the participation of monomeric Cu(II) sites cannot be excluded. Below ≈580 K, the generated H_2_O adsorbs on the Cu(II) dimers, causing a decrease in the intensity of their characteristic O_ef_→Cu(II) LMCT transitions. As a result, the loss in absorbance of the features originating from S4 and S5 after re‐activation at intermediate temperatures does not only originate from autoreduction but also the interaction of H_2_O with these Cu(II) species. Raising the temperature above 580 K (Scheme 2 IV) triggers the desorption of H_2_O from the dimeric Cu(II) centers, which restores the signal intensity of their corresponding bands. Under these conditions, the absorbance of signals arising from S4 and S5 is solely governed by the extent of autoreduction.

The latter can be reversed by cooling the Cu‐zeolites in O_2_ to 313 K (Scheme 2 V) but the reoccurring adsorption of H_2_O masks the characteristic O_ef_→Cu(II) LMCT of S4 and S5 as evident from Figures S26–S28 (Supporting Information). Therefore, a subsequent evacuation at 753 K (Scheme 2 VI) is necessary to dehydrate the materials, which, however, leads to the autoreduction of S4 and S5 (Figures S29–S31, Supporting Information). The oxidized Cu(II) species are recovered by treating the samples in O_2_ again, which restores the activated state after a cool‐down in O_2_ to 313 K (Scheme 2 I). After this procedure, Cu*_3.2_* MOR*_10.0_* predominantly features the band emerging from S4, whereas Cu*_3.6_* MFI*_11.5_* mainly displays the signal stemming from S5. It appears that even though both Cu(II) dimers can initially form in the two samples during re‐activation at low temperatures, their high‐temperature stability is controlled by the zeolite topology.

The fact that the formation of dimeric Cu(II) centers does not require high activation temperatures and/or long activation durations, as frequently stated invites the question of why CH_3_OH productivity usually benefits from increasing both parameters.^[^ ^25^, ^39^ ^]^ Considering the adverse effect of adsorbed H_2_O on the intensity of features arising from S4 and S5, extended treatments in flowing O_2_ may be necessary to fully dehydrate the Cu‐zeolite. Importantly, the adsorption of H_2_O does not only mask the characteristic O_ef_→Cu(II) LMCT of S4 and S5 but also renders them inactive toward CH_4_.^[^ ^104^ ^]^ Moreover, long treatment periods in O_2_ at elevated temperatures facilitate the formation of monomeric Cu(II) centers due to their slower formation rate. Keeping in mind that S1, S2, and especially S3 have been proven to participate in partial CH_4_ oxidation, maximizing their fraction by an extensive activation protocol should enhance the CH_3_OH productivity.

## 3 Conclusion

The evolution of Cu(II) active sites for the CH_4_‐to‐CH_3_OH conversion has been studied in a series of Cu‐zeolites with different topologies but similar Si/Al ratios and Cu loadings, employing a combination of in situ and operando spectroscopic techniques, including XANES, EPR, and UV–vis spectroscopy. The oxidative generation of distinct Cu(II) species depends on their structure and the topology of the zeolite host. Bare Cu^2+^ sites start to emerge at 200 K, whereas a pronounced development of [CuOH]^+^ requires considerably higher temperatures. Two types of [Cu_2_(µ‐O)]^2+^ already appear at 313 K and their formation rate is significantly faster compared to the one of Cu(II) monomers. The relation between the oxidation kinetics of monomeric/dimeric Cu(II) sites and the zeolite topology is reflected in the overall oxidizability of the individual samples. The latter increases in the order of Cu*_3.2_* CHA*_11.0_* < Cu*_3.6_* MFI*_11.5_* < Cu*_3.2_* MOR*_10.0_*. Additionally, the zeolite framework and the structure of the Cu(II) motif govern its stability and fraction. Dimeric Cu(II) centers are found to be more prone to temperature‐induced autoreduction in O_2_ than monomeric ones. Moreover, the reversibility of this process is controlled by the topology. The autoreduction at high temperatures is accompanied by the transient reduction of [Cu_2_(µ‐O)]^2+^ by hydrocarbon residues arising from the initial reduction of the material in CH_4_. The oxidative decomposition of C_X_H_Y_ species yields CO_2_ and H_2_O. Below ≈580 K, the latter adsorbs on the Cu(II) dimers masking their characteristic UV–vis signatures and rendering them inactive in partial CH_4_ oxidation. This highlights that elevated activation temperatures are not required to generate the highly active [Cu_2_(µ‐O)]^2+^ species, but instead are necessary to efficiently dehydrate the material and to facilitate the formation of monomeric Cu(II) active sites.

## Conflict of Interest

The authors declare no conflict of interest.

## Supporting information



Supporting Information

## Data Availability

The data that support the findings of this study are available from the corresponding author upon reasonable request.
