# Supplementary material for: Tracking Active Site Formation during Oxidative Activation of Copper‐Exchanged Zeolites for Methane‐to‐Methanol Conversion
Source: Adv Sci (Weinh). 2025 Feb 14;12(13):2413870. doi: 10.1002/advs.202413870 (PMC11967819; doi:10.1002/advs.202413870)
Supplement: Supplementary file 1 — Supporting Information [file ADVS-12-2413870-s001.docx]

Tracking Active Site Formation During Oxidative Activation of Copper-Exchanged Zeolites for Methane-to-Methanol Conversion

Andreas Brenig,^+[a,b]^ Jörg W. A. Fischer,^+[c]^ Daniel Klose,^[c]^ Gunnar Jeschke,*^[c]^ Jeroen A. van Bokhoven,*^[a,b]^ Vitaly L. Sushkevich*^[b]^

[a] A. Brenig, Prof. Dr. J. A. van Bokhoven
Institute for Chemical and Bioengineering
ETH Zurich
Vladimir-Prelog-Weg 1-5/10, 8093 Zurich (Switzerland)

[b] A. Brenig, Prof. Dr. J. A. van Bokhoven, Dr. V. L. Sushkevich
Paul Scherrer Institute, Center for Energy and Environmental Sciences
Forschungsstrasse 111, 5232 Villigen (Switzerland)

[c] J. W. A. Fischer, Dr. D. Klose, Prof. Dr. G. Jeschke
Institute for Molecular Physical Science
ETH Zurich
Vladimir-Prelog-Weg 1-5/10, 8093 Zurich (Switzerland)

[+] These authors contributed equally to this work.

E-mail: gunnar.jeschke@phys.chem.ethz.ch, jeroen.vanbokhoven@chem.ethz.ch, vitaly.sushkevich@psi.ch

Table of Contents

| 1. Experimental Procedures | 2 |
| --- | --- |
| 1.1 Sample Preparation and Physiochemical Characterization | 2 |
| 1.2 In Situ X-ray Absorption Near Edge Structure (XANES) Spectroscopy | 2 |
| 1.3 Operando/In Situ and Quantitative Electron Paramagnetic Resonance (EPR) Spectroscopy | 3 |
| 1.5 Operando/In Situ Ultraviolet-Visible (UV-Vis) Spectroscopy | 4 |
| 1.6 In Situ Fourier-transform Infrared (FTIR) Spectroscopy | 6 |
| 2. Results and Discussion | 7 |
| 2.1 Elemental Analysis and Physiochemical Characterization | 7 |
| 2.2 In Situ XANES Spectroscopy | 7 |
| 2.3 Operando/In Situ and Quantitative EPR Spectroscopy | 9 |
| 2.5 Operando/In Situ UV-Vis Spectroscopy | 17 |
| 2.6 In Situ FTR Spectroscopy | 27 |
| 3. References | 27 |
| 4. Author Contributions | 27 |

1. Experimental Procedures

**1.1 Sample Preparation and Physicochemical Characterization**

Cu-exchanged MOR and MFI were prepared by aqueous ion exchange of commercial NH_4_-MOR (Zeolyst International, CBV 21A, Si/Al ratio: 10.0) and NH_4_-MFI (Zeolyst International, CBV 2314, Si/Al ratio: 11.5) using Cu(NO_3_)_2_•3H_2_O (Sigma-Aldrich, puriss. p.a., 99-104%). Approximately 5 g of the parent zeolite was stirred in 1 L of a ~0.05 M aqueous solution of Cu(NO_3_)_2_ for 24 h at 323 K, filtered, washed twice with 500 mL of deionized water, and dried for 2 h at 393 K. In total, this procedure was repeated three times in order to maximize the Cu loading. Cu-CHA was synthesized in a similar manner using a commercial H-CHA (Clariant, HCZC22, Si/Al
ratio: 11.0) sample. Prior to Cu introduction, about 3 g of the material was converted into the Na-form via a three-fold ion exchange in 600 mL of a ~0.15 M aqueous solution of CH_3_COONa (Carl Roth, 99%) according to the method described above. After a subsequent three-fold ion exchange in 600 mL of a 0.15 M NH_4_NO_3_ (Sigma-Aldrich, ≥99.0%) aqueous solution, the material was Cu-exchanged three times in 600 mL of a ~0.06 M aqueous solution of Cu(NO_3_)_2_ following the above mentioned procedure. Cu-zeolites are labelled as Cu*_X_*ZEO*_Y_*, where *_X_*, ZEO, and *_Y_* correspond to the Cu loading in wt%, the zeolite topology, and the Si/Al ratio.

The Cu and Na content of the prepared samples was determined by inductively coupled plasma-mass spectrometry (ICP-MS) using an Agilent 77009 instrument after sample digestion in HF in a 3000 Anton Paar microwave digestion system. N_2_ adsorption isotherms at 77 K were recorded using a Micromeritics 3DFlex surface characterization unit. Prior to N_2_ physisorption, the materials were dehydrated at 623 K under vacuum for at least 24 h. Powder X-ray diffraction (PXRD) patterns in the range from 5 to 70° two-theta were acquired on a Bruker D8 diffractometer using Cu K_α_ radiation with a wavelength of 1.5456 Å.

**1.2 In Situ X-ray Absorption Near Edge Structure (XANES) Spectroscopy**

In situ Cu K-edge XANES measurements were performed at BM31 of the Swiss-Norwegian Beamlines (SNBL) at the European Synchrotron Radiation Facility (ESRF), Grenoble, France. Around 5 mg of the sample was packed in a 0.8 mm diameter thin-walled (0.01 mm) quartz capillary reactor and held in position with a quartz wool plug. The quartz capillary was attached to the same batch reactor setup used for the EPR experiments, which is described elsewhere.^[1]^ A thermocouple was directly inserted into the sample bed to record the temperature during the temperature-programmed oxidation studies in O_2_ (O_2_-TPO). Sample cooling was achieved with a cold N_2_ vapor jet (Cryostream, Oxford Cryosystems). Sample heating was performed with a custom-made heating block, enabling control of the temperature. The X-ray beam, sample, and X-ray detectors were arranged in a conventional transmission geometry. Cu K-edge XANES spectra in the range from 8910 to 9120 eV were collected every 10 s. The extended X-ray absorption fine structure (EXAFS) part was not measured. Prior to the O_2_-TPO, each material was first subjected to a pretreatment procedure consisting of a dehydration step at 753 K (20 K/min) under vacuum for 20 min, a calcination period at 753 K in ~500 mbar O_2_ for 30 min, and a second dehydration phase at 753 K under vacuum for 10 min. This was followed by a reduction of the Cu-zeolite at
753 K in approximately 500 mbar CH_4_ for 30 min and a subsequent evacuation period at the same temperature for 1 h. Next, the sample was cooled down to 240 K under vacuum and 10 mbar O_2_ was introduced into the cell. The sample was then heated to 753 K (6 K/min). Upon reaching room temperature, the cryostream was exchanged for the heating block, which required a brief repositioning of the Cu-zeolite. A summary of the experimental procedure can be found in Scheme S1.


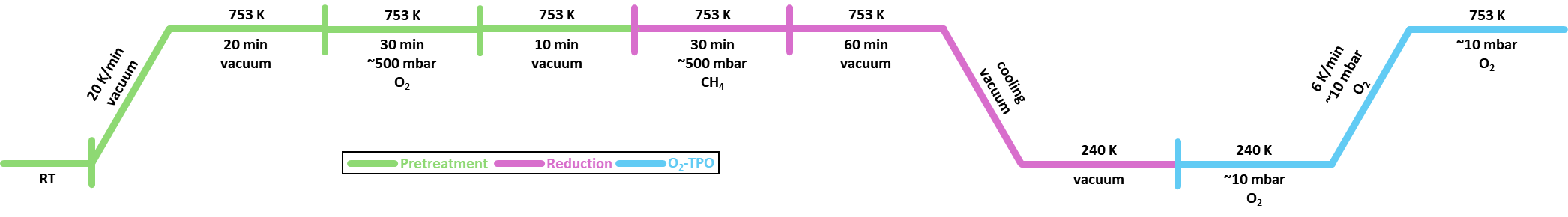


**Scheme S1.** Experimental procedure of the XANES O_2_-TPO.

**1.3 Operando/In Situ and Quantitative Electron Paramagnetic Resonance (EPR) Spectroscopy**

Operando and in situ continuous-wave (cw) EPR spectroscopy experiments in the temperature range between 300-753 K were performed using a home-built, water-cooled, high-temperature resonator installed into a X-band EPR spectrometer (Bruker EMX) operating at ~9.16 GHz.^[1,2]^ In situ cw EPR spectroscopy experiments in the temperature range between 200-300 K were conducted with an Elexsys E500 EPR spectrometer (Bruker Biospin) equipped with a liquid N_2_ cooling unit (Bruker Biospin) and a Super-High-Q resonator (Bruker Biospin) operating at ~9.5 GHz. Approximately 20 mg of the sieved material (250-500 µm) was inserted into an EPR quartz tube (Wilmad, OD: 4 mm) and fixed with a quartz wool plug. The quartz tube was subsequently connected to a home-built reactor setup.^[1]^

Prior to any measurement, each sample was first subjected to a onetime pretreatment procedure, consisting of a dehydration step at 753 K under vacuum for 2 h, a calcination period at 753 K in ~500 mbar O_2_ (Linde, grade: 5.0) for 2 h, and a second dehydration phase at 753 K under vacuum for 1 h. This was followed by a reduction step at 753 K in ~400 mbar CH_4_ (Messer, grade: 4.5) for 1 h and a subsequent evacuation period at the same temperature for 2 h. Cu-zeolites were kept within the EPR setup under vacuum in between experiments. After cooling the material to 200 K under vacuum, O_2_-TPO studies were conducted by dosing about 10 mbar O_2_ into the system while simultaneously registering spectral changes. The temperature was then raised to 300 K (6 K/min). After reaching 300 K, the setup was transferred to the other spectrometer and inserted back into the high-temperature cavity. The heating was then continued to 753 K (~6 K/min). A summary of the experimental procedure can be found in Scheme S2.


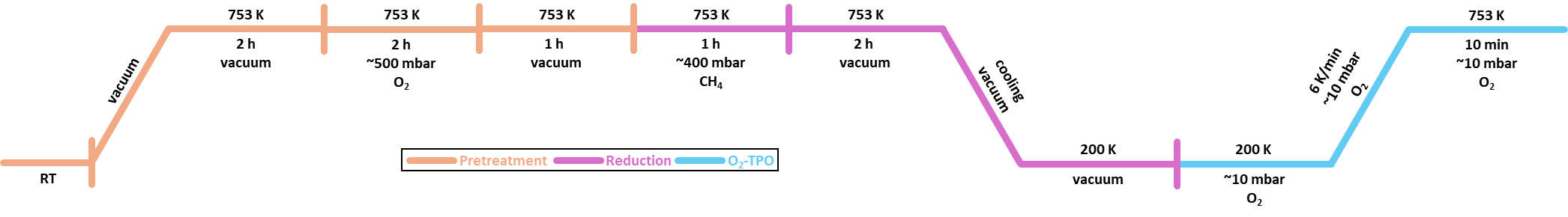


**Scheme S2.** Experimental procedure of the EPR O_2_-TPO.

For the isothermal re-activation experiments, each fresh sample was first activated once. The procedure consists of a dehydration step in vacuum at 753 K for 2 h followed by a calcination in ~400 mbar O_2_ for 2 h at the same temperature. This step was followed by an additional evacuation at 753 K for 1 h and a second treatment in 400 mbar O_2_ for 2 h. The sample was then cooled down in O_2_ to room temperature and vacuum was applied. The reference spectrum of the material after the regular activation was collected at this point. A summary of the experimental procedure can be found in Scheme S3.


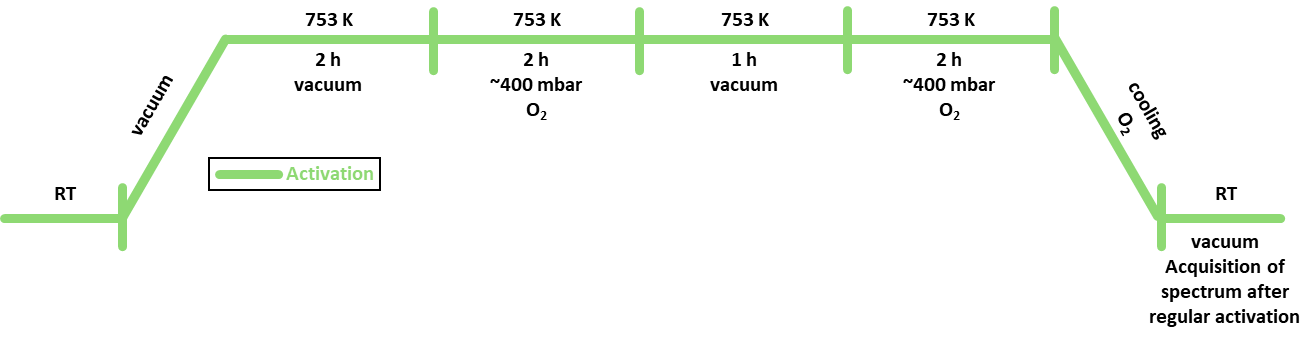


**Scheme S3.** Onetime activation of the samples prior to the isothermal re-activation.

After the regular activation procedure, the Cu-zeolite was heated in vacuum to 753 K and reduced in ~400 mbar CH_4_ for 1 h. Next, the material was treated in vacuum for 2 h and cooled down in vacuum to collect the reference spectrum of the reduced sample at room temperature. Afterwards, the Cu-zeolite was heated to the desired temperature in the range from 313 to 723 K and exposed to 10 mbar O_2_ while recording spectral changes as well as changes in the pressure. The material was then cooled down to room temperature and a spectrum of the re-activated sample was acquired after applying vacuum. Subsequently, the material was heated to 753 K in vacuum, exposed to ~400 mbar O_2_ for 2 h, and evacuated for 1 h. Prior to the next isothermal re-activation measurement, the material was reduced again in ~400 mbar CH_4_ at 753 K for 1 h and treated under vacuum at 753 K for 2 h. A summary of the experimental procedure can be found in Scheme S4.


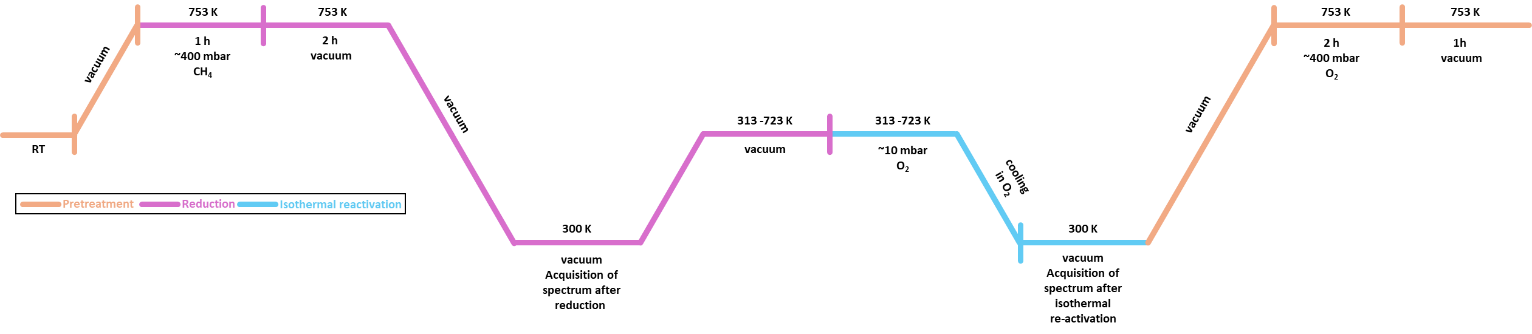


**Scheme S4.** Experimental procedure of the EPR isothermal re-activation experiments. The initial ramp from room temperature to 753 K in vacuum was performed only for the first isothermal re-activation after the regular activation procedure (see Scheme 3).

Quantitative X-band cw EPR measurements were conducted using Cu(pic)_2_:Zn(pic)_2_ Cu(II) references as employed by Fischer et al..^[1]^ The samples and references were measured on the same day without retuning the instrument. Special care has been taken to ensure that the reference and sample occupy the same volume within the quartz tube and that they were measured using quartz tubes with the same specifications.

The intensity of the cw EPR spectrum is proportionally related to the first derivative of the magnetic susceptibility of the sample at a certain temperature and at the external magnetic field strength. Accordingly, the double integral of the EPR spectrum is a direct measure of the total number of spins in the sample. The standard procedure to determine the concentration is double integration of the spectrum.^[3]^ Here the relative decay of the Cu intensity during O_2_ treatment was determined by double integration of the difference spectra at each point in time throughout the reaction. The intensity of the spectra recorded during O_2_-TPO were corrected for the temperature difference.^[4]^

**1.5 Operando/In Situ Ultraviolet-Visible (UV-Vis) Spectroscopy**

Operando and in situ UV-Vis measurements were performed using a home-built quartz reactor setup equipped with a custom UV-Vis-NIR Avantes six-around-one high-temperature reflection probe, an Ocean Optics DH-2000-BAL deuterium/halogen light source, and an Ocean Optics Maya 2000-Pro UV-Vis spectrometer.^[1]^ In a typical experiment, a square pellet with a mass of approximately
150 mg of sample was positioned in the quartz reactor cell above a reference white standard (corresponding parent zeolite in its NH_4_-form). Prior to any measurement, each sample was first subjected to a onetime pretreatment procedure consisting of a dehydration step at 753 K (10 K/min) under vacuum for 2 h, a calcination period at 753 K in ~500 mbar O_2_ (PanGas, grade: 5.0) for 2 h, and a second dehydration phase at 753 K under vacuum for 2 h. Materials were kept within the UV-Vis setup under vacuum in between experiments. A summary of the experimental procedure can be found in Scheme S5.


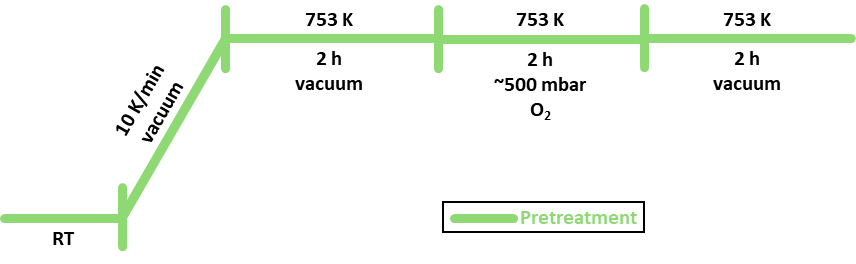


**Scheme S5.** Experimental procedure of the UV-Vis pretreatment procedure.

O_2_-TPO studies were conducted by heating the material to 753 K (10 K/min) under vacuum and subsequently activating it at 753 K in ~500 mbar O_2_ for 1 h. Next, the system was cooled to 365 K in O_2_ and a spectrum of the activated Cu-zeolite was acquired after a quick degassing. This procedure is referred to as "regular activation" in the main text. Following an evacuation at 753 K (10 K/min) for 1 h, the sample was reduced at 753 K in ~500 mbar CH_4_ (Linde, grade: 5.5) for 1 h and again evacuated at 753 K for 2 h. In particular cases, material reduction was performed in CO (Air Liquide, grade: 4.7) instead of CH_4_. After cooling the setup to 300 K under vacuum and recording a spectrum of the reduced Cu-zeolite, about 9.5 mbar O_2_ was introduced into the system while simultaneously registering spectral changes. The temperature was first maintained for 1 h, then raised to 753 K (6 K/min), kept constant for a specified amount of time, and finally decreased to 365 K. At this temperature, the setup was degassed again. The initial isothermal equilibration step was adopted since the UV-Vis spectrometer, unlike the EPR system, does not permit performing experiments below room temperature. Considering that the starting temperature in the EPR-based O_2_-TPO studies was 200 K, the isothermal O_2_ treatment in the UV-Vis-based measurements was employed to account for the additional time that the sample spent in contact with O_2_ at low temperatures. Additionally, each sample was subjected to a modified O_2_-TPO protocol. The latter included an additional evacuation step at 753 K for 1 h ensued by a treatment at 753 K in ~9.5 mbar O_2_ for 1 h after the initial dwell period at 753 K in O_2_ of the regular O_2_-TPO routine. This was concluded by a cool down to 365 K in O_2_ and a subsequent degassing. A summary of the experimental procedure can be found in Schemes S6 and S7.


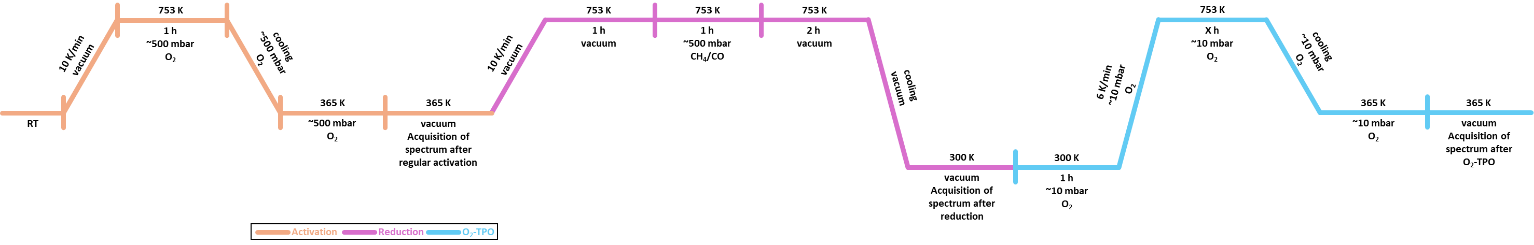


**Scheme S6.** Experimental procedure of the UV-Vis O_2_-TPO.


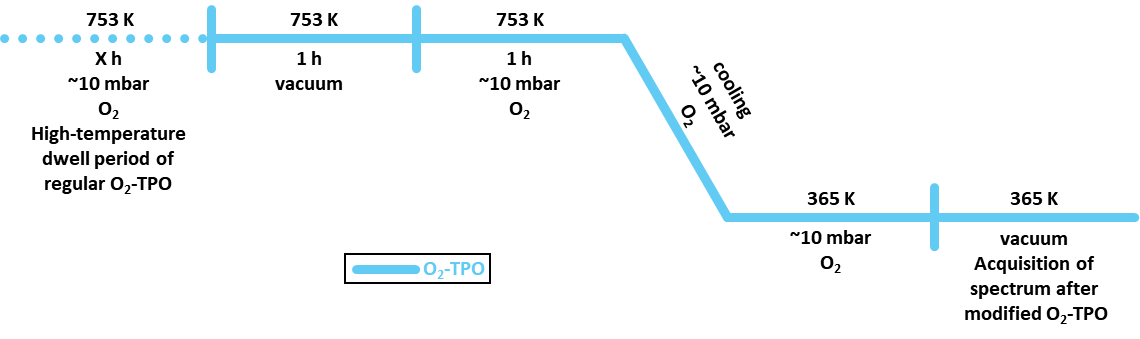


**Scheme S7.** Experimental procedure of the modified UV-Vis O_2_-TPO.

Isothermal re-activation experiments were carried out by heating the material to 753 K (10 K/min) under vacuum and subsequently activating it at 753 K in ~500 mbar O_2_ for 1 h. Next, the system was cooled in O_2_ to the specific temperature at which the isothermal re-activation would take place and a spectrum of the activated Cu-zeolite was acquired. This procedure is referred to as "regular activation" in the main text. Following an evacuation at 753 K (10 K/min) for 1 h, the sample was reduced at 753 K in ~500 mbar CH_4_ for 1 h and again evacuated at 753 K for 2 h. After cooling the setup to a particular temperature in the range from 313 to 723 K under vacuum and recording a spectrum of the reduced Cu-zeolite, about 9.5 mbar O_2_ was introduced into the system while simultaneously monitoring changes in the spectrum and the system pressure. The approximate duration of an isothermal re-activation experiment was 19 h. A summary of the experimental procedure can be found in Scheme S8.


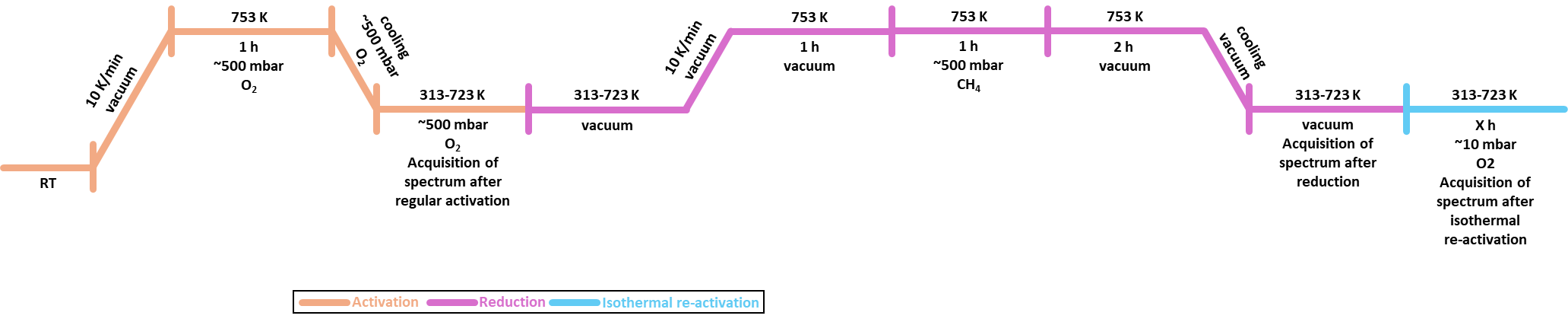


**Scheme S8.** Experimental procedure of the UV-Vis isothermal re-activation experiments.

Temperature-programmed reduction studies in CH_4_ were performed by heating the material to 753 K (10 K/min) under vacuum and subsequently activating it at 753 K in ~500 mbar O_2_ for 1 h. Next, the system was cooled to 365 K in O_2_, evacuated at 365 K for 30 min, and further cooled to 300 K under vacuum. While continuously recording spectra, ~9.5 mbar CH_4_ was introduced into the setup and the temperature was gradually raised to 753 K (6 K/min). A summary of the experimental procedure can be found in Scheme S9.


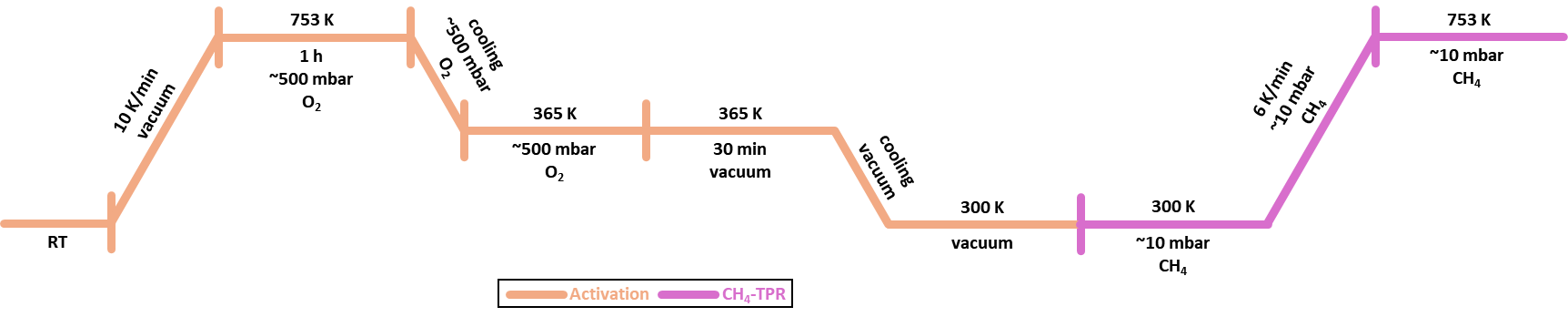


**Scheme S9.** Experimental procedure of the UV-Vis CH_4_-TPR.

Usually, 400 spectra were averaged and the spectrometer integration time was set to 25 ms. As a result, spectra were acquired with a total time resolution of about 10 s. A boxcar smoothing window with a width of eight points was applied. All UV-Vis spectra are given in relative reflectance instead of absorbance to avoid disproportionalities introduced by the usage of the Kubelka-Munk function.^[5]^

**1.6 In Situ Fourier-transform Infrared (FTIR) Spectroscopy**

In situ FTIR measurements were carried out on a Thermo Scientific Nicolet iS50 equipped with a deuterated-triglycine sulfate (DTGS) detector and a temperature-controllable quartz cell. Around 20 mg of the sample, which was pressed into a self-supporting wafer
(2 cm^2^), was used for a typical experiment. Prior to any measurement, each sample was first subjected to a pretreatment procedure consisting of a dehydration step at 753 K (10 K/min) under vacuum for 2 h, a calcination period at 753 K in ~365 mbar O_2_ (PanGas, grade: 5.0) for 2 h, and a second dehydration phase at 753 K under vacuum for 2h. Materials were kept within the FTIR setup under vacuum in between experiments.

FTIR measurements of adsorbed surface species after reduction in CH_4_ (Linde, grade: 5.5) were performed by heating the Cu-zeolite to 753 K (10 K/min) under vacuum and subsequently activating it at 753 K in ~365 mbar O_2_ for 1 h. After recording a spectrum of the activated sample (spectral resolution of 4 cm^-1^ and averaging of 128 scans), the system was evacuated at 753 K for 1 h followed by material reduction at 753 K in ~365 mbar CH_4_ for 1 h and a second dehydration at 753 K under vacuum for 2 h. At this point a spectrum of the remaining adsorbed surface species was collected. Data processing was performed using the OMNIC 9.5.9 software package.

2. Results and Discussion

**2.1 Elemental Analysis and Physicochemical Characterization**

**Table S1.** Composition of the prepared materials as well as their characteristic surface properties compared to the ones of the parent zeolites.

| Sample name  and composition ^[a]^ | Cu loading [wt%] | Na loading [wt%] | Surface area [m^2^/g] | Micropore volume [cm^3^/g] |
| --- | --- | --- | --- | --- |
| Cu*_3.2_*MOR*_10.0_* | 3.23 | 0.04 | 480 | 0.16 |
| H-MOR*_10.0_* | - | ND ^[b]^ | 490 | 0.16 |
| Cu*_3.6_*MFI*_11.5_* | 3.62 | 0.03 | 400 | 0.12 |
| H-MFI*_11.5_* | - | ND | 420 | 0.13 |
| Cu*_3.2_*CHA*_11.0_* | 3.20 | 0.05 | 700 | 0.24 |
| H-CHA*_11.0_* | - | ND | 740 | 0.25 |

[a] Samples are labelled as Cu*_X_*ZEO*_Y_*, where *_X_*, ZEO, and *_Y_* correspond to the Cu loading in wt%, the zeolite topology, and the Si/Al ratio.

[b] Not determined.

As indicated by Table S1, the specific surface area of the Cu-exchanged materials is lower than the one of the corresponding parent zeolites indicating a certain degree of pore blockage by Cu(II) species. Nevertheless, this does not significantly impact the micropore volume of the Cu containing samples since the former does not strongly deviate from the one of the related parent materials. A dominant CuO phase cannot be identified in the Cu-loaded zeolites since their PXRD patterns do not display additional reflections at 35.6 and 38.8° two-theta.^[6]^


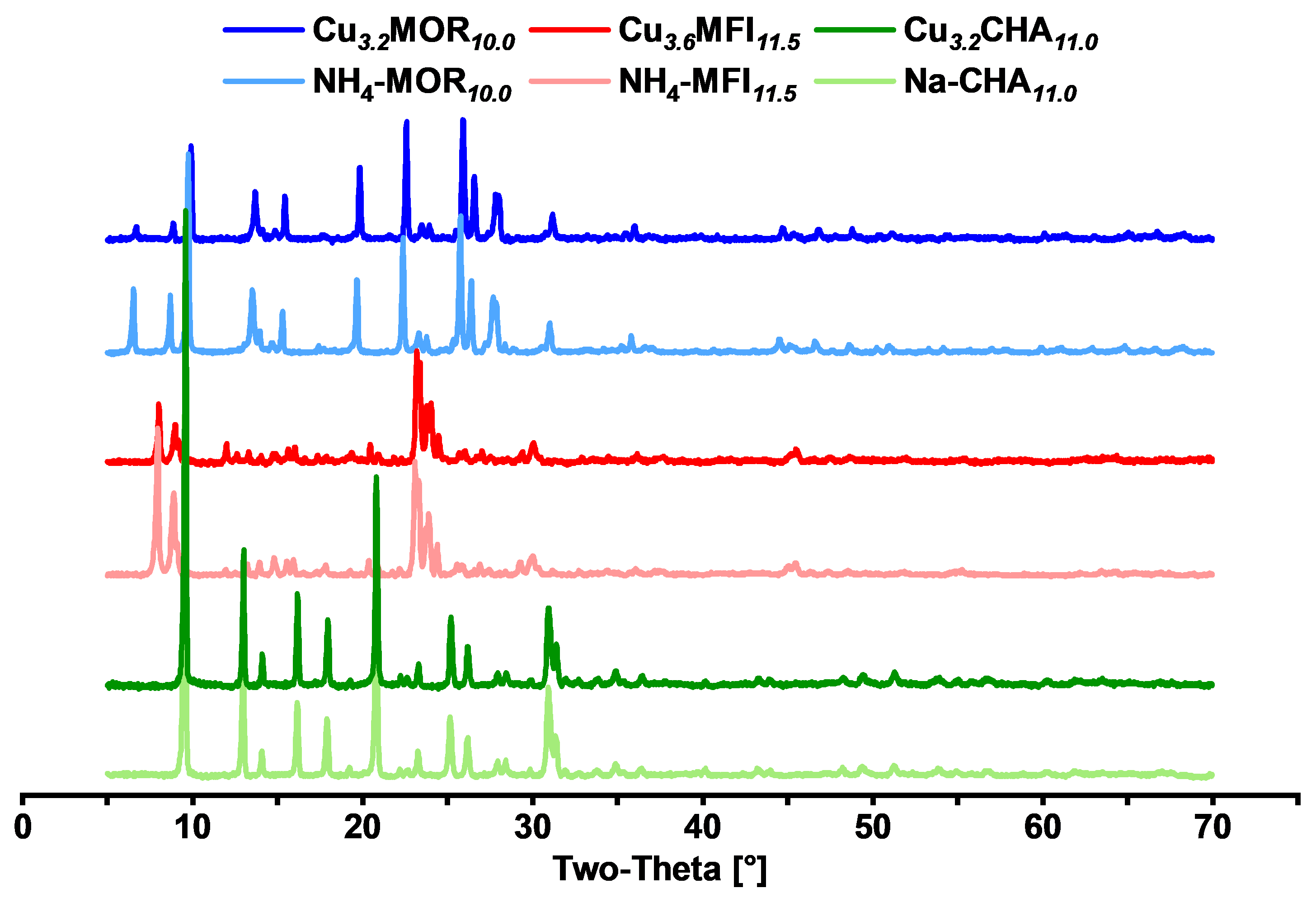


**Figure S1.** Ex situ PXRD patterns of Cu*_3.2_*MOR*_10.0_*, Cu*_3.6_*MFI*_11.5_*, and Cu*_3.2_*CHA*_11.0_* as well as the ones of the corresponding parent zeolites.

**2.2 In Situ XANES Spectroscopy**

**
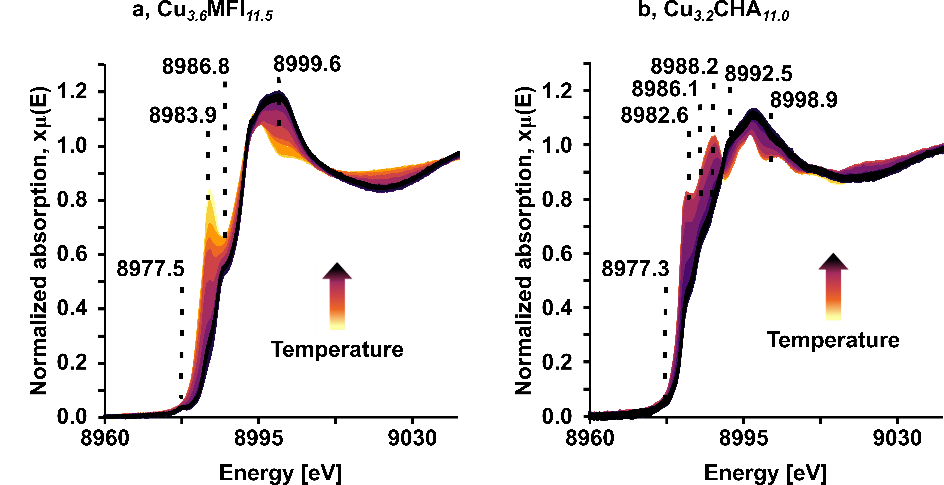
**

**Figure S2.** In situ Cu K-edge XANES spectra of Cu*_3.6_*MFI*_11.5_* (a) and Cu*_3.2_*CHA*_11.0_* (b) throughout O_2_-TPO.

After dosing O_2_ at low temperatures, the spectrum of Cu*_3.6_*MFI*_11.5_* is dominated by a feature at ~8983.9 eV, corresponding to the
1s→4p_x,y_ transition in Cu(I).^[7]^ This demonstrates that the majority of Cu is still present as Cu(I) at 240 K. Increasing the temperature leads to the appearance of a pre-edge feature around 8977.5 eV as well as a small shoulder at ~8986.8 eV, which originate from the dipole-allowed 1s→4p transition in Cu(II) and its quadrupole-allowed 1s→3d transition, respectively.^[7–9]^ During the O_2_-TPO an additional feature at ~8999.6 eV becomes visible, which is related to an increase in the first-shell coordination number.^[10]^

Similarly, the spectrum of Cu*_3.2_*CHA*_11.0_* is characterized by a feature at 8982.6 eV after the admission of O_2_ at low temperatures, which has been attributed to the dipole-allowed 1s→4p_x,y_ transition in Cu(I).^[11]^ This signal is accompanied by a feature at 8988.2 eV, which has been linked to a structurally different Cu(I) site.^[7,10,12]^ The presence of these signals highlight that the majority of Cu is present as Cu(I) at 240 K. Upon raising the temperature, a pre-edge signal at 8977.3 eV develops, which has been attributed to the dipole-forbidden but quadrupole-allowed 1s→3d transition in Cu(II).^[13]^ This band is accompanied with a rising-edge feature at 8986.1 eV, which has been assigned to the dipole-allowed 1s→4p transition in Cu(II).^[11,13]^ The two signals at 8992.5 and 8998.9 eV, appearing at higher temperatures in the white-line region, stem from bare Cu^2+^ in the six-membered ring (MR).^[13]^


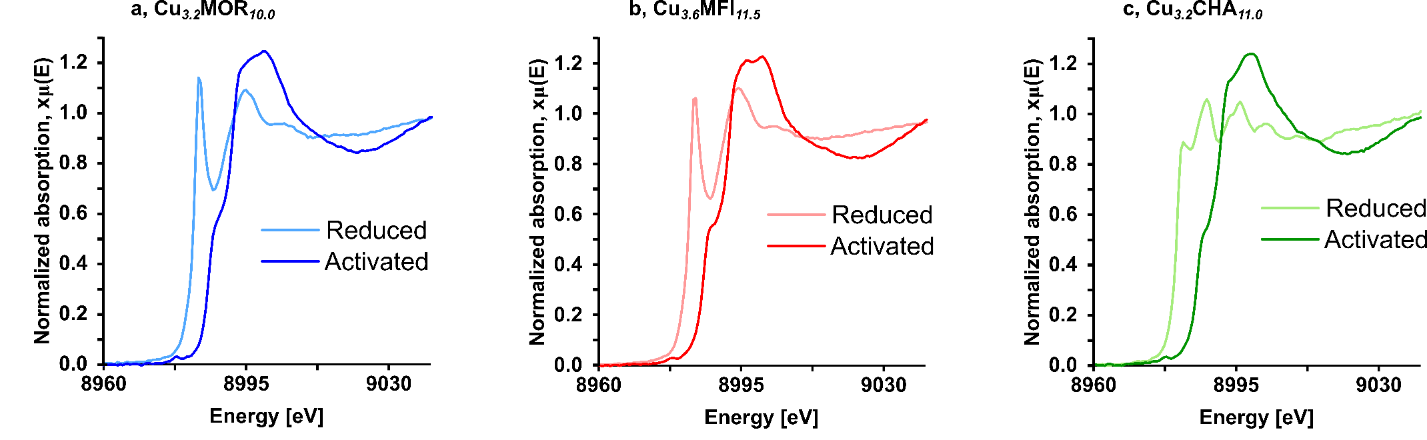


**Figure S3.** In situ Cu K-edge XANES spectra of regularly activated and reduced Cu*_3.2_*MOR*_10.0_* (a), Cu*_3.6_*MFI*_11.5_* (b), and Cu*_3.2_*CHA*_11.0_* (c). These spectra were used as references for the LCF procedure of the O_2_-TPO series.


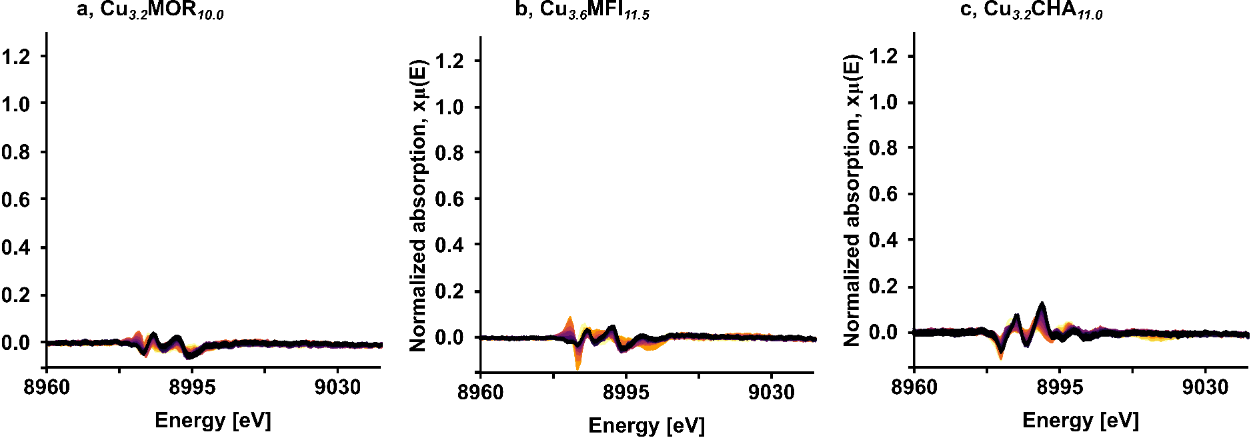


**Figure S4.** Residuals of the LCF procedure for Cu*_3.2_*MOR*_10.0_* (a), Cu*_3.6_*MFI*_11.5_* (b), and Cu*_3.2_*CHA*_11.0_* (c).

**2.3 Operando/In Situ and Quantitative EPR Spectroscopy**

The measurement parameters for the in situ and operando X-band measurements are listed in Table S2.

**Table S2.** Measurement parameters for the X-band EPR measurements.

| Experiment | Sweep  width [mT] | Modulation frequency [kHz] | Modulation amplitude [mT] | Sweep  time [s] | Conversion  time [ms] | Time  constant [ms] | Power  attenuation [dB] |
| --- | --- | --- | --- | --- | --- | --- | --- |
| Operando | 140 | 100 | 0.4 | 40 | 40 | 40 | 20 |
| In situ (200-300 K) | 110 | 100 | 0.2 | 42 | 40 | 40 | 20 |
| In situ (300-753 K) | 150 | 100 | 0.2 | 60 | 40 | 40 | 20 |
| In situ at room temperature | 200 | 100 | 0.3 | 327 | 80 | 20 | 20 |

The measurement parameters for the quantitative X-band EPR experiments are listed in Table S3. Note, that the error associated with the quantitative EPR measurements is about 5%.^[14]^

**Table S3.** Measurement parameters for the quantitative X-band EPR measurements at room temperature

|  | Sweep  width [mT] | Modulation  frequency [kHz] | Modulation  amplitude [mT] | Sweep  time [s] | Conversion time [ms] | Time constant [ms] | Power  attenuation [dB] |
| --- | --- | --- | --- | --- | --- | --- | --- |
|  | 200 | 100 | 3 | 163 | 80 | 40 | 24 |


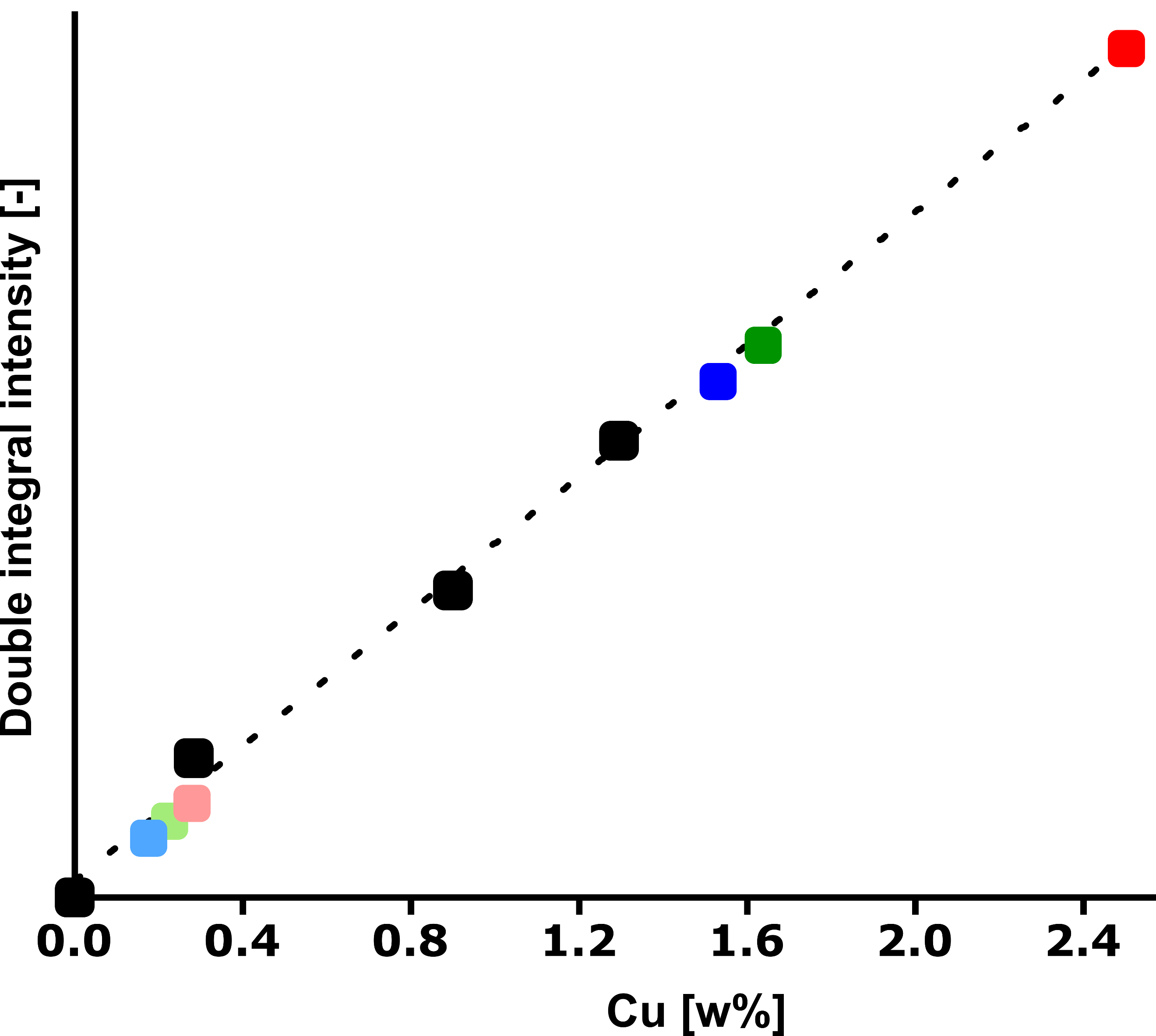


**Figure S5.** Quantitative EPR analysis of activated (vibrant) and reacted (pale) Cu*_3.2_*MOR*_10.0_* (blue), Cu*_3.6_*MFI*_11.5_* (red), and Cu*_3.2_*CHA*_11.0_* (green). All spectra were recorded at room temperature. Cu(pic)_2_:Zn(pic)_2_ was used as a Cu(II) standard (black).

**
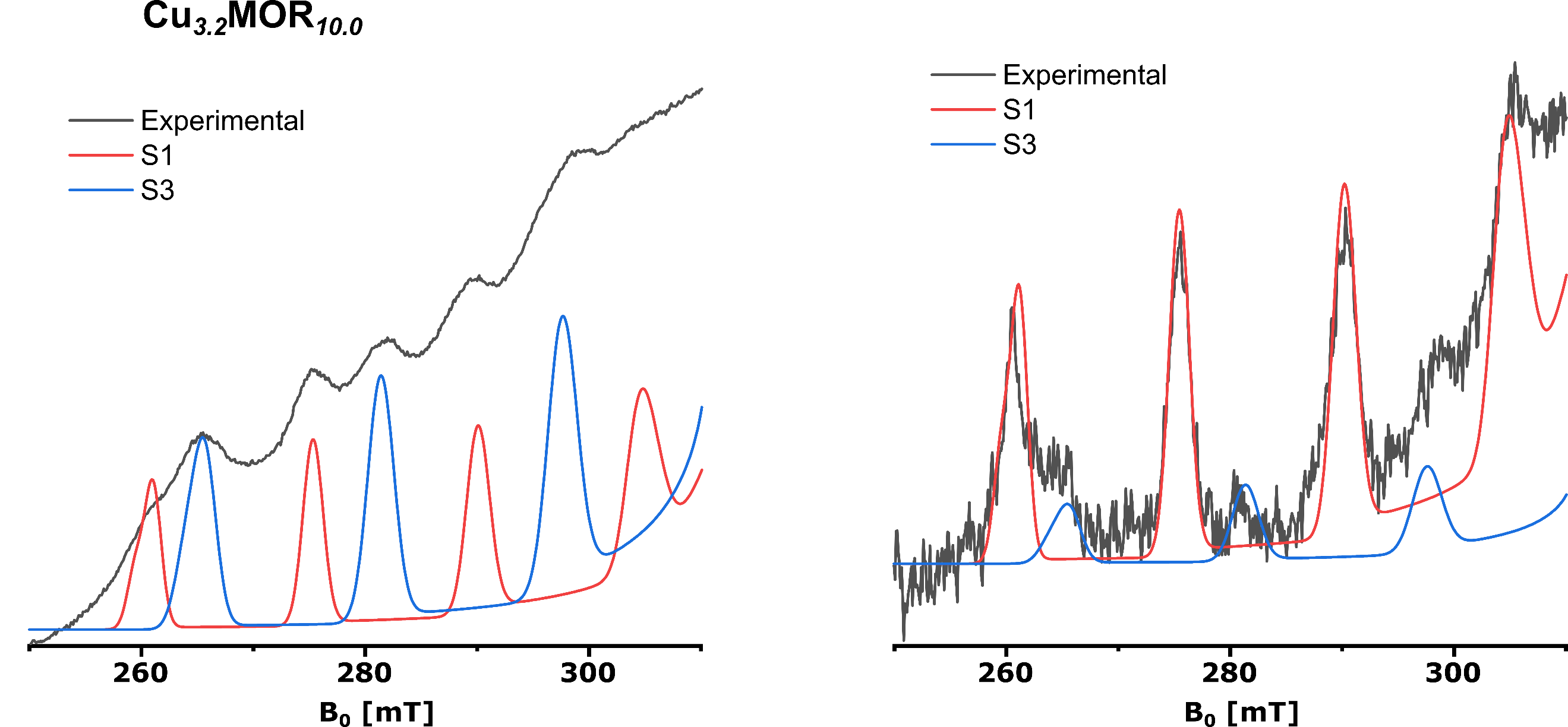
**

**Figure S6.** In situ X-band EPR spectra of Cu*_3.2_*MOR*_10.0_* recorded at room temperature after regular activation (left) and reduction (right). Note, that the line shape of the activated sample is very broad due to the dipole-dipole interaction of the Cu(II) sites. Thus, the simulation emphasizes the type of species present and not the overall line shape. The parameters for the simulations are given in Table S4.

**
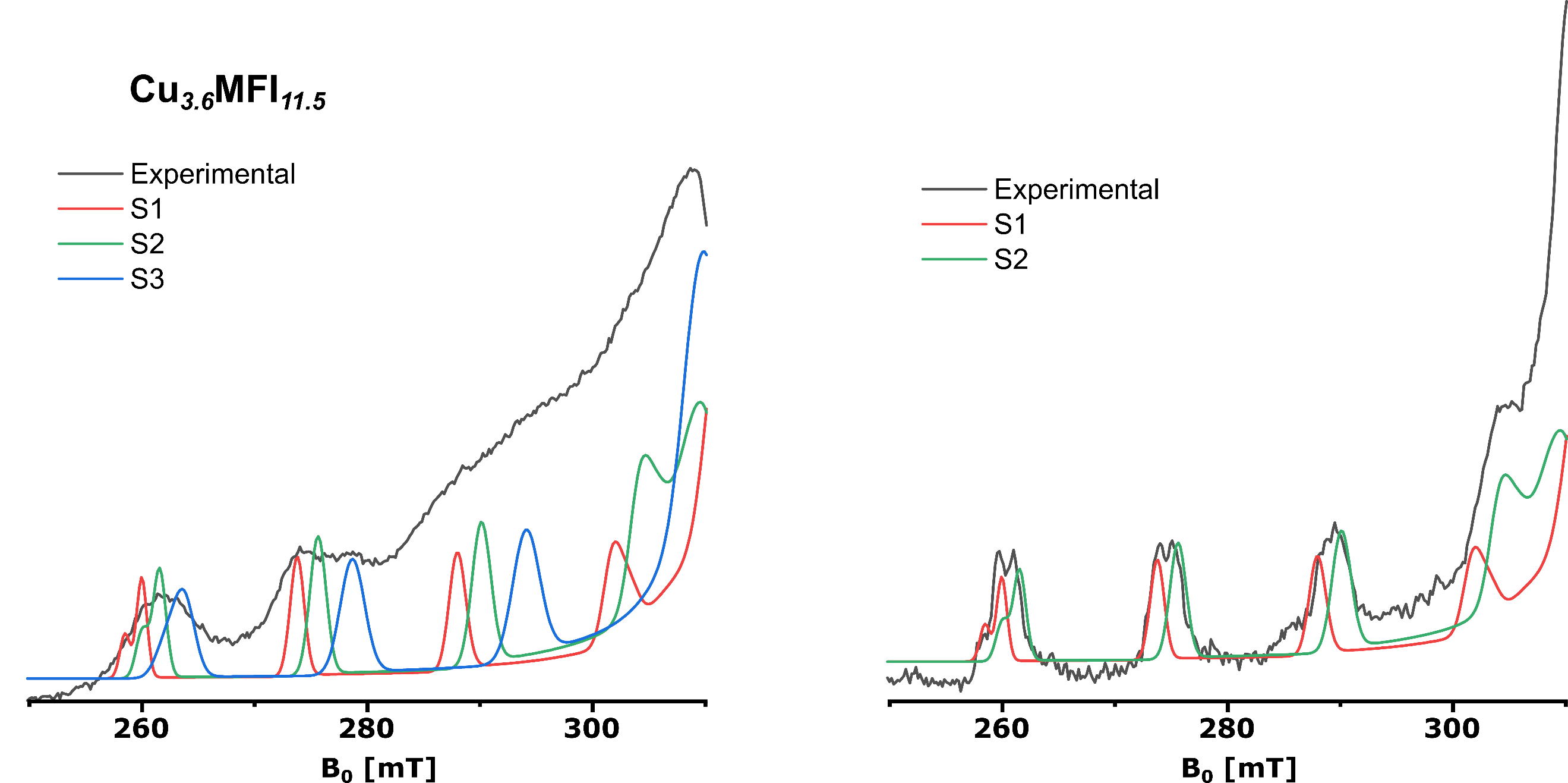
**

**Figure S7.** In situ X-band EPR spectra of Cu*_3.6_*MFI*_11.5_* recorded at room temperature after regular activation (left) and reduction (right). Note, that the line shape of the activated sample is very broad due to the dipole-dipole interaction of the Cu(II) sites. Thus, the simulation emphasizes the type of species present and not the overall line shape. The parameters for the simulations are given in Table S5.

**
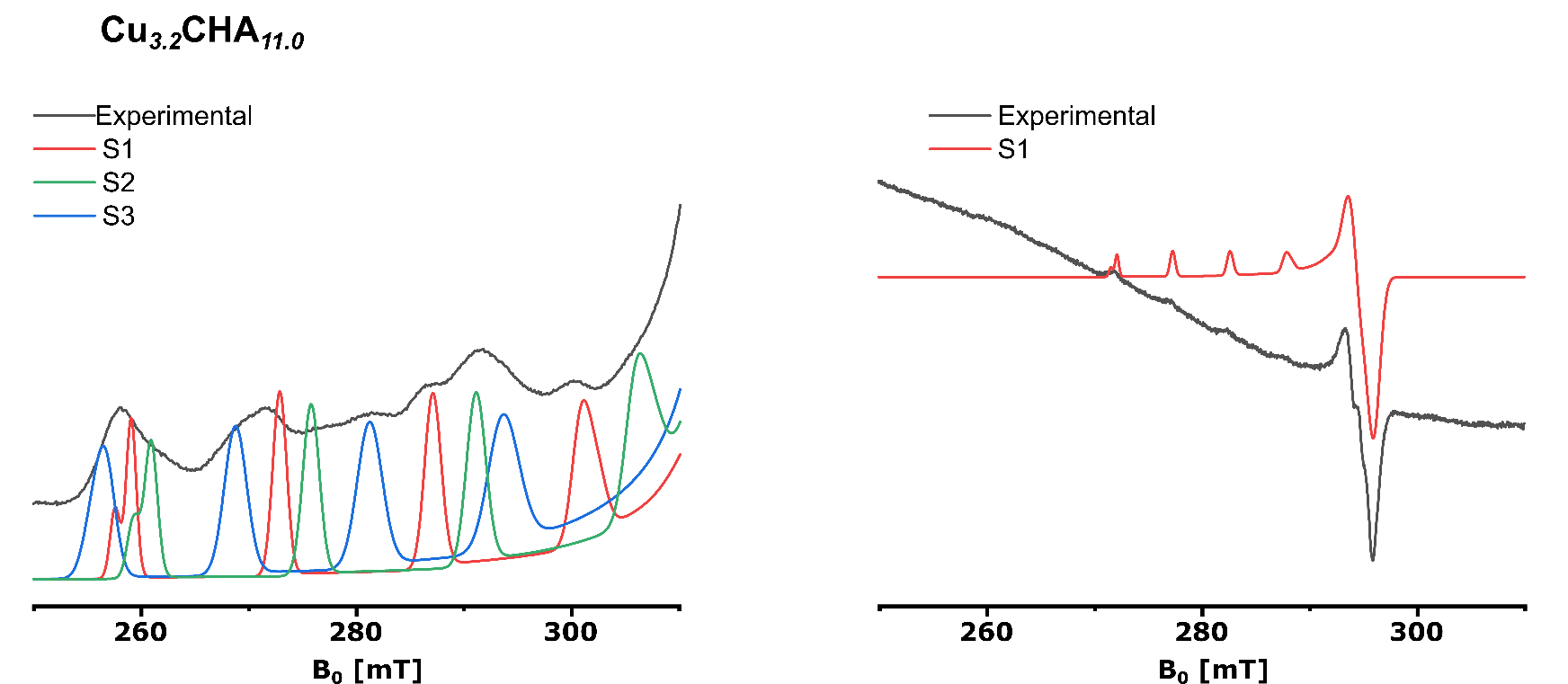
**

**Figure S8.** In situ X-band EPR spectra of Cu*_3.2_*CHA*_11.0_* recorded at room temperature after regular activation (left) and reduction (right). Note, that the line shape of the oxidized sample is very broad due to the dipole-dipole interaction of the Cu(II) sites. Thus, the simulation emphasizes the type of species present and not the overall line shape. The parameters for the simulations are given in Table S6.

**Table S4.** Parameters of the simulated spectra shown in Fig. S6.

|  | S1 | S3 |
| --- | --- | --- |
| g | g_⊥_: 2.07 g_II_: 2.33 | g_⊥_: 2.07 g_II_: 2.27 |
| A [MHz] | A_⊥_: 17 A_II_: 472 | A_⊥_: 38 A_II_: 507 |
| g-strain | g_⊥_: 0.027 g_II_: 0.0180 | g_⊥_: 0.030 g_II_: 0.020 |

**Table S5.** Parameters of the simulated spectra shown in Fig. S7.

|  | S1 | S2 | S3 |
| --- | --- | --- | --- |
| g | g_⊥_: 2.07 g_II_: 2.33 | g_⊥_: 2.07 g_II_: 2.31 | g_⊥_: 2.07 g_II_: 2.28 |
| A [MHz] | A_⊥_: 17 A_II_: 492 | A_⊥_: 10 A_II_: 498 | A_⊥_: 38 A_II_: 522 |
| g-strain | g_⊥_: 0.027 g_II_: 0.0130 | g_⊥_: 0.04 g_II_: 0.015 | g_⊥_: 0.030 g_II_: 0.020 |

**Table S6.** Parameters of the simulated spectra shown in Fig. S8.

|  | S1 | S2 | S3 |
| --- | --- | --- | --- |
| g | g_⊥_: 2.07 g_II_: 2.36 | g_⊥_: 2.07 g_II_: 2.33 | g_⊥_: 2.07 g_II_: 2.4 |
| A [MHz] | A_⊥_: 17 A_II_: 460 | A_⊥_: 10 A_II_: 490 | A_⊥_: 36 3 A_II_: 410 |
| g-strain | g_⊥_: 0.027 g_II_: 0.0130 | g_⊥_: 0.04 g_II_: 0.015 | g_⊥_: 0.030 g_II_: 0.020 |

**
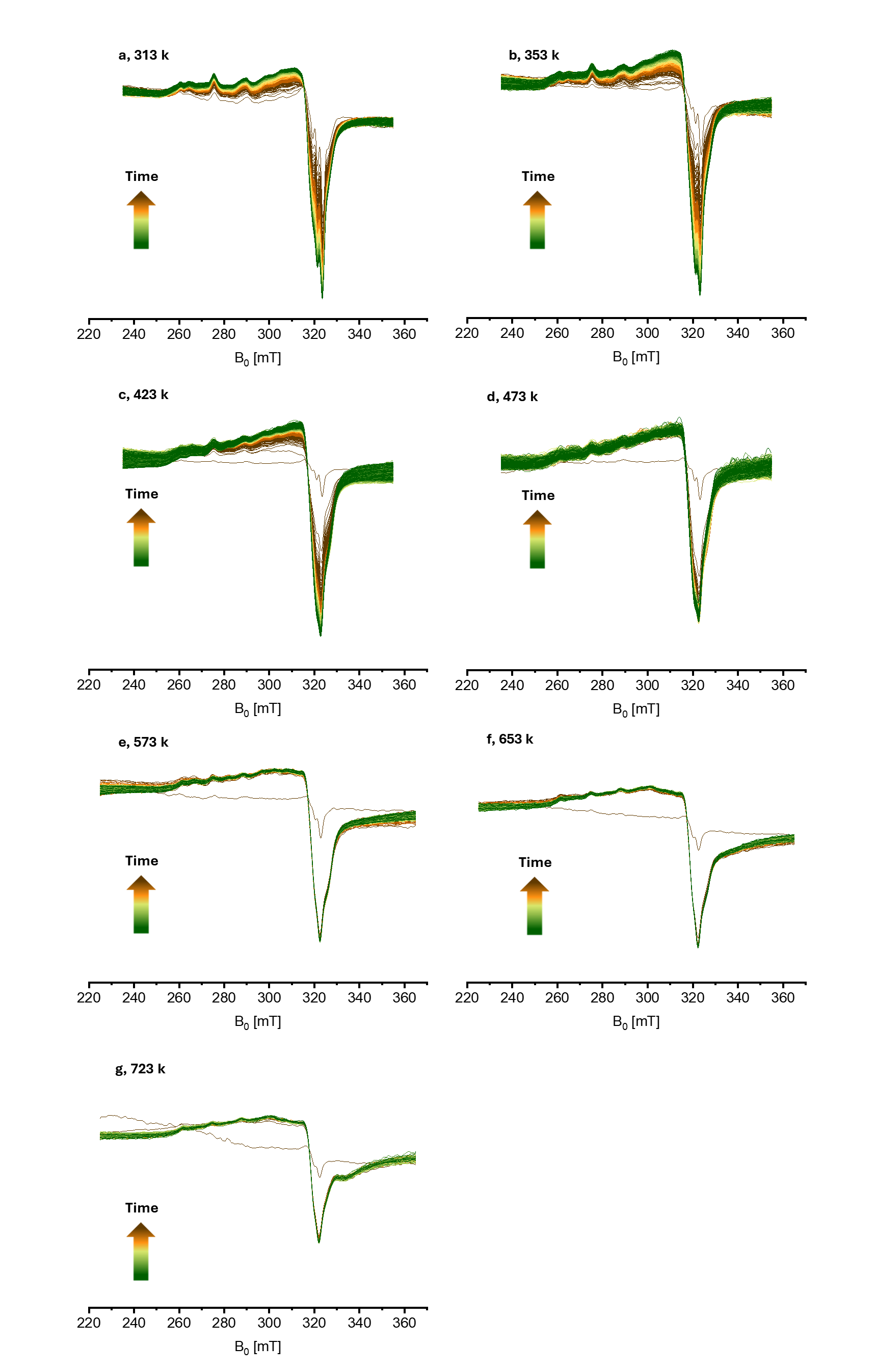
**

**Figure S9.** Operando X-band EPR spectra of Cu*_3.2_*MOR*_10.0_* during isothermal re-activation at 313 K for 11 h (a), 353 K for 11 h (b), 423 K for 11 h (c), 473 K for
11 h (d), 573 K for 1.5 h (e), 653 K for 1.5 h (f), and 723 K for 1.5 h (g) with ~10 mbar O_2_.


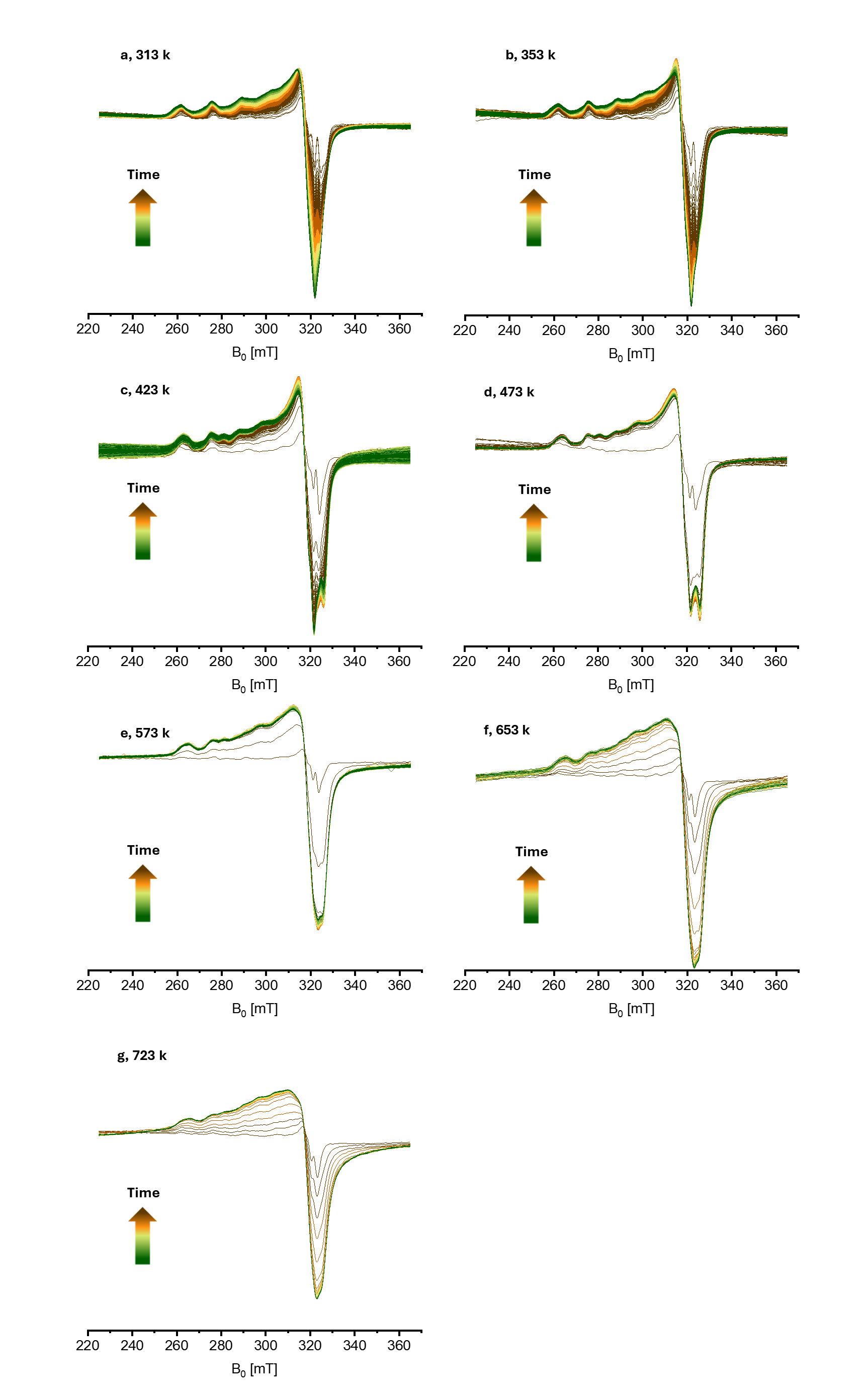


**Figure S10.** Operando X-band EPR spectra of Cu*_3.6_*MFI*_11.5_* during isothermal re-activation at 313 K for 16 h (a), 353 K for 16 h (b), 423 K for 16 h (c), 473 K for
16 h (d), 573 K for 1 h (e), 653 K for 1 h (f), and 723 K for 1 h (g) with ~10 mbar O_2_.

**
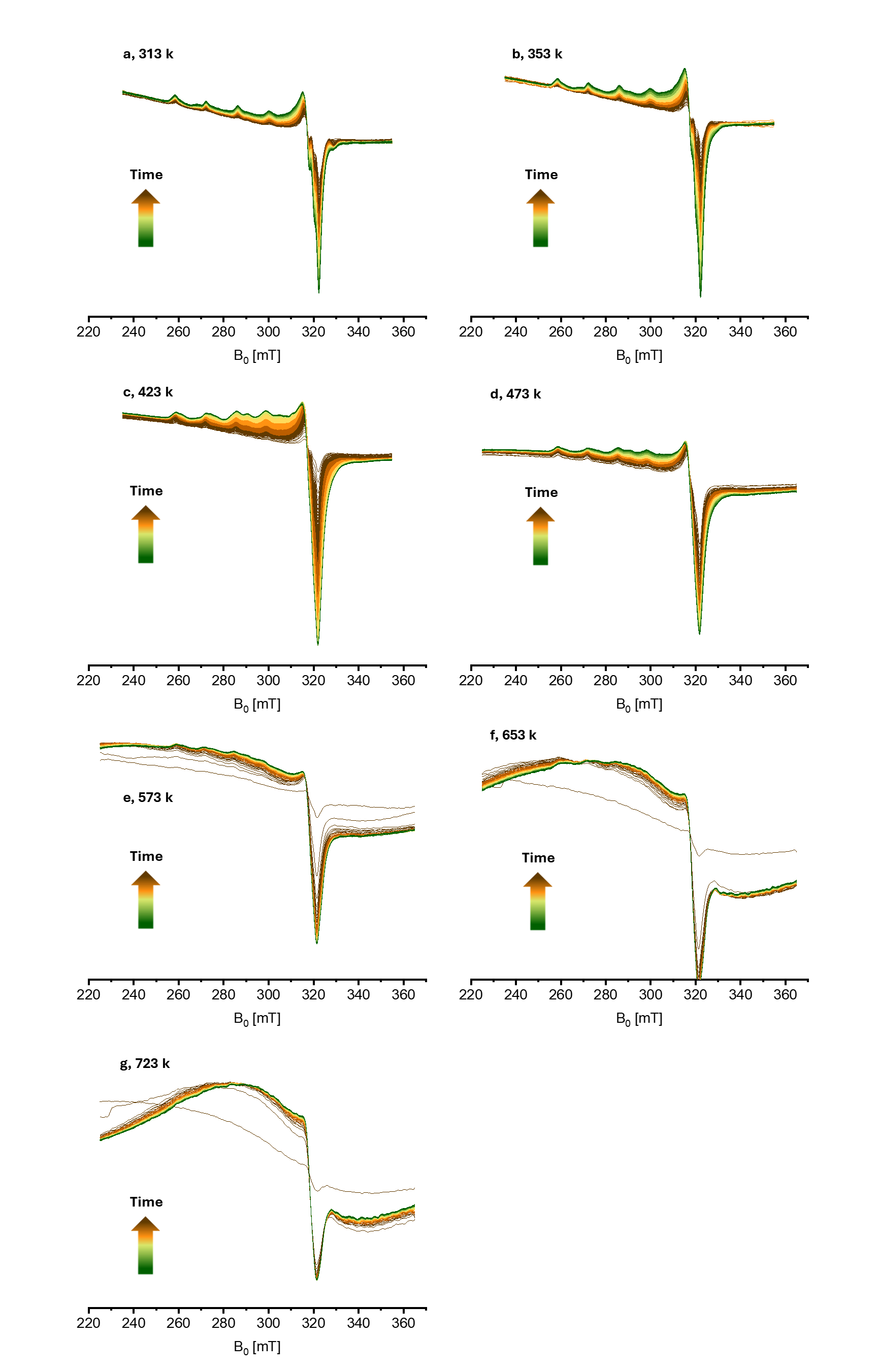
**

**Figure S11.** Operando X-band EPR spectra of Cu*_3.2_*CHA*_11.0_* during isothermal re-activation at 313 K for 5.5 h (a), 353 K for 5.5 h (b), 423 K for 4 h (c), 473 K for 5.5 h (d), 573 K for 1 h (e), 653 K for 1 h (f), and 723 K for 1 h (g) with ~10 mbar O_2_.


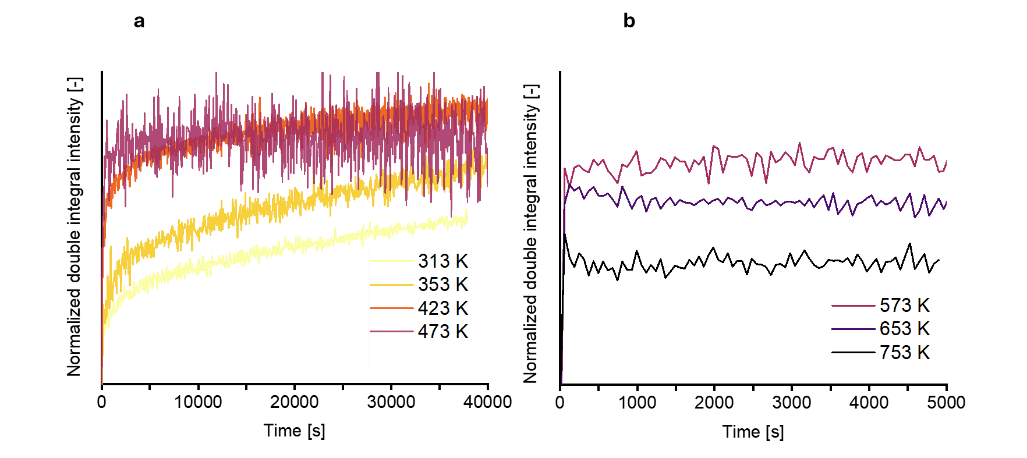


**Figure S12.** Development of the double integral intensity of Cu*_3.2_*MOR*_10.0_* at temperatures in the range between 313 and 473 K (a) and in the range between 573 and 723 K (b) with ~10 mbar O_2_.


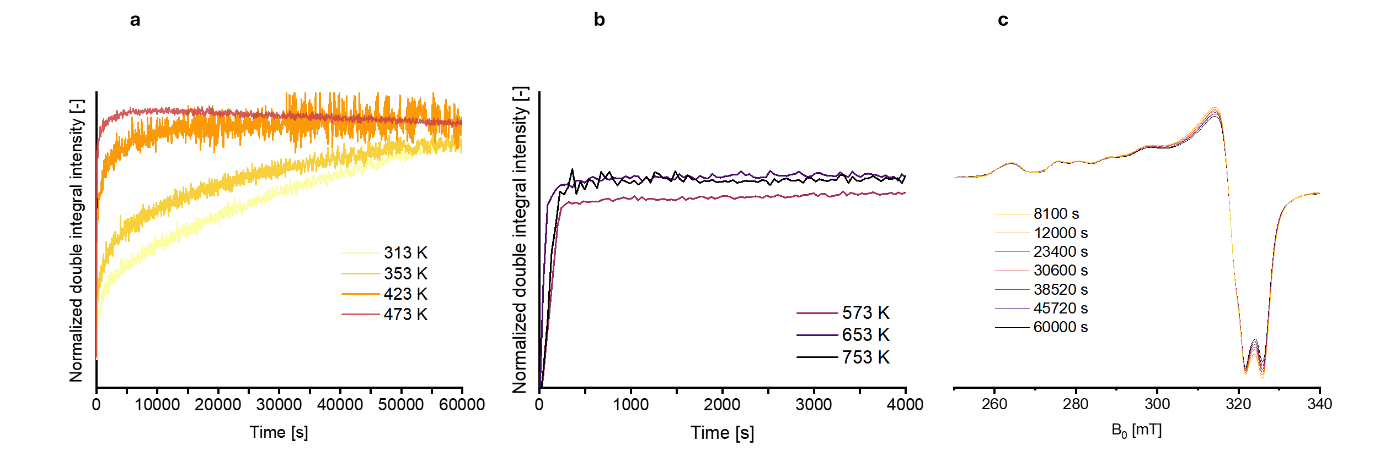


**Figure S13.** Development of the double integral intensity of Cu*_3.6_*MFI*_11.5_* at temperatures in the range between 313 and 473 K (a) and in the range between 573 and 723 K (b) with ~10 mbar O_2_.Examplary spectra of Cu*_3.6_*MFI*_11.5_* at 473 K at different points in time, highlighting the marginal autoreduction (c).


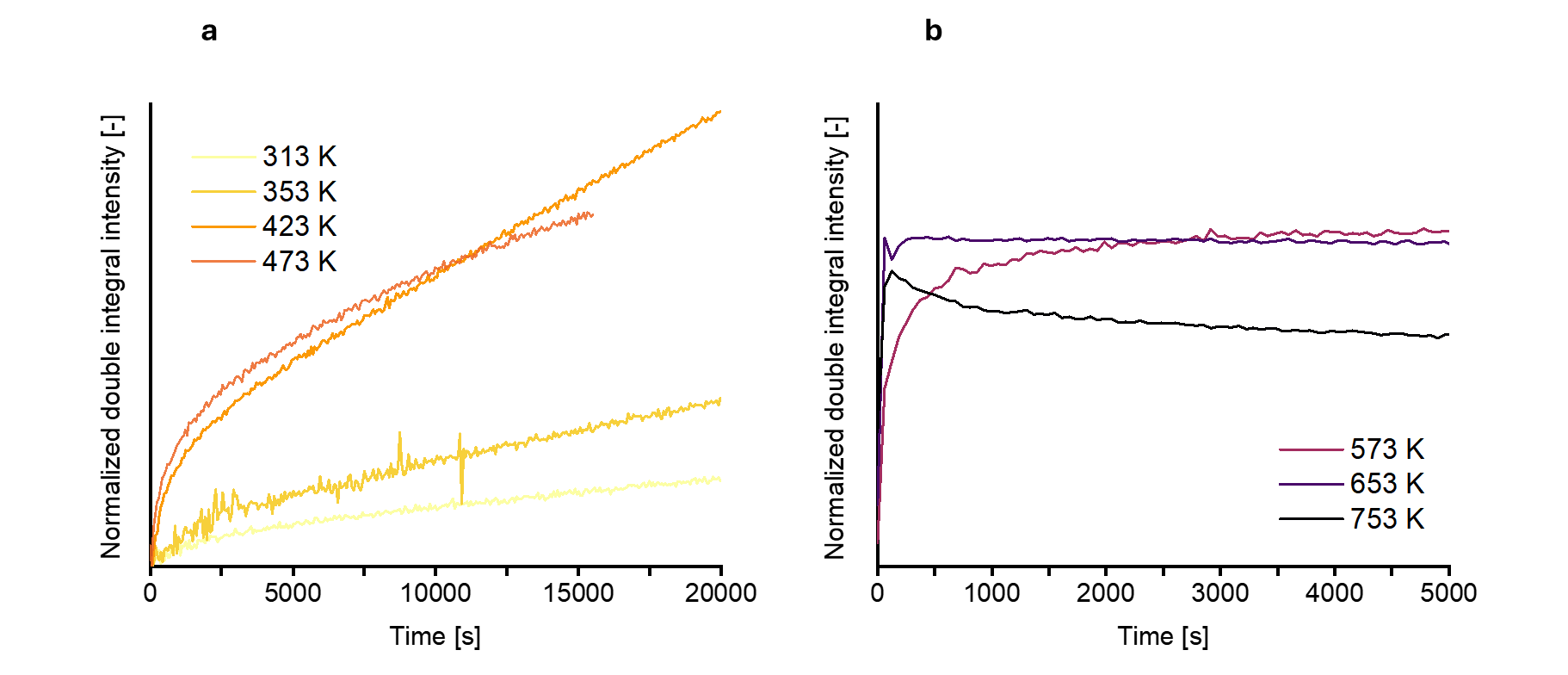


**Figure S14.** Development of the double integral intensity of Cu*_3.2_*CHA*_11.0_* at temperatures in the range between 313 and 473 K (a) and in the range between 573 and 723 K (b) with ~10 mbar O_2_.





**Figure S15.** In situ FTIR spectra of the O-H stretching frequency region of Cu*_3.2_*MOR*_10.0_* (a) Cu*_3.6_*MFI*_11.5_* (b) and Cu*_3.2_*CHA*_11.0_* (c) after regular activation (vibrant colors) and after reduction at 753 K in CH_4_ (pale colors). The band around 3745 cm^-1^ corresponds to terminal silanol groups, whereas the band at ~3610 cm^-1^ corresponds to Brønsted acid sites (BAS).^[15]^


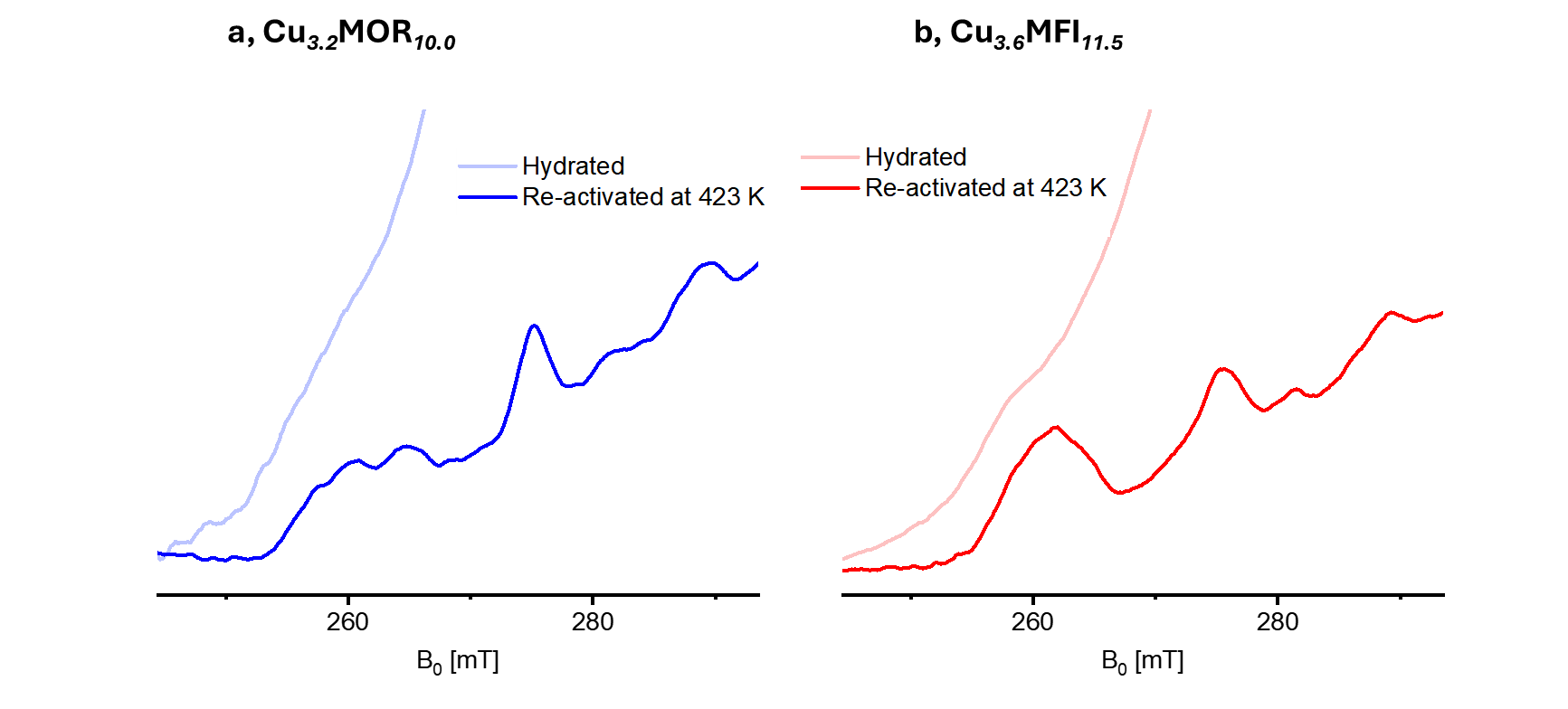


**Figure S16.** In situ X-band EPR spectra of hydrated Cu*_3.2_*MOR*_10.0_* (a) and Cu*_3.6_*MFI*_11.5_* (b) as well as the corresponding spectra after isothermal re-activation at
423 K.


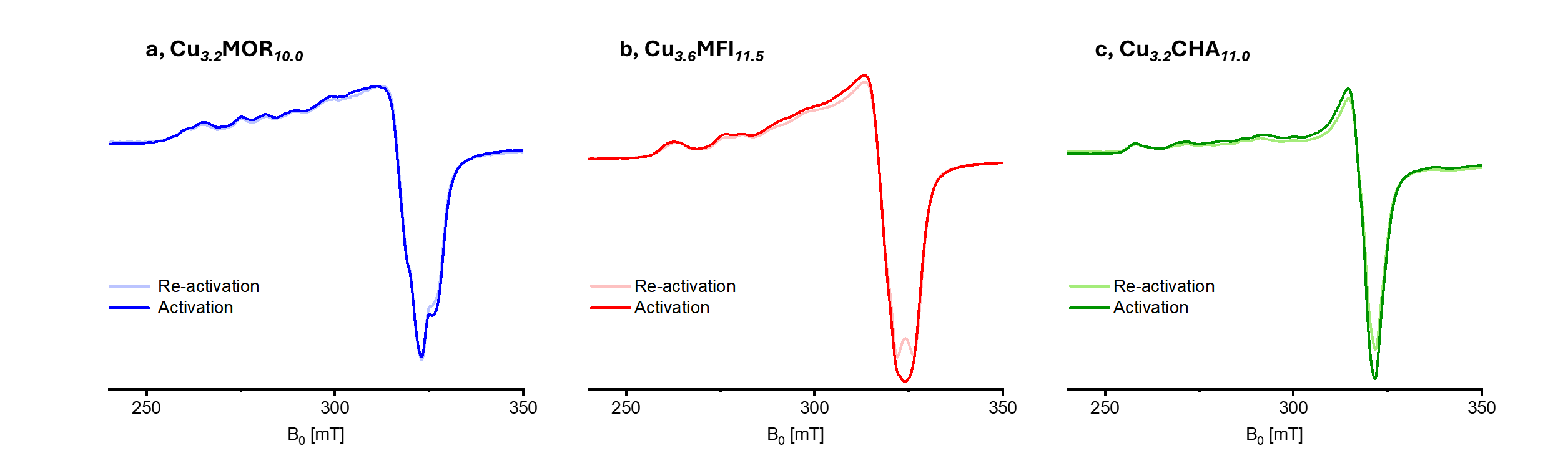


**Figure S17.** In situ X-band EPR spectra of Cu*_3.2_*MOR*_10.0_* (a) Cu*_3.6_*MFI*_11.5_* (b) and Cu*_3.2_*CHA*_11.0_* (c) after re-activation at 723 K with ~10 mbar O_2_ and regular activation at 753 K.


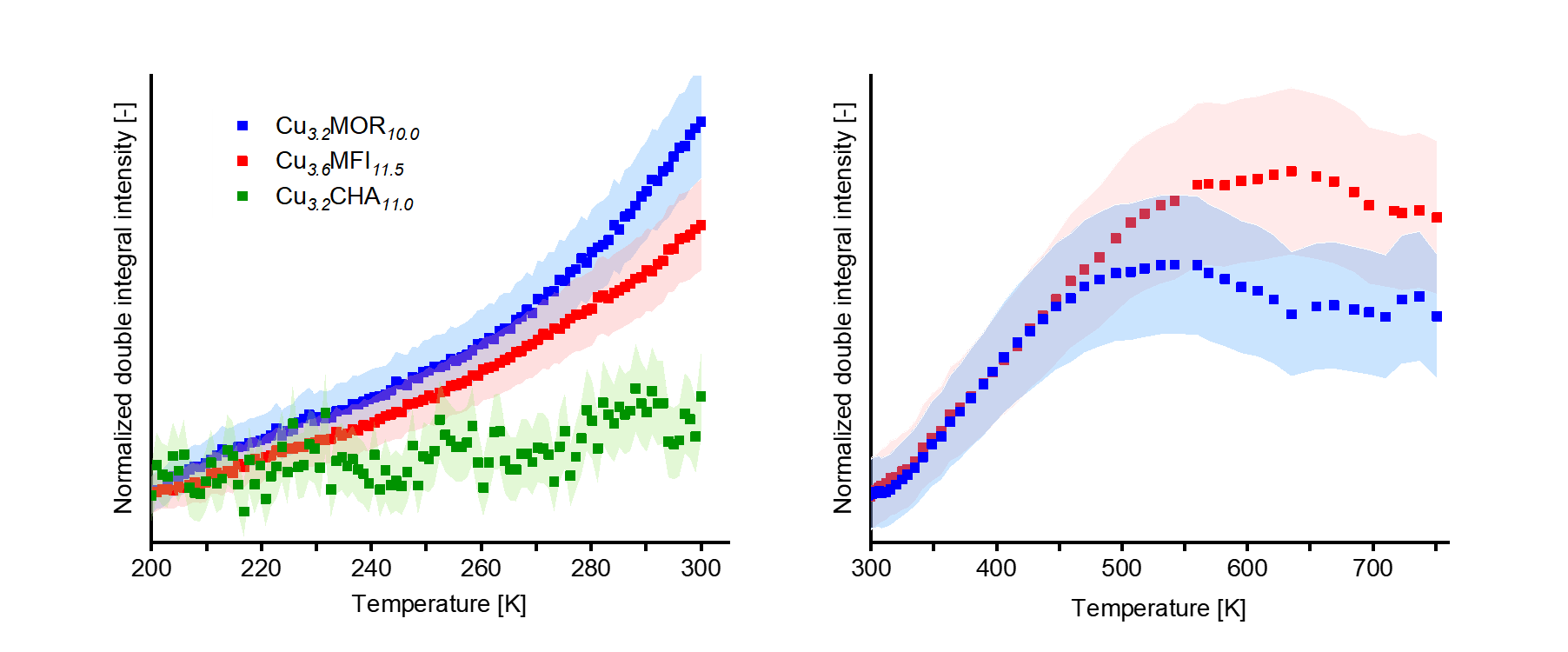


**Figure S18.** Development of monomeric Cu(II) in Cu*_3.2_*MOR*_10.0_*, Cu*_3.6_*MFI*_11.5_*, and Cu*_3.2_*CHA*_11.0_* as a function of temperature in the range between 200 and 300 K (left) and in Cu*_3.2_*MOR*_10.0_* and Cu*_3.6_*MFI*_11.5_* in the range between 300 and 753 K. Multiple factors, including uncertainty in temperature and variation in the Q value of the resonator, contribute to the measurement uncertainty, which was assumed to be 15%.

**
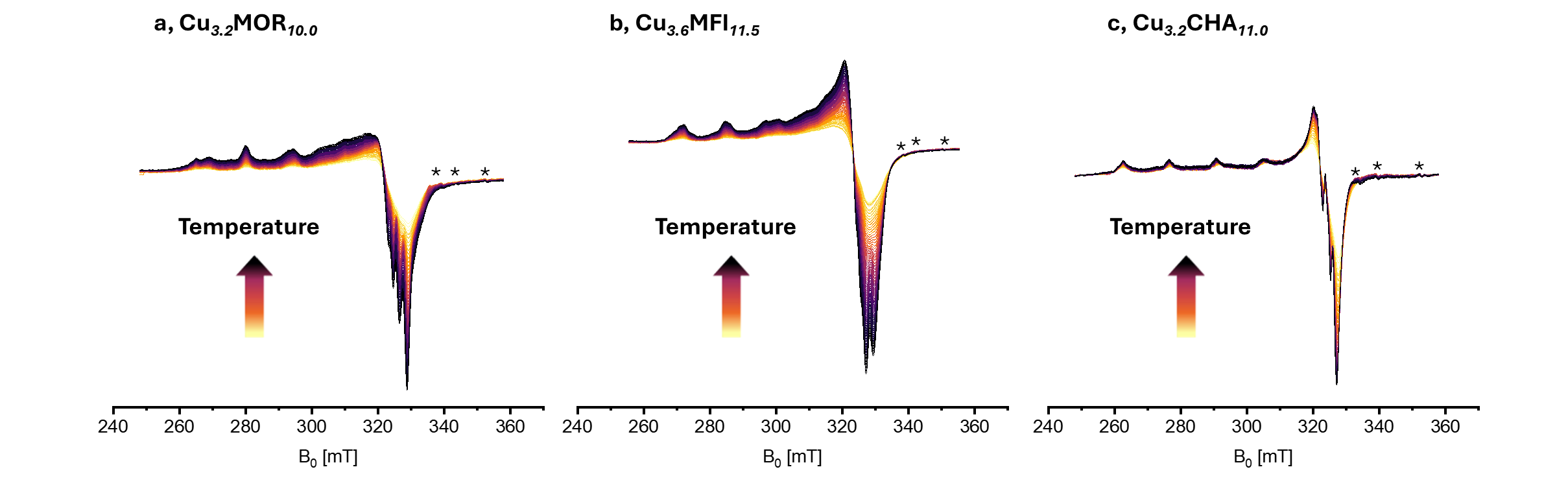
**

**Figure S19.** In situ EPR spectra of Cu*_3.2_*MOR*_10.0_* (a) Cu*_3.6_*MFI*_11.5_* (b), and Cu*_3.2_*CHA*_11.0_* (c) in the temperature range between 200 and 300 K with ~10 mbar O_2_. The asterisks indicate the signal originating from O_2_.


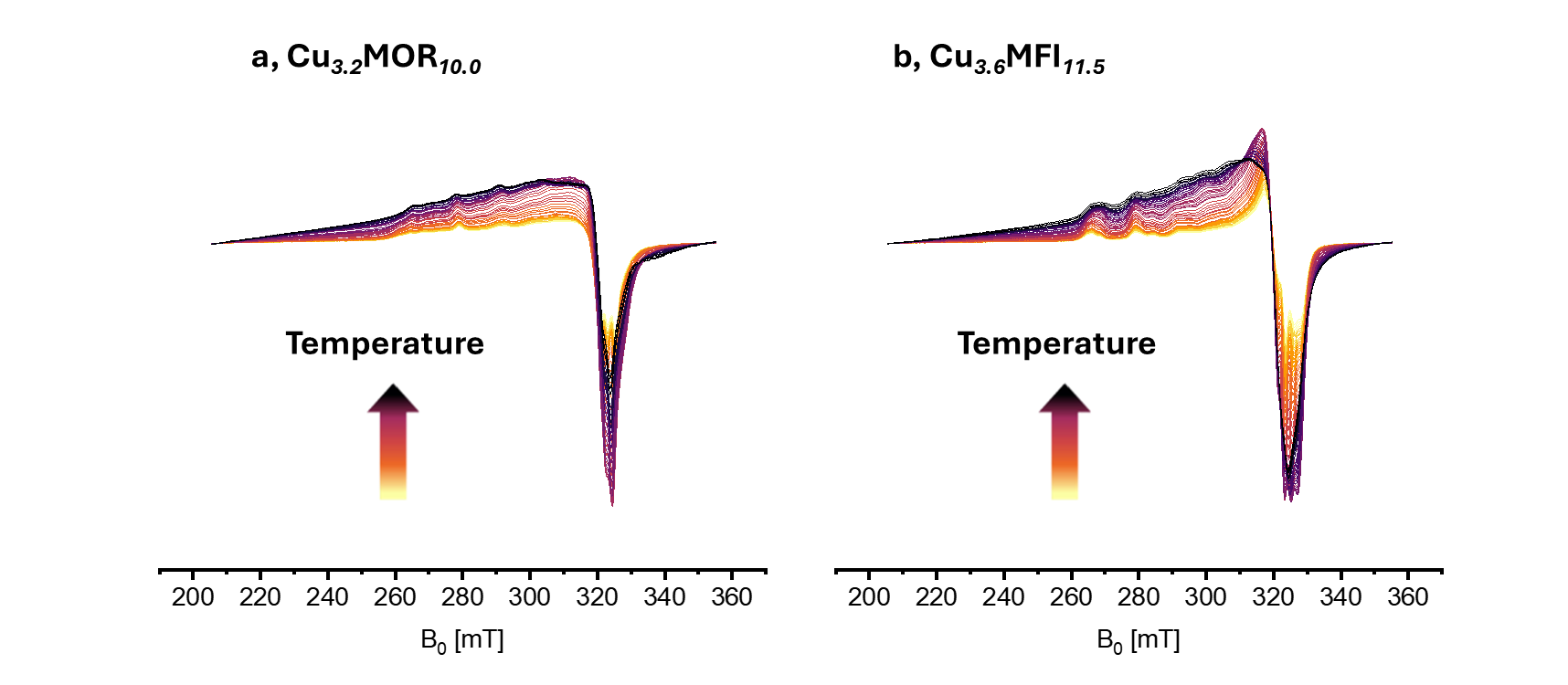


**Figure S20.** In situ EPR spectra of Cu*_3.2_*MOR*_10.0_* (a) and Cu*_3.6_*MFI*_11.5_* (b) in the temperature range between 300 and 753 K with ~10 mbar O_2_.

**
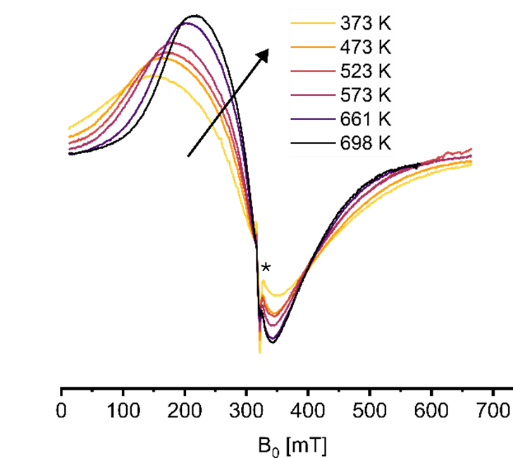
**

**Figure S21.** In situ EPR spectra of Cu*_3.2_*CHA*_11.0_* after reduction with CH_4_ at 753 K recorded in vacuum at different temperatures. The temperature-dependence of the spectral line shape is indicative for the presence of small superparamagnetic nanoparticles^[16–19]^. The asterisk indicates the remaining fraction of monomeric Cu(II).

**2.5 Operando/In Situ UV-Vis Spectroscopy**





**Figure S22.** In situ UV-Vis spectra of Cu_3.2_MOR_10.0_ (a), Cu_3.6_MFI_11.5_ (b), and Cu_3.2_CHA_11.0_ (c) after the regular activation (dotted lines, recorded in O_2_) and isothermal re-activation (solid lines, recorded in O_2_) at temperatures in the range from 313 to 723 K. Spectra of the activated and re-activated materials were measured at the same specific temperature at which the corresponding re-activation took place. The characteristic bands of S4 and S5 are highlighted in purple and orange.


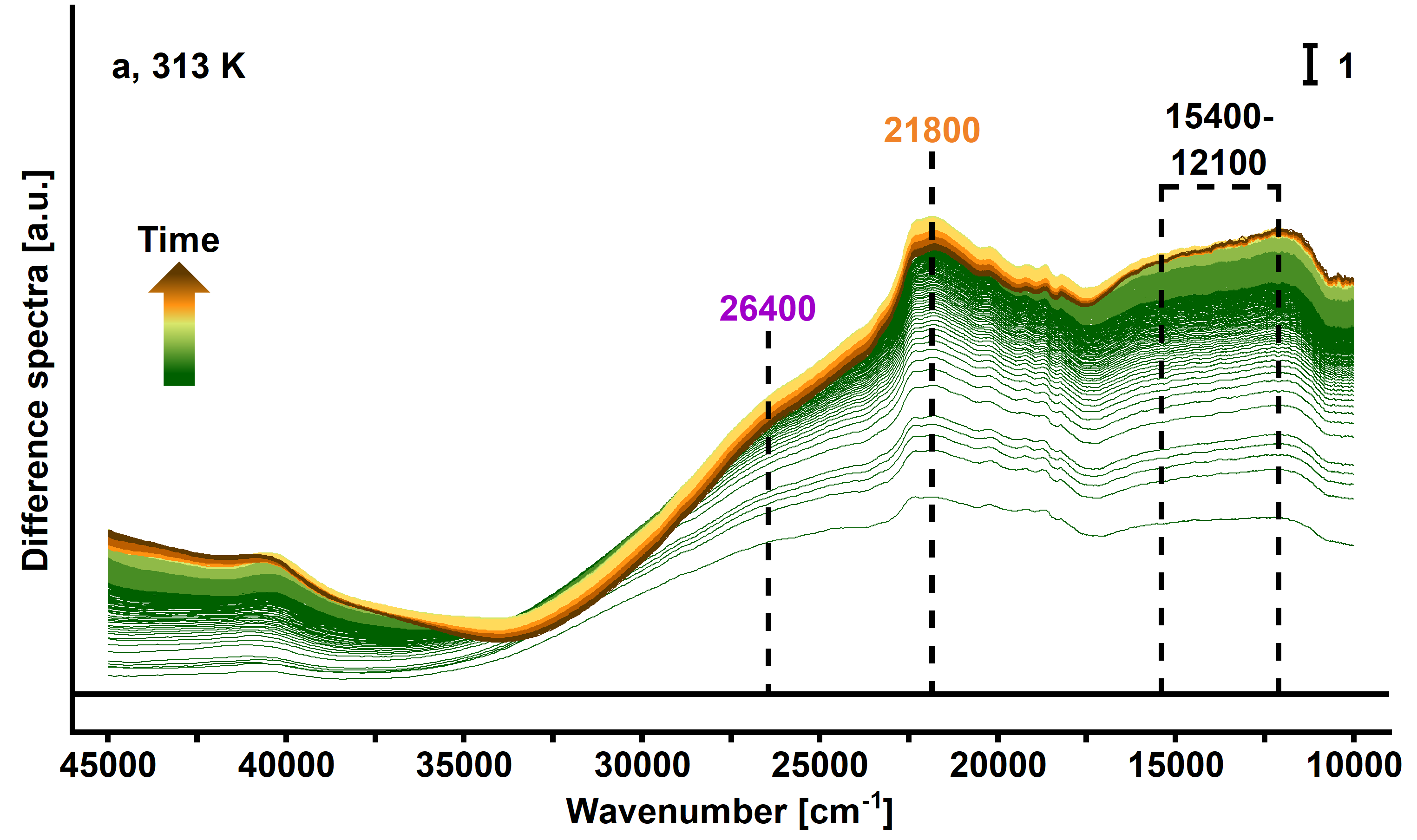

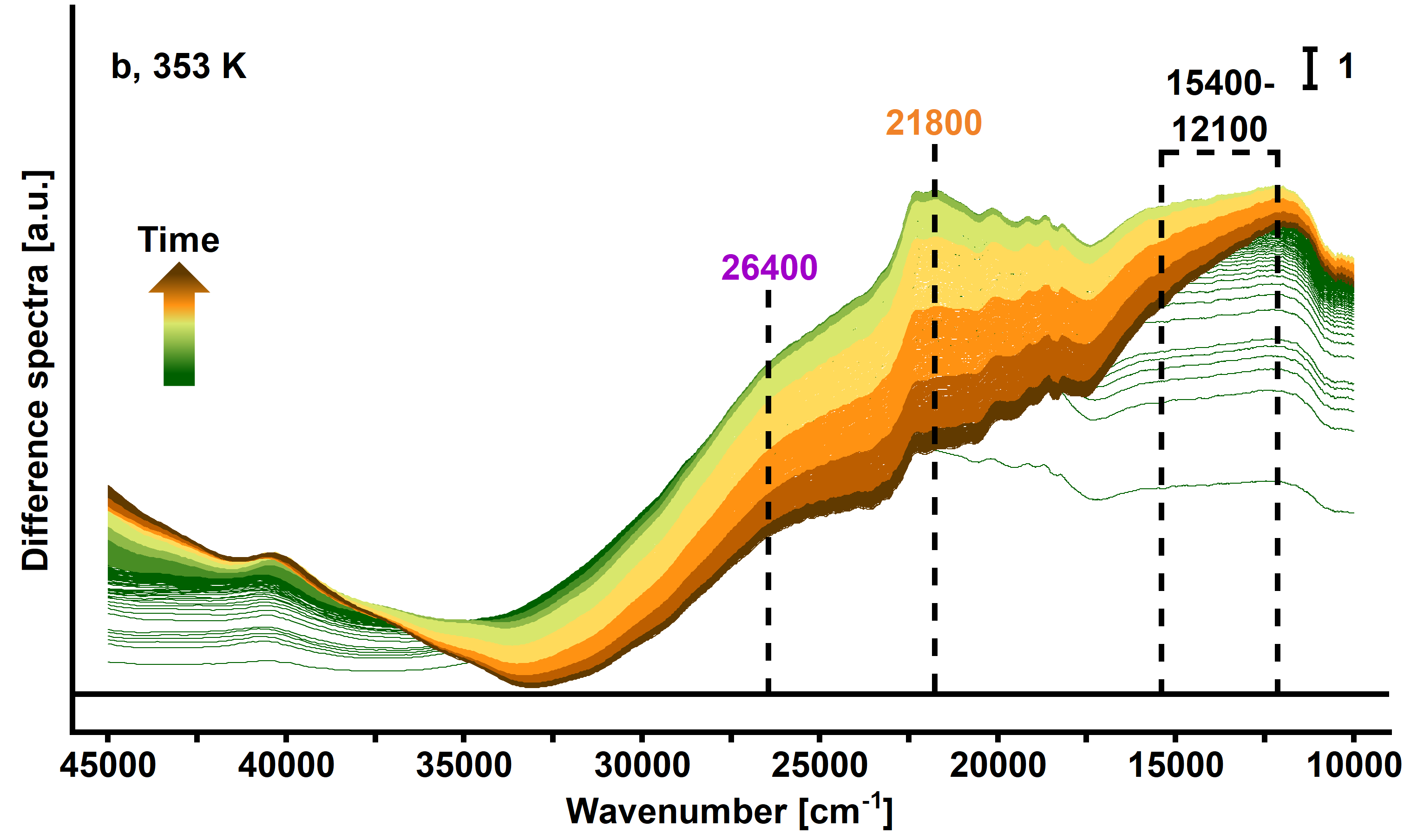

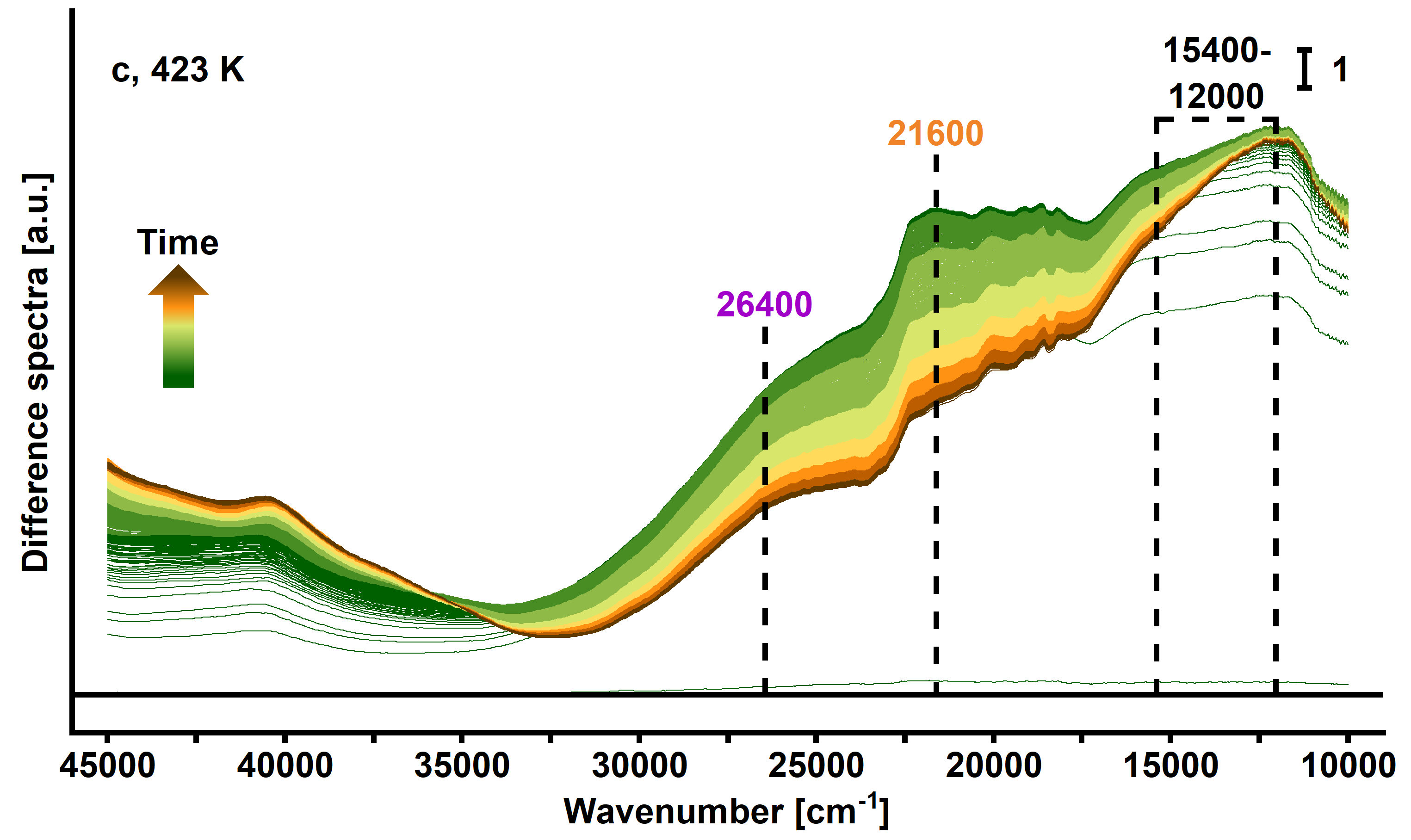

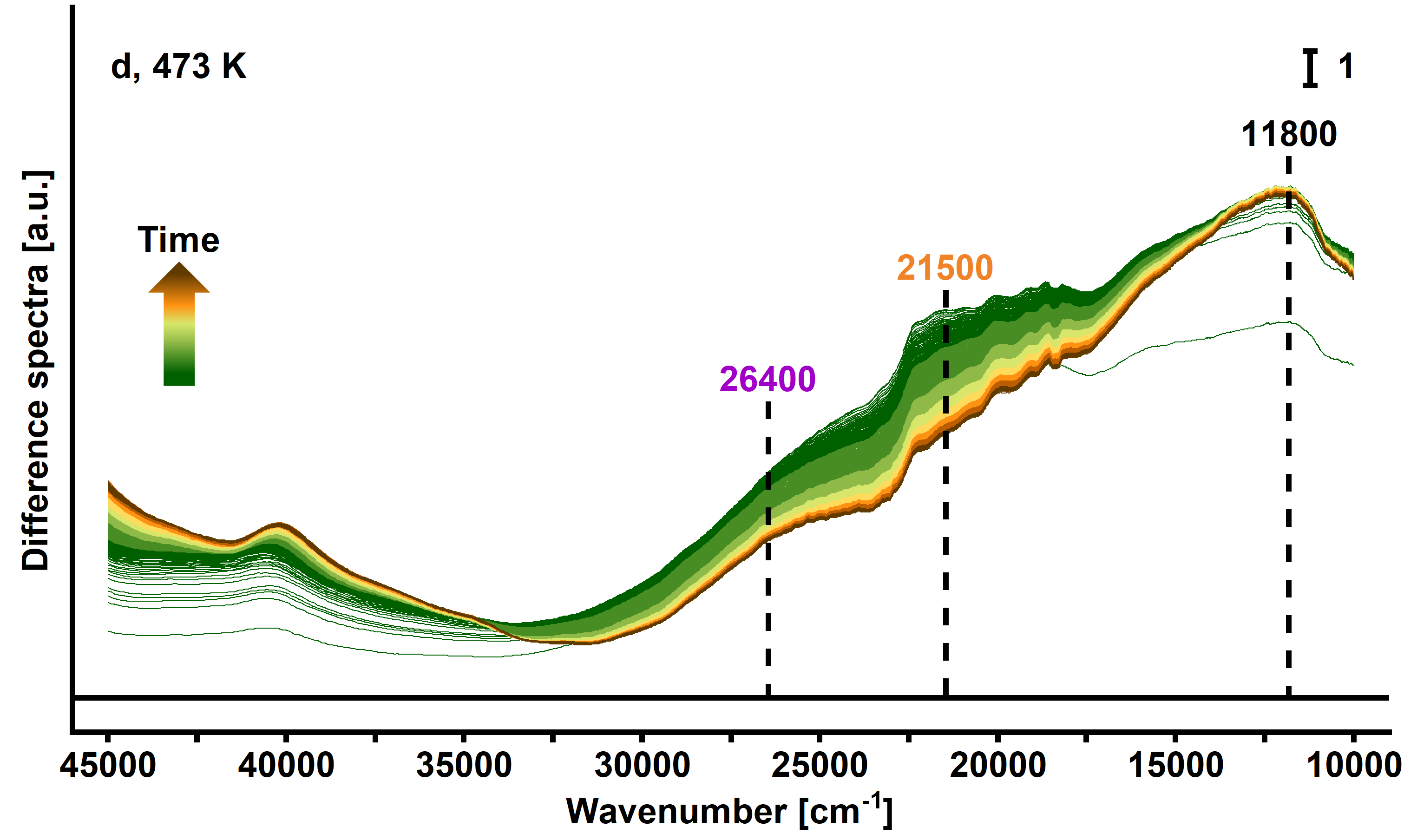

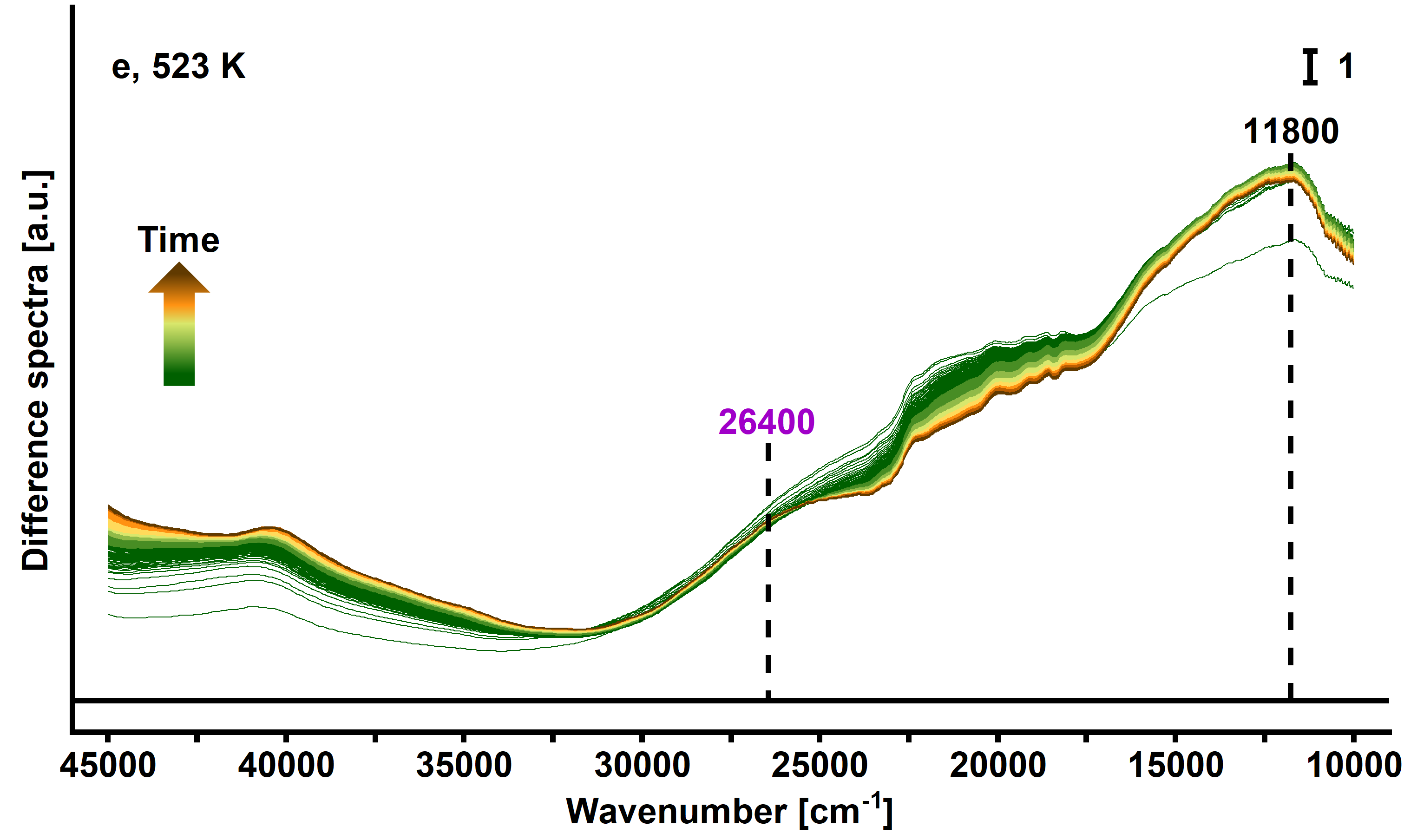

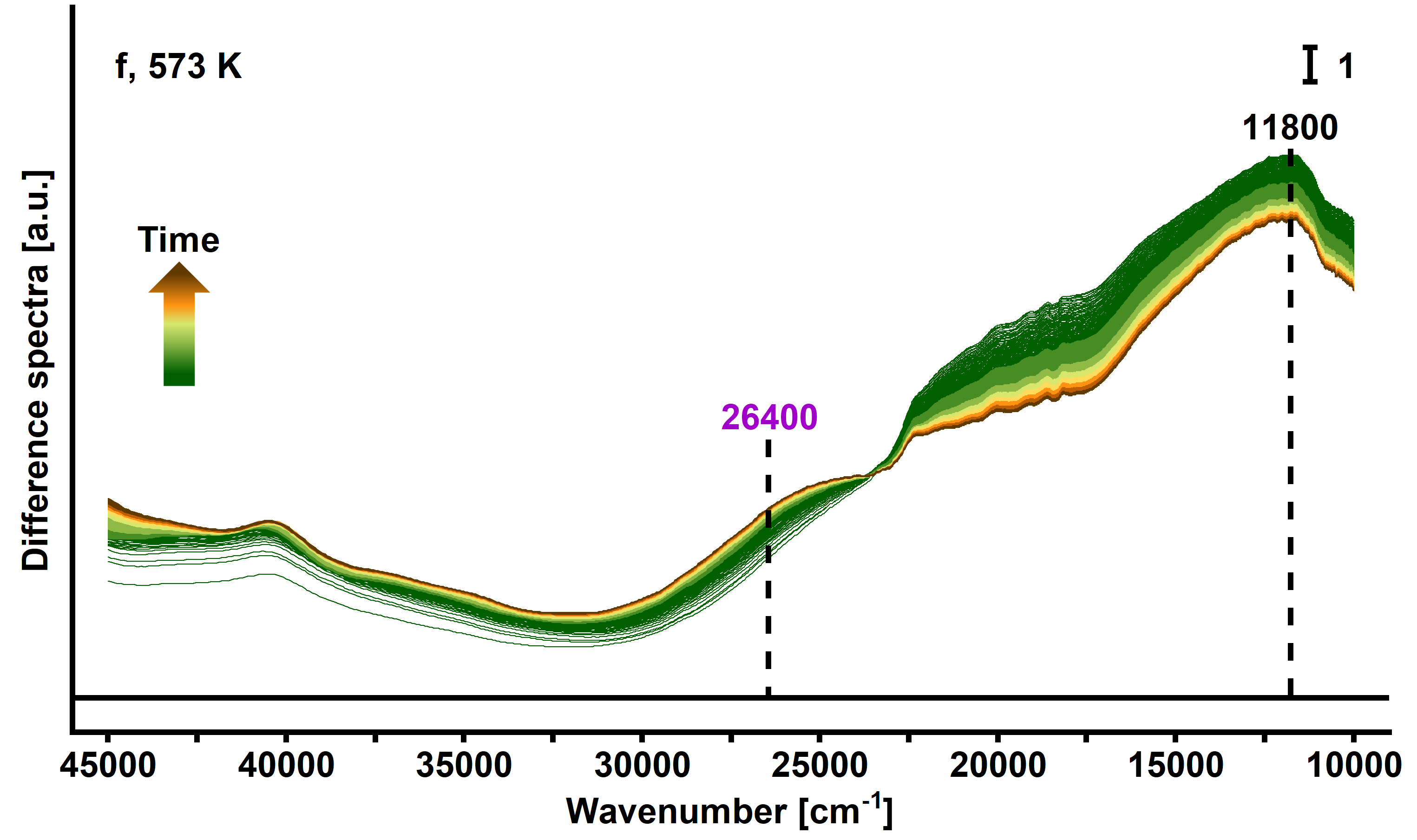

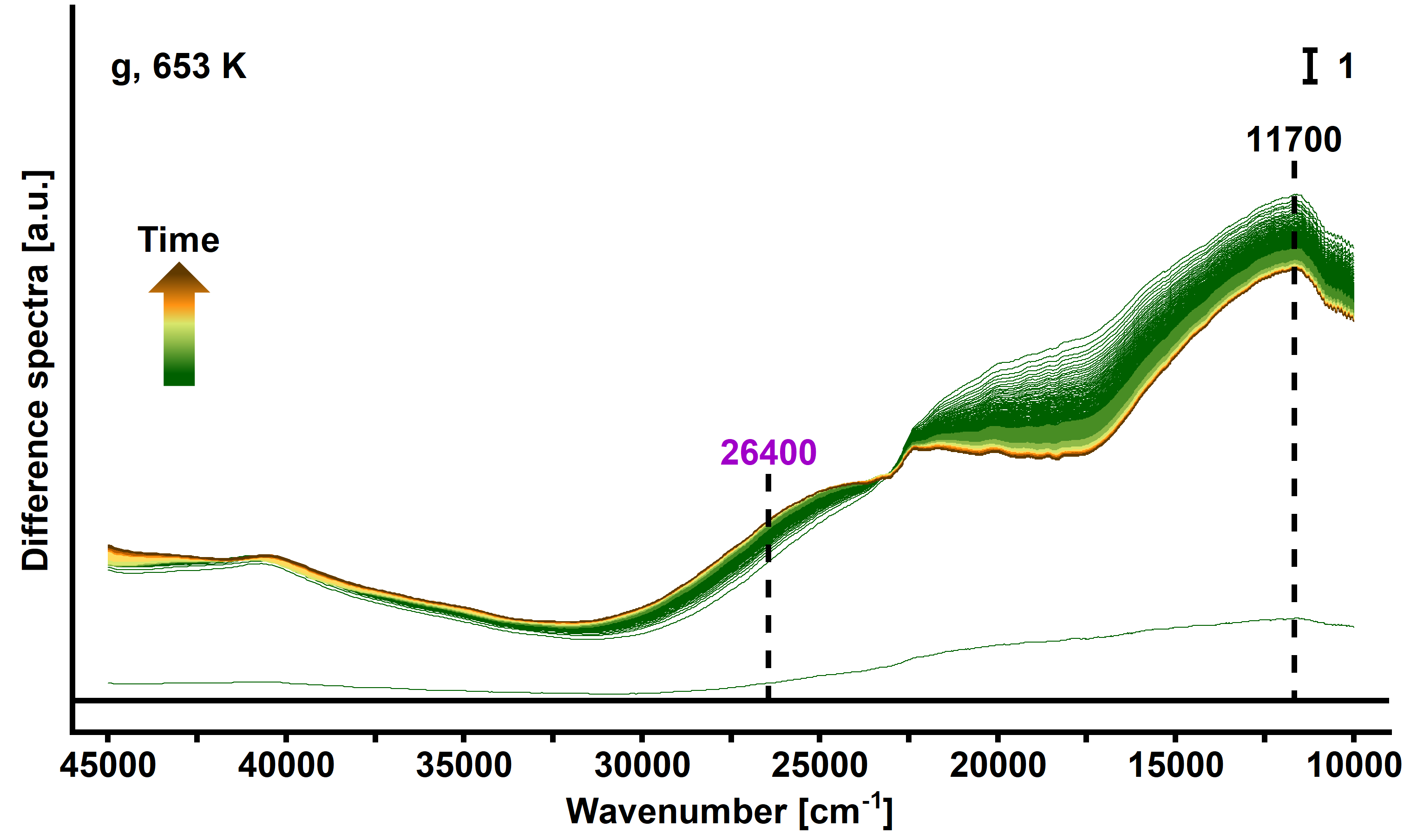

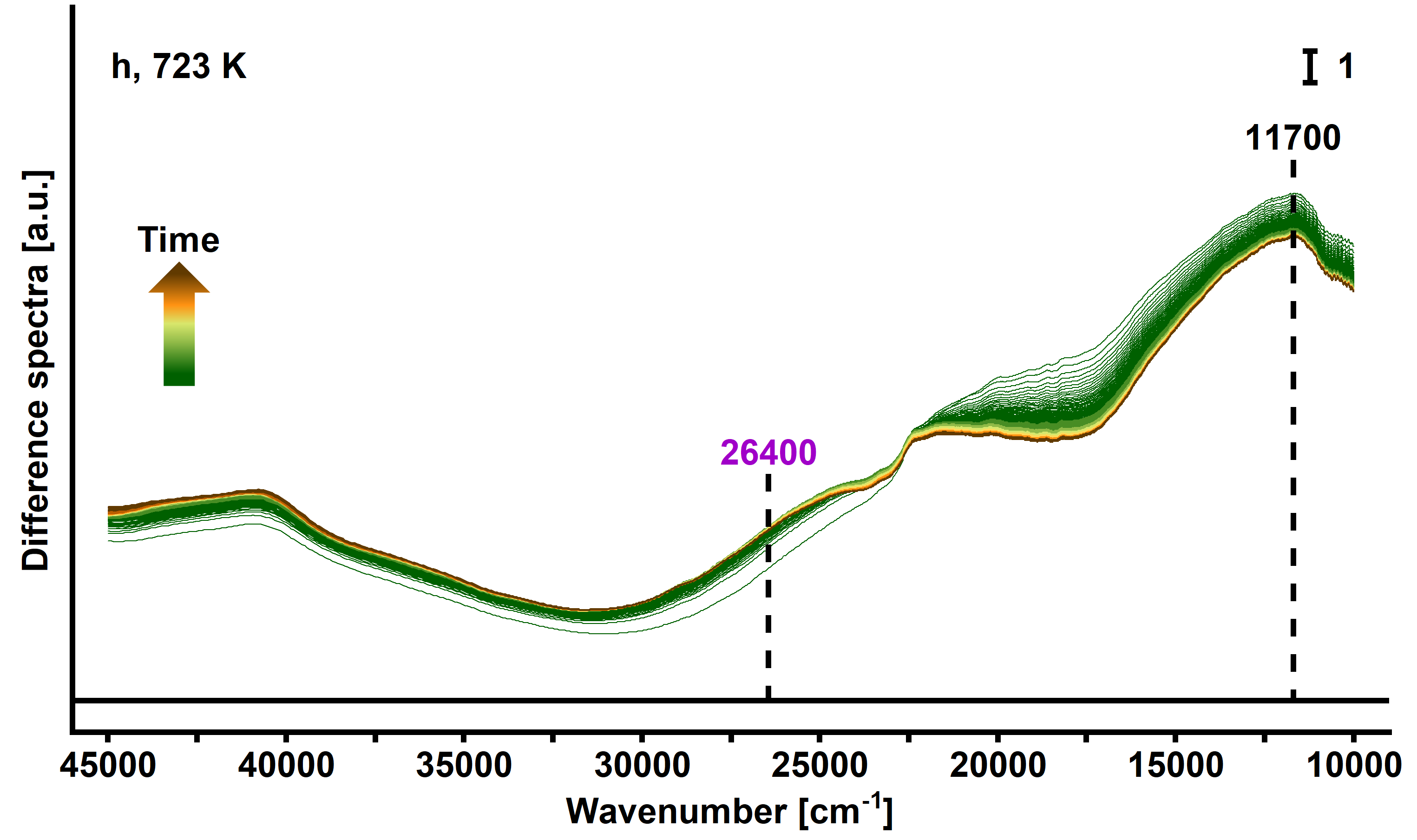


**Figure S23.** Operando UV-Vis difference spectra of Cu*_3.2_*MOR*_10.0_* during isothermal re-activation at 313 K for 19 h(a), 353 K for 16 h (b), 423 K for 16 h (c), 473 K for 18 h (d), 523 K for 17 h (e), 573 K for 16 h (f), 653 K for 17 h (g), and 723 K for 2 h (h) with O_2_. The characteristic bands of S4 and S5 are highlighted in purple and orange.


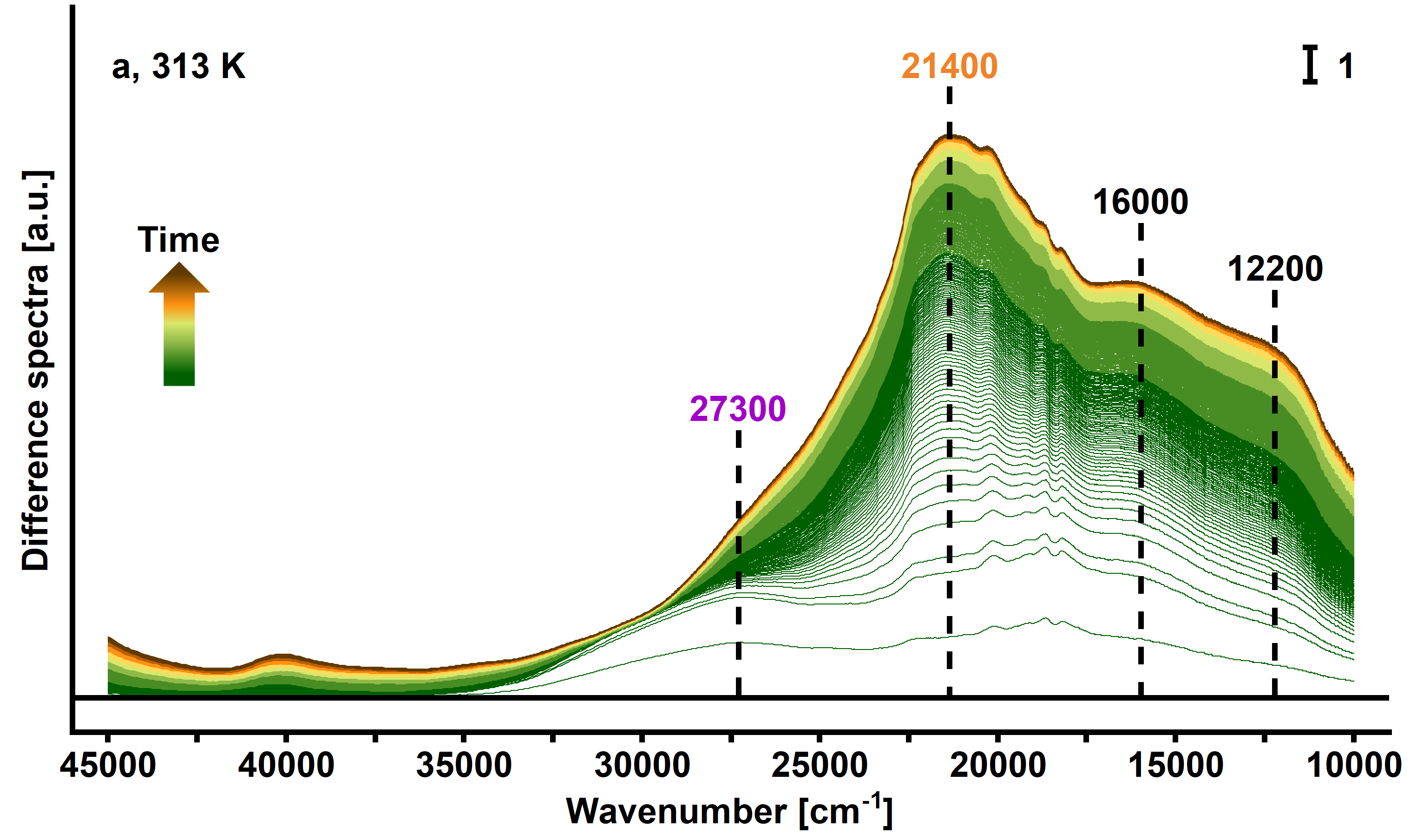

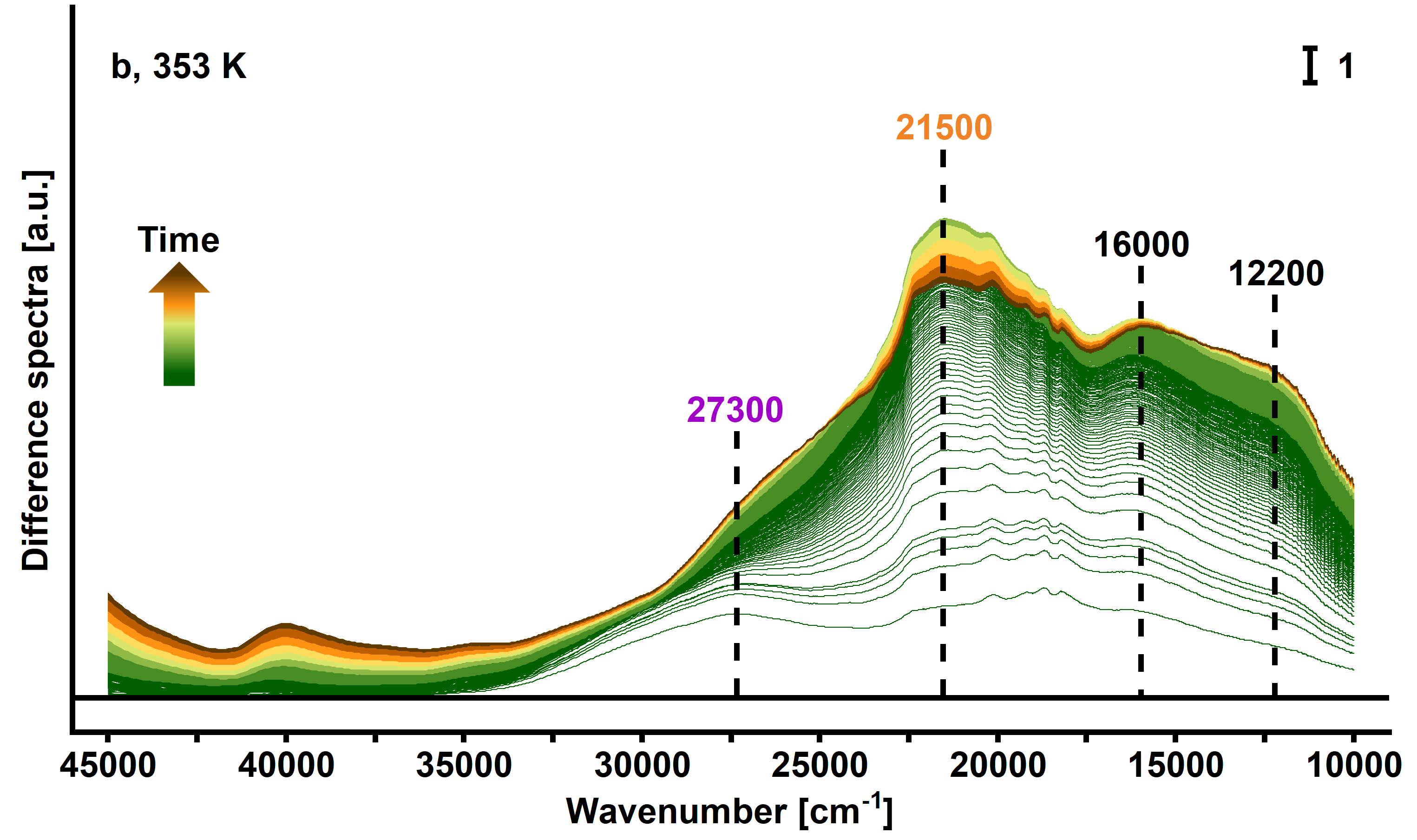

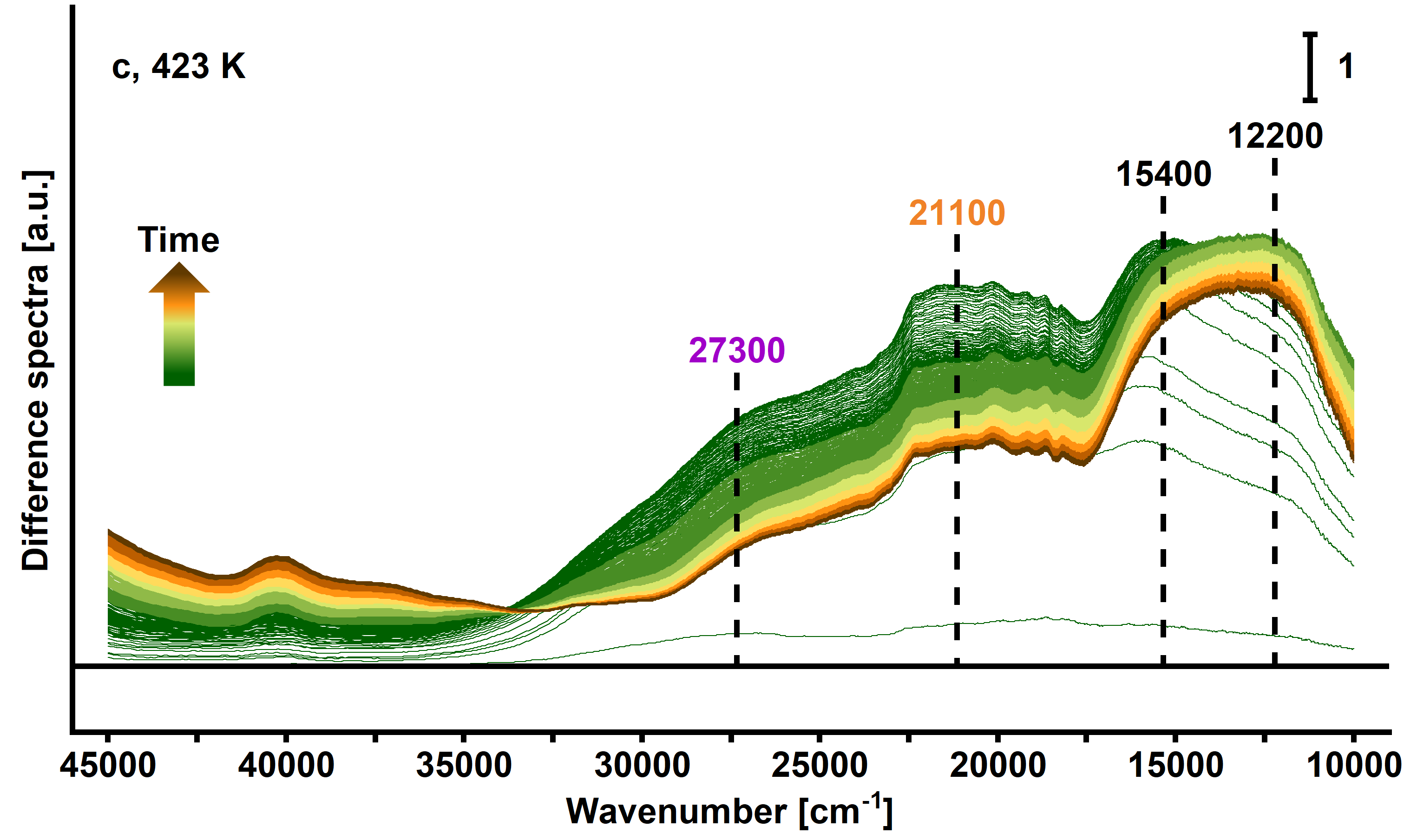

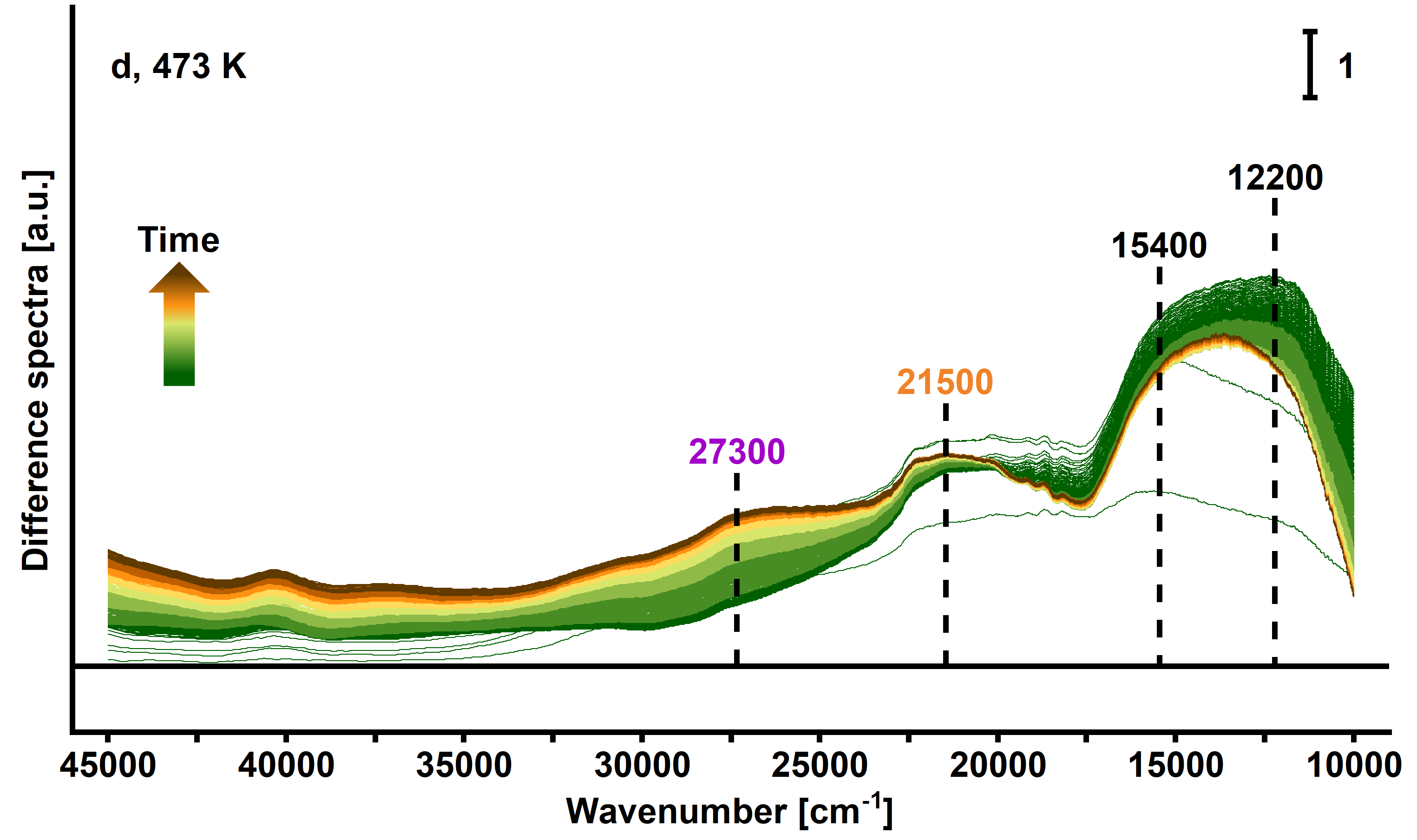

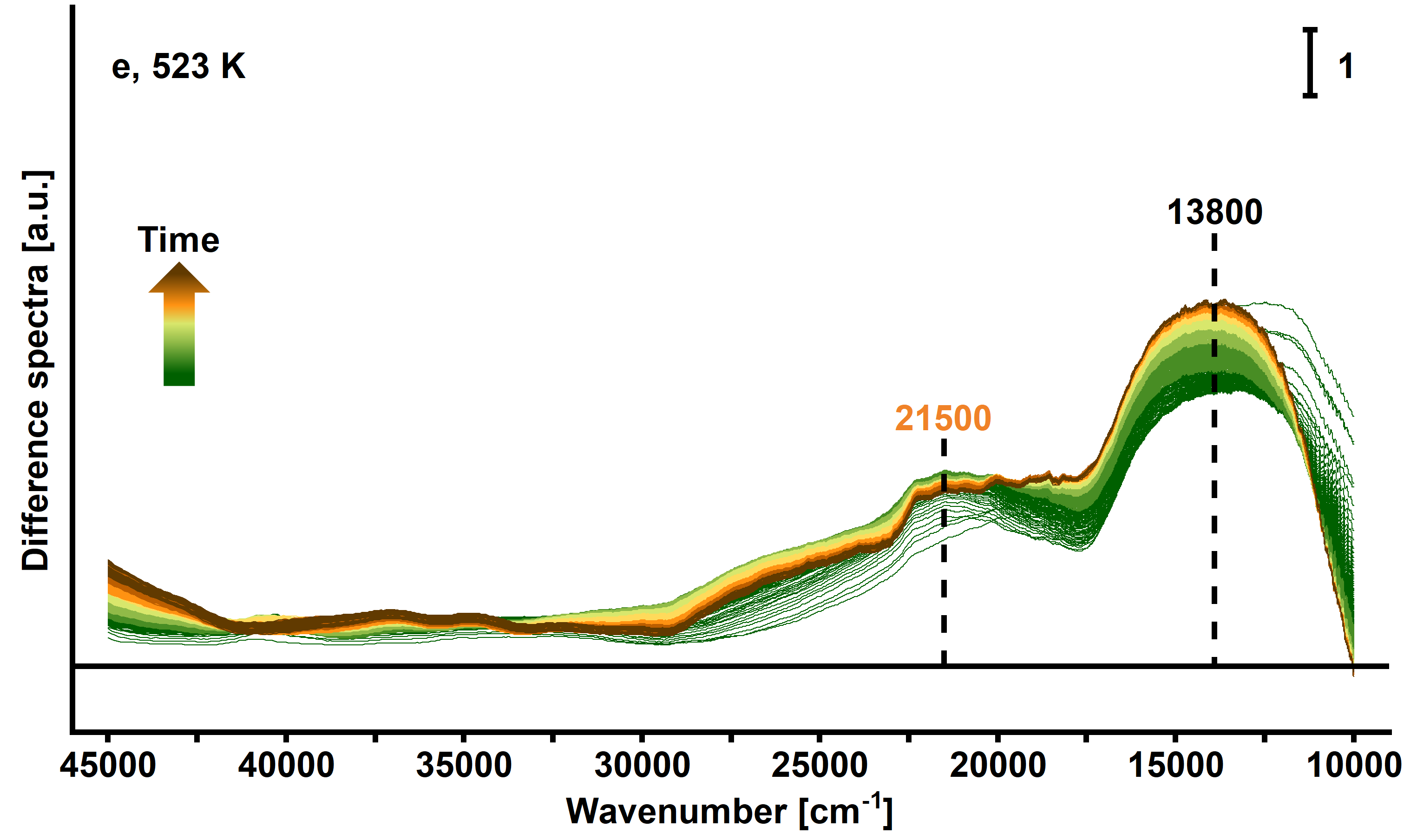

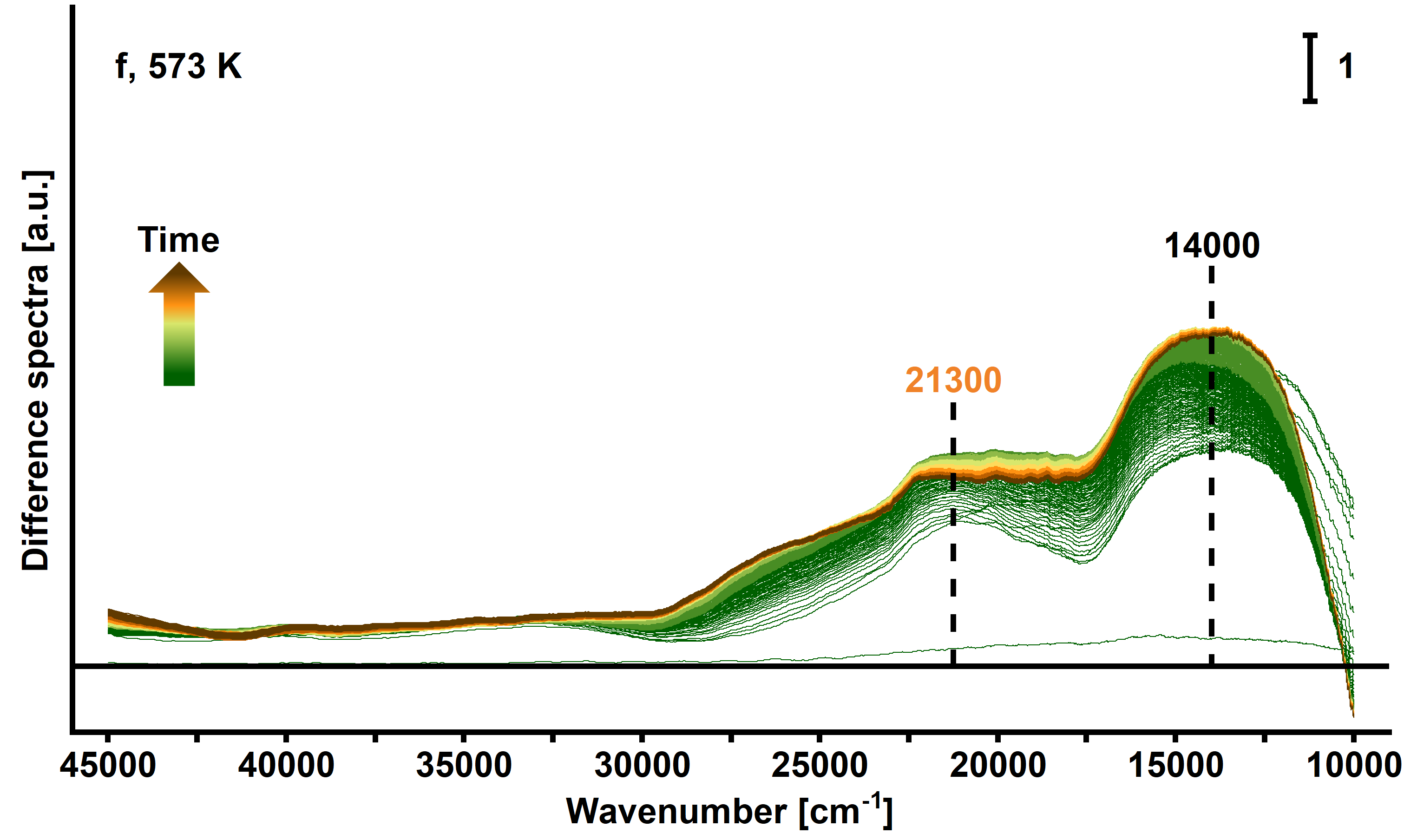

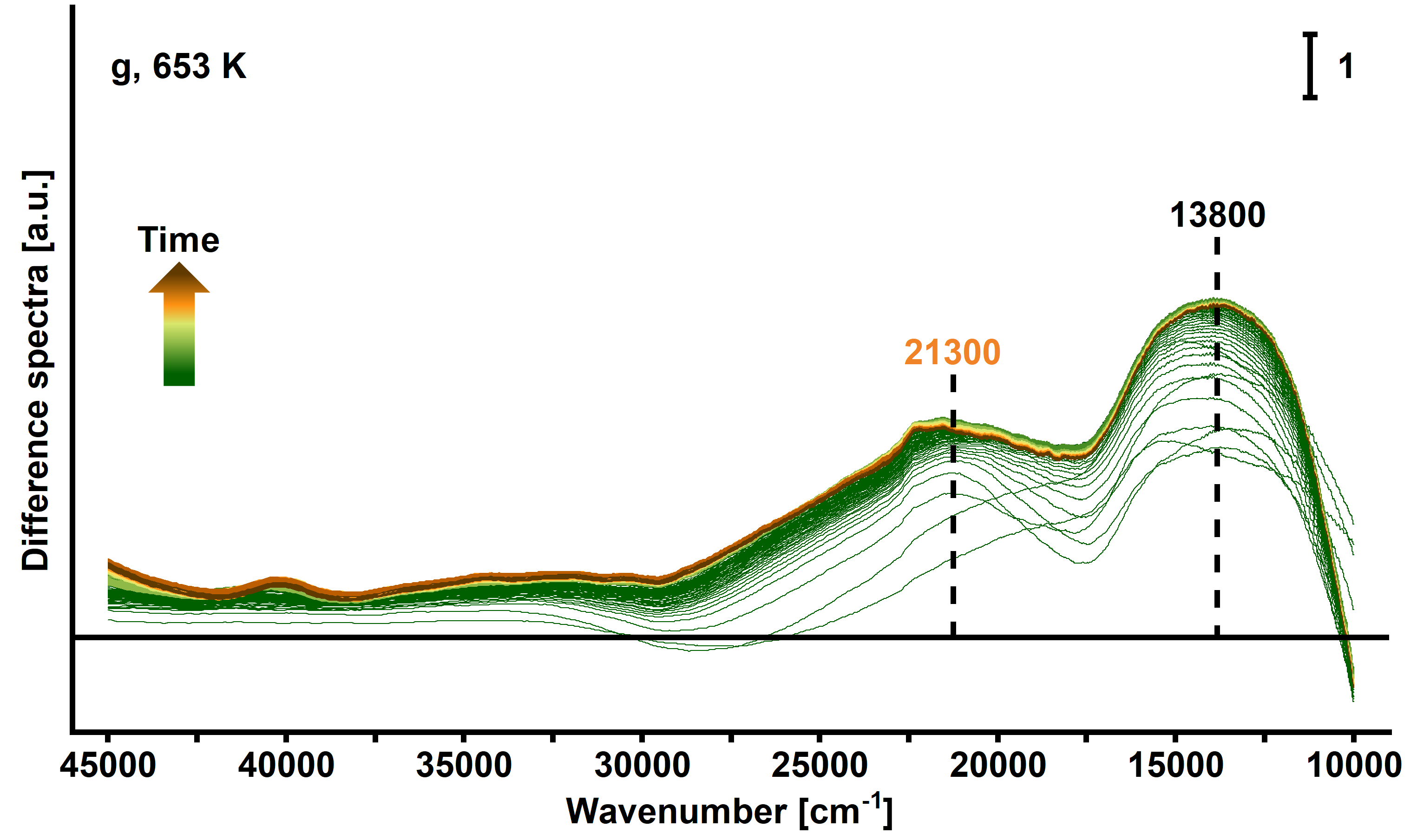

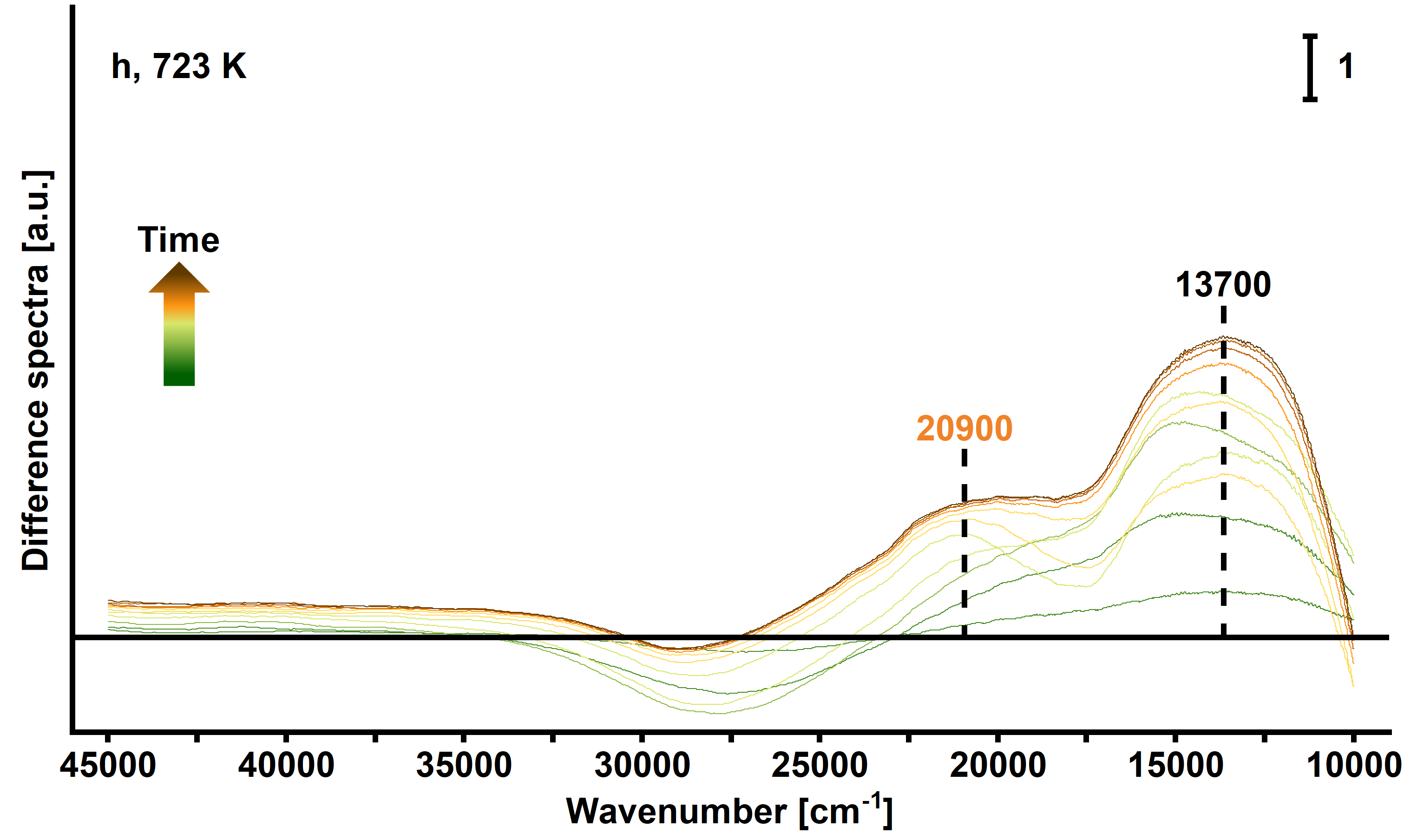


**Figure S24.** Operando UV-Vis difference spectra of Cu*_3.6_*MFI*_11.5_* during isothermal re-activation at 313 K for 21 h (a), 353 K for 22 h (b), 423 K for 16 h (c), 473 K for 17 h (d), 523 K for 17 h (e), 573 K for 17 h (f), 653 K for 17 h (g), and 723 K for 2 h (h) with O_2_. The characteristic bands of S4 and S5 are highlighted in purple and orange.


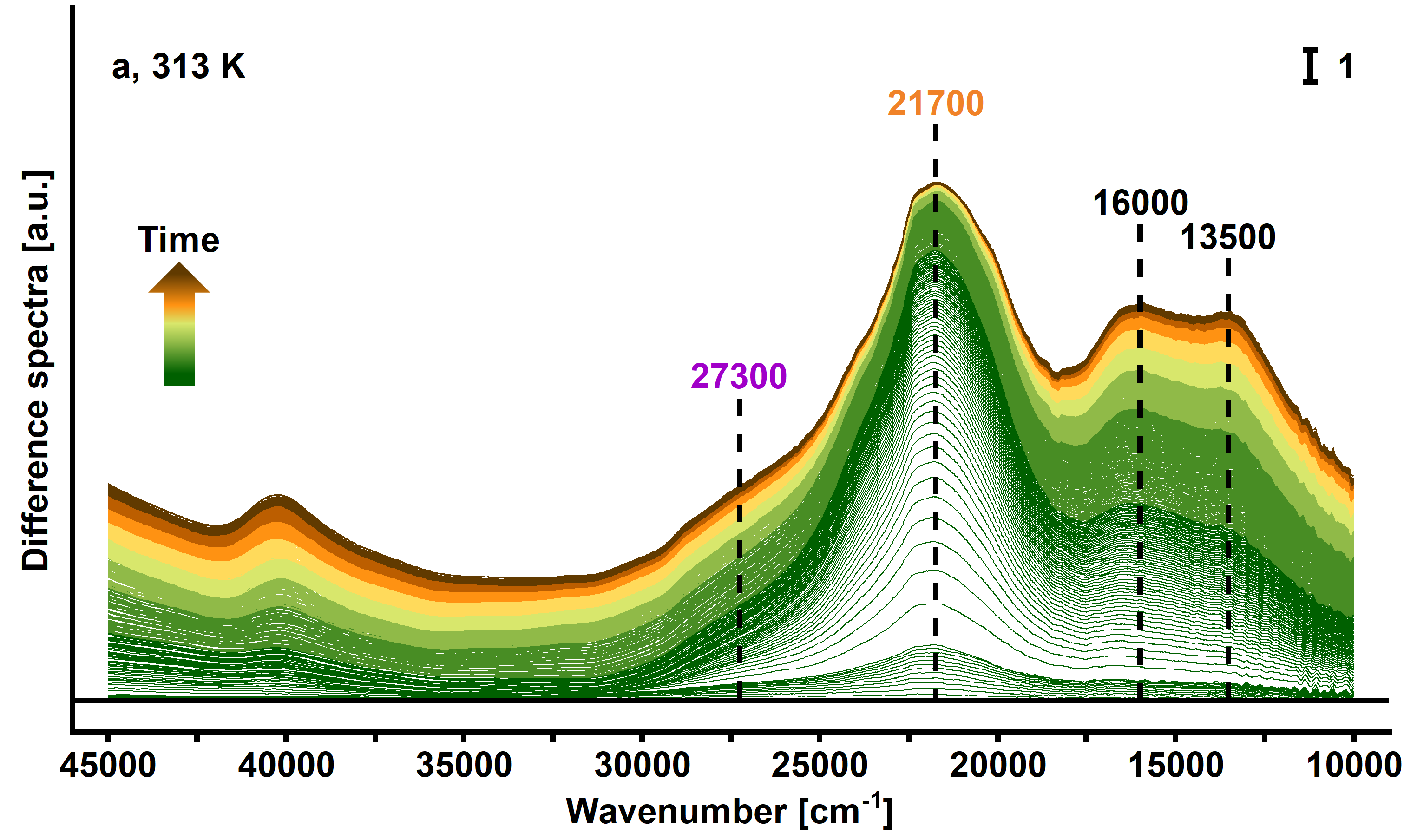

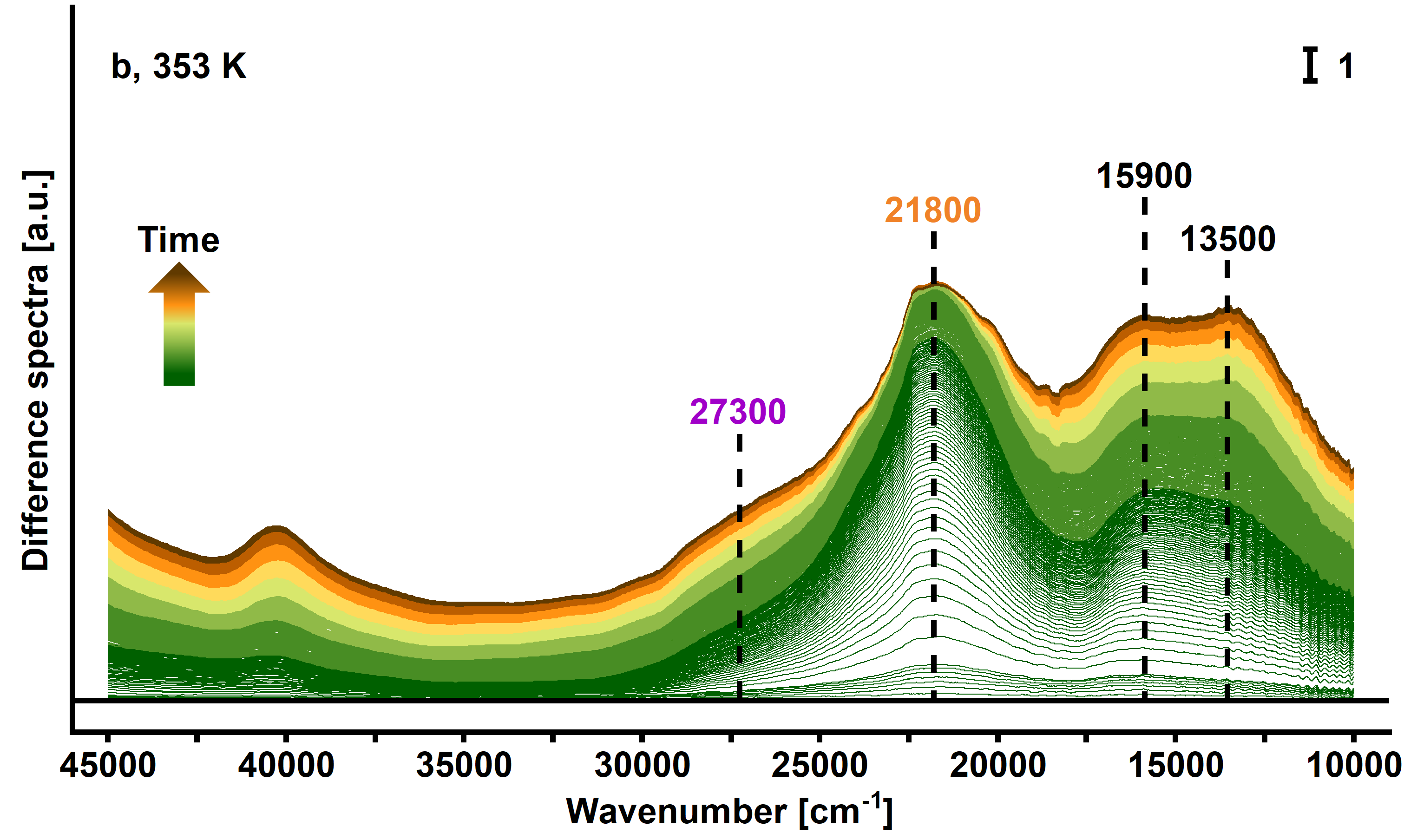

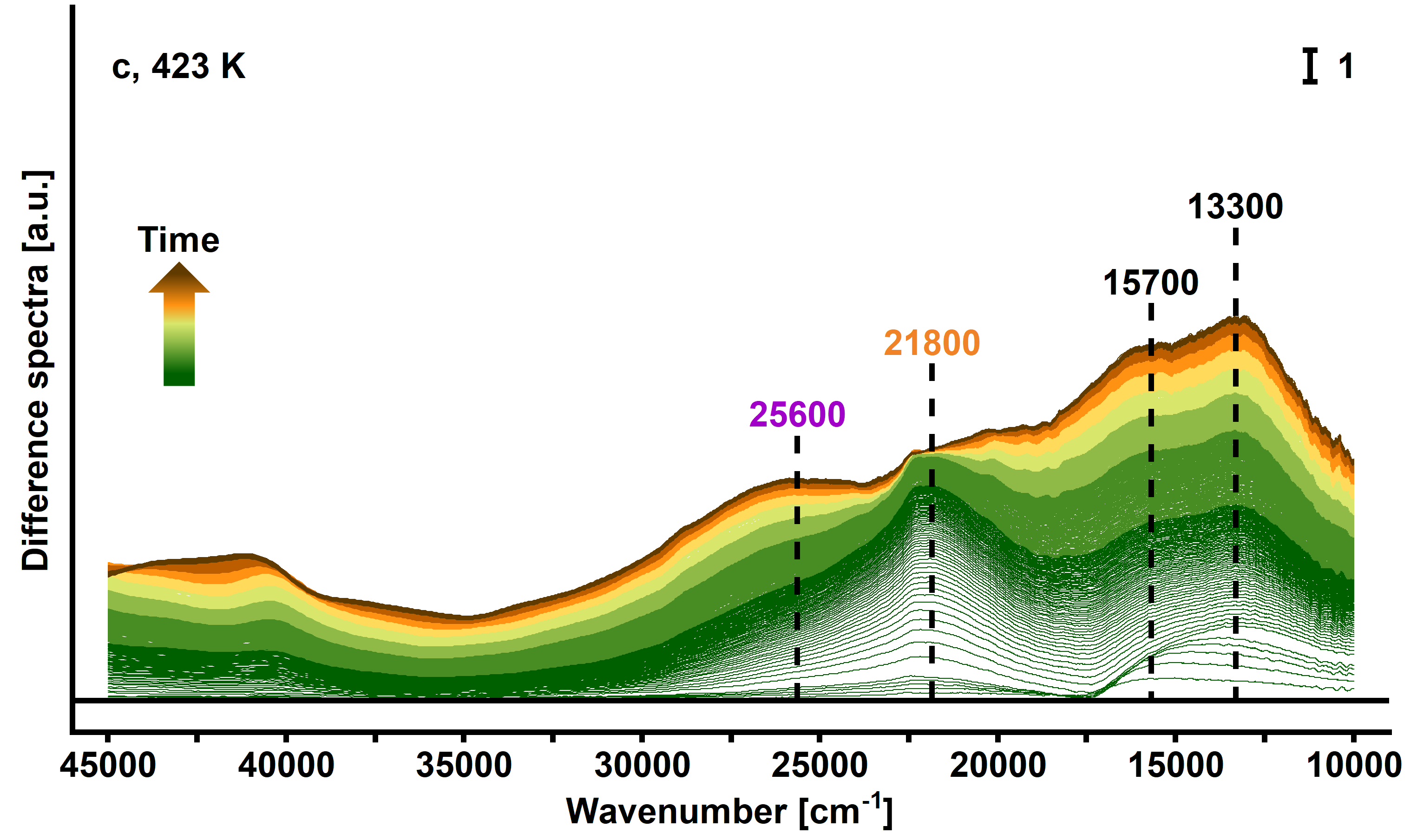

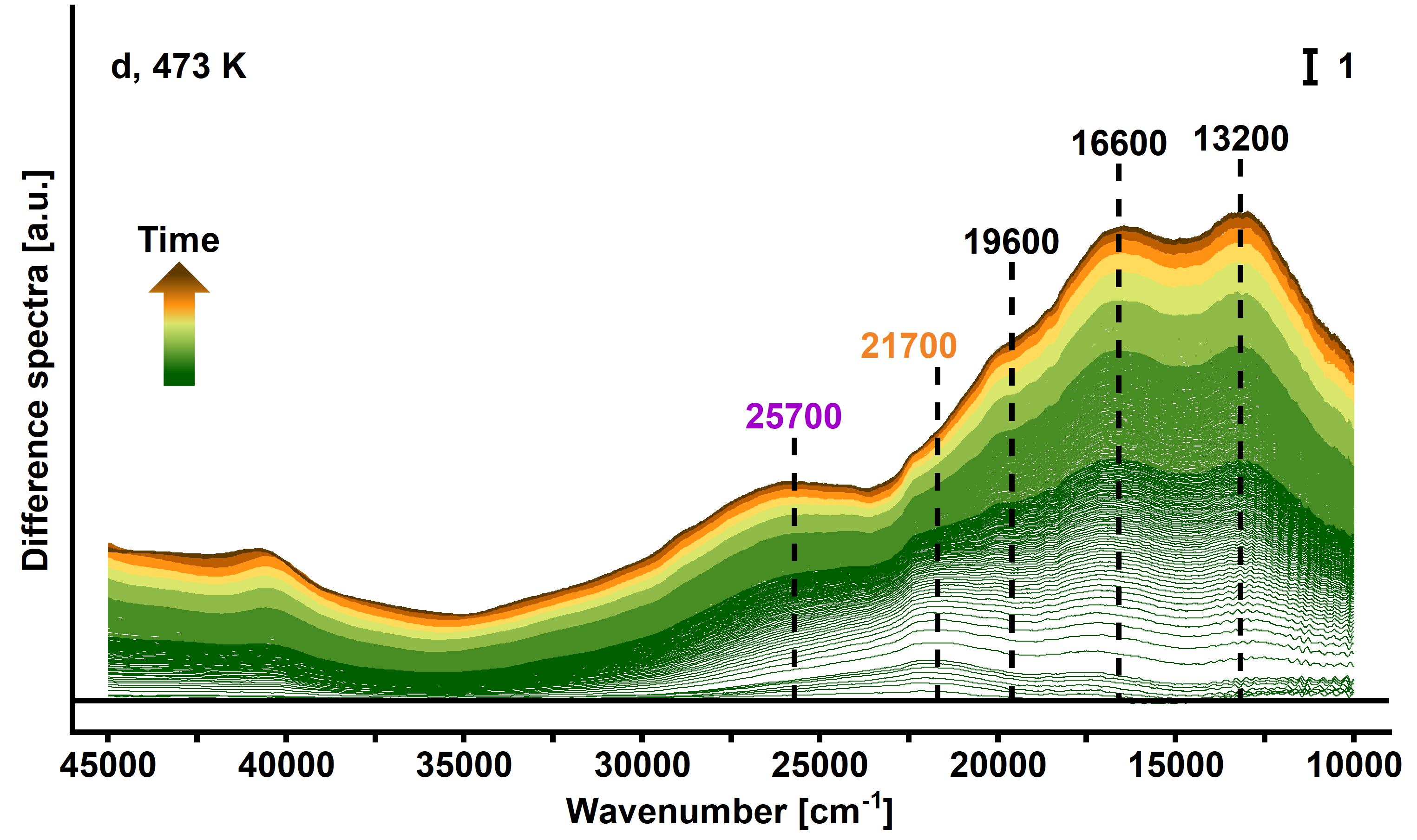

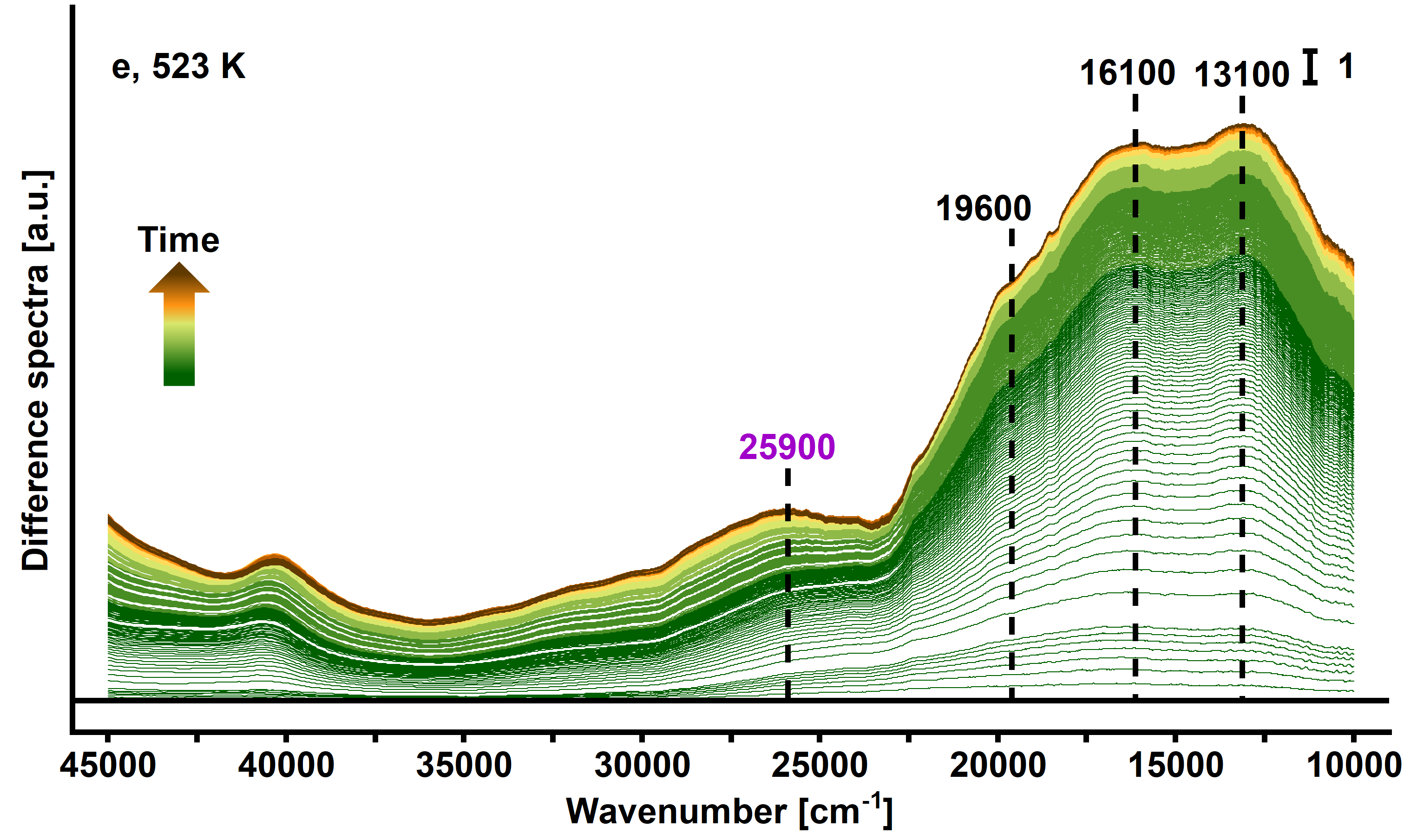

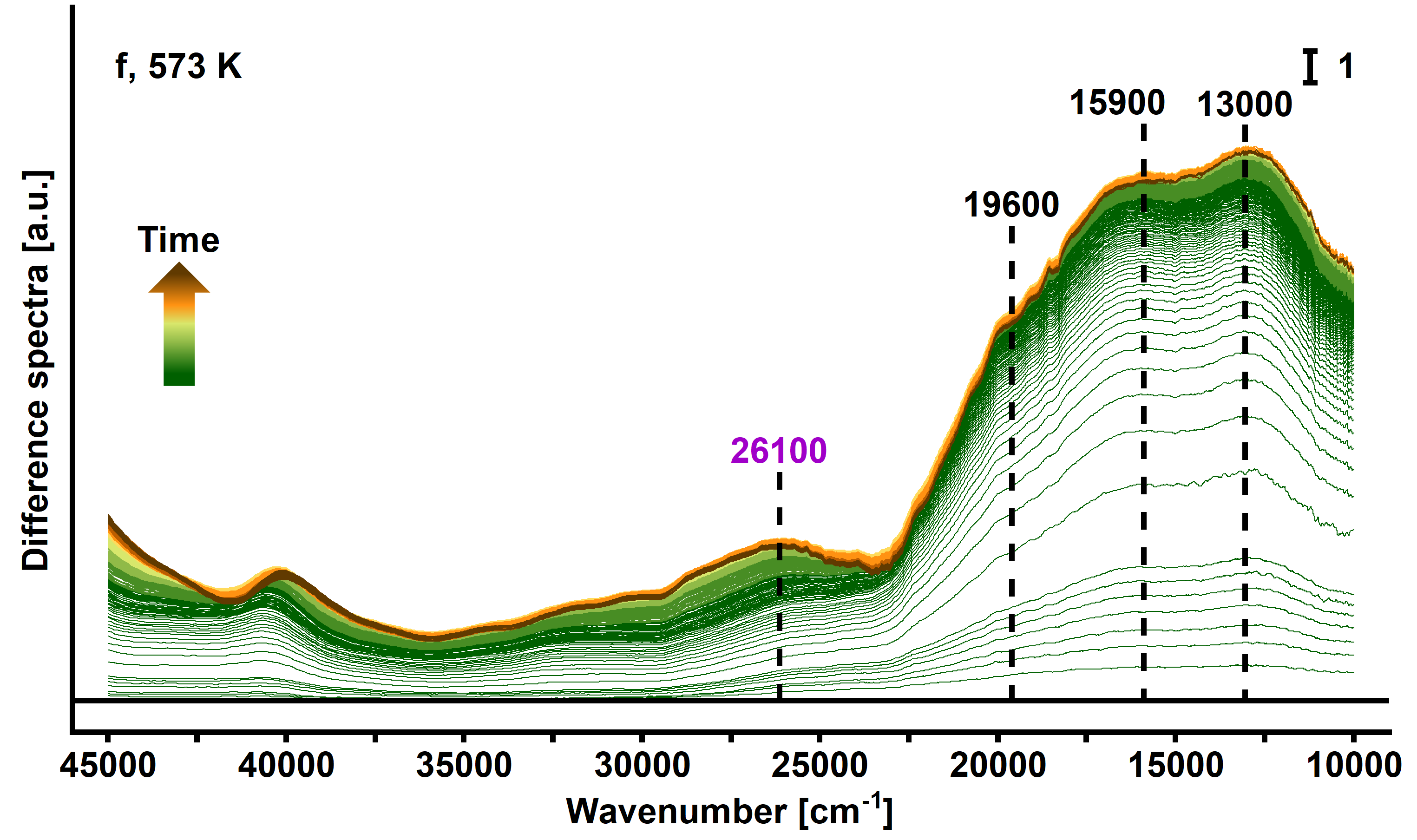

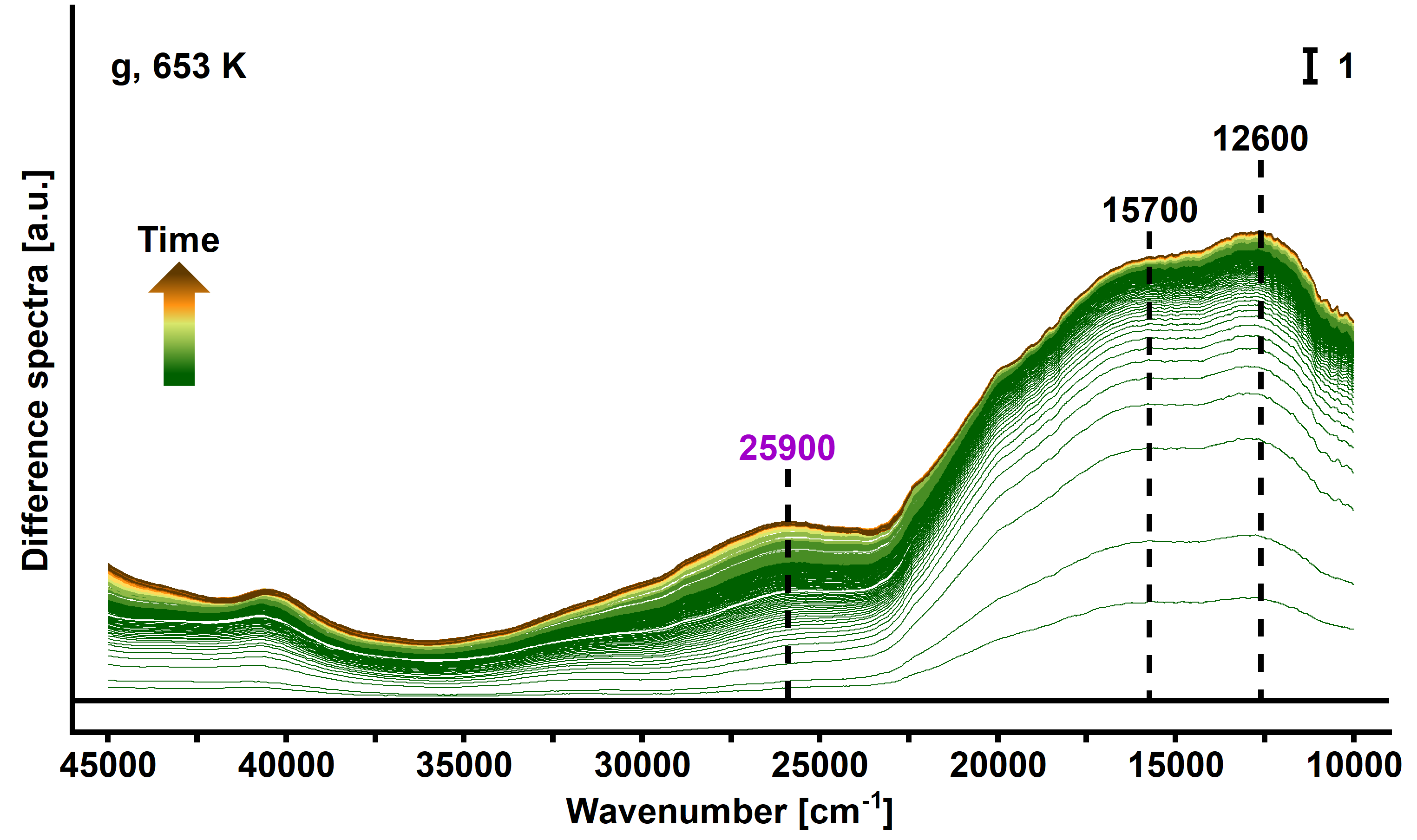

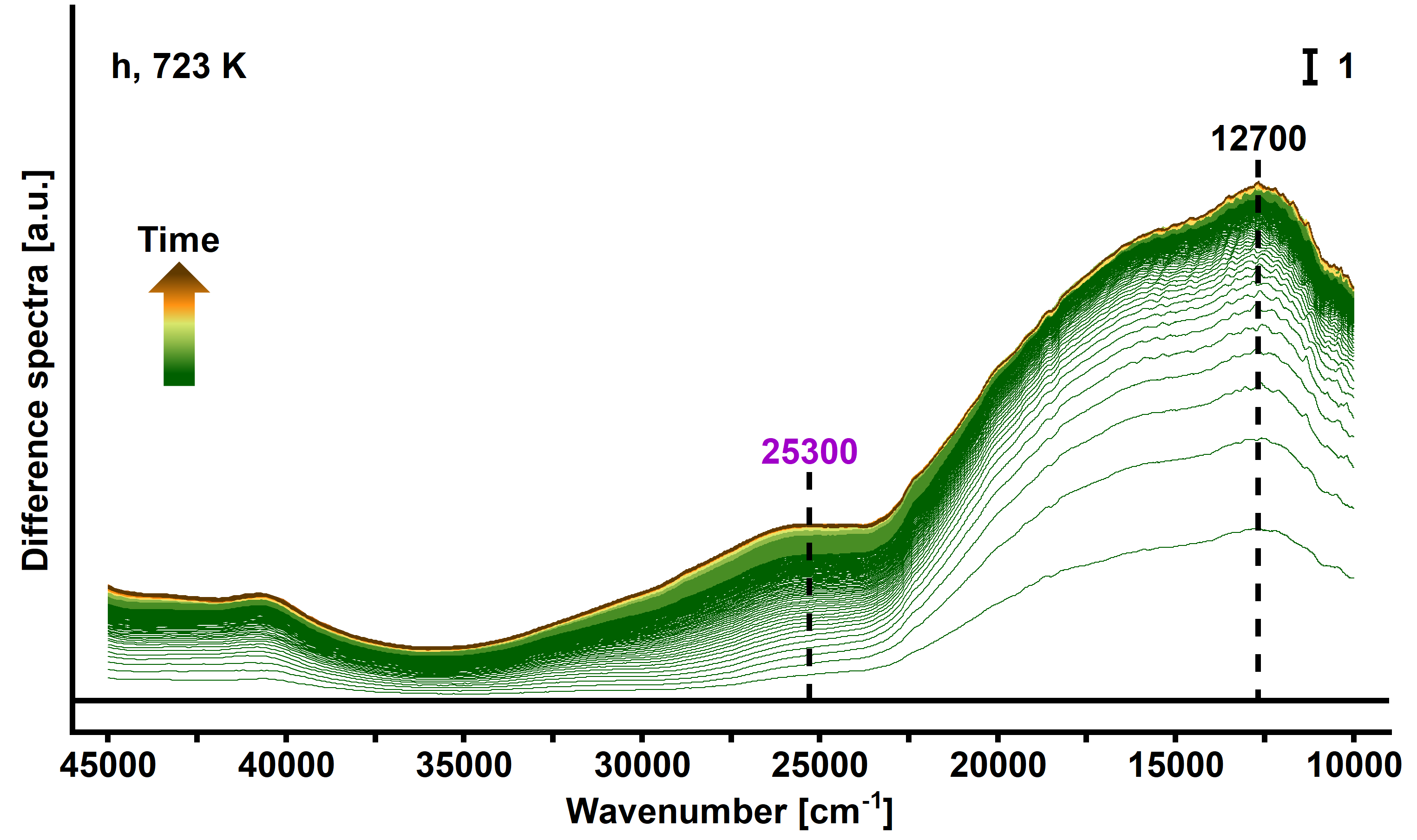


**Figure S25.** Operando UV-Vis difference spectra of Cu*_3.2_*CHA*_11.0_* during isothermal re-activation at 313 K for 24 h (a), 353 K for 24 h (b), 423 K for 18 h (c), 473 K for 24 h (d), 523 K for 23 h (e), 573 K for 24 h (f), 653 K for 13 h (g), and 723 K for 22 h (h) with O_2_. The characteristic bands of S4 and S5 are highlighted in purple and orange.


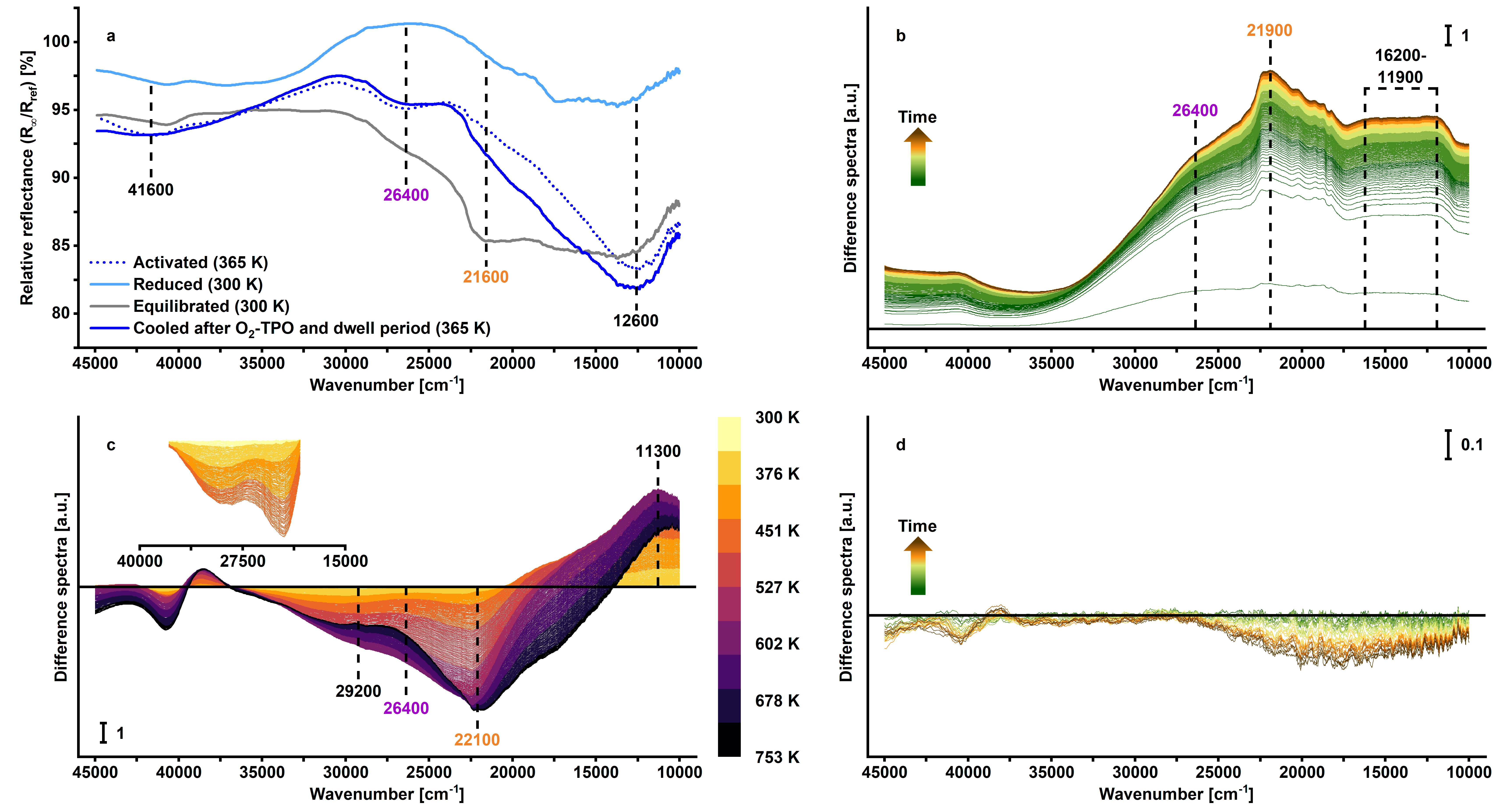


**Figure S26.** In situ UV-Vis relative reflectance spectra of Cu*_3.2_*MOR*_10.0_* after specific segments of the O_2_-TPO (a). Values in parenthesis correspond to the spectrum acquisition temperature. Spectra after activation, reduction, and cool down were recorded in vacuum. The spectrum after isothermal equilibration was recorded in O_2_. Difference spectra during isothermal equilibration at 300 K in O_2_ for 1 h (b), O_2_-TPO in the range from 300 to 753 K (c), and dwell at 753 K in O_2_ for 5 min (d). The inset in Fig. S25c shows an enlarged section of the spectra in the range from 300 to 470 K, potentially corresponding to the decomposition of the [Cu_2_(µ-η^2^:η^2^-O_2_)]^2+^ precursor. The characteristic bands of S4 and S5 are highlighted in purple and orange.


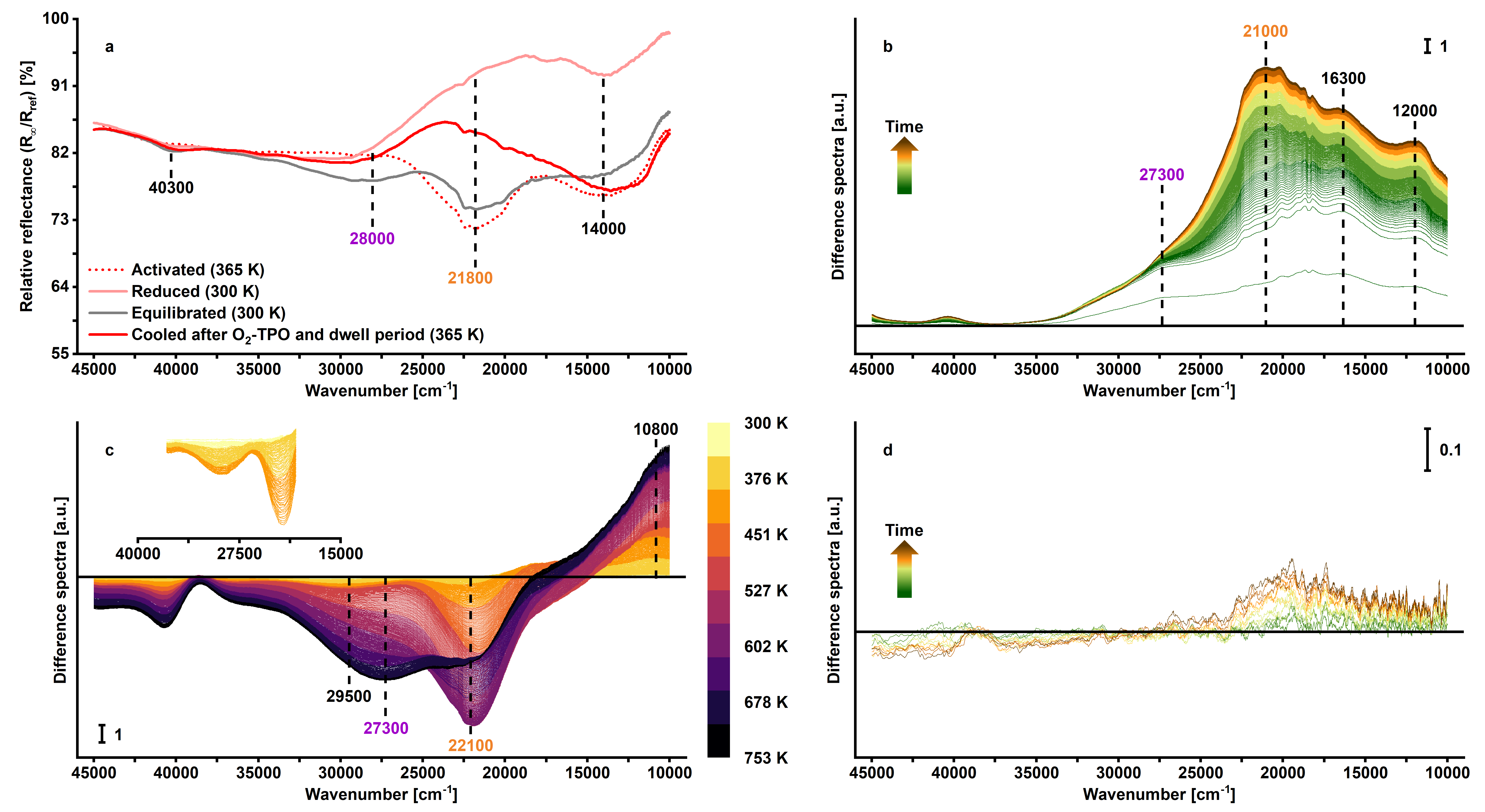


**Figure S27.** In situ UV-Vis relative reflectance spectra of Cu*_3.6_*MFI*_11.5_* after specific segments of the O_2_-TPO (a). Values in parenthesis correspond to the spectrum acquisition temperature. Spectra after activation, reduction, and cool down were recorded in vacuum. The spectrum after isothermal equilibration was recorded in O_2_. Difference spectra during isothermal equilibration at 300 K in O_2_ for 1 h (b), O_2_-TPO in the range from 300 to 753 K (c), and dwell at 753 K in O_2_ for 2 min (d). The inset in Fig. S26c shows an enlarged section of the spectra in the range from 300 to 413 K, potentially corresponding to the decomposition of the
[Cu_2_(µ-η^2^:η^2^-O_2_)]^2+^ precursor. The characteristic bands of S4 and S5 are highlighted in purple and orange.

**
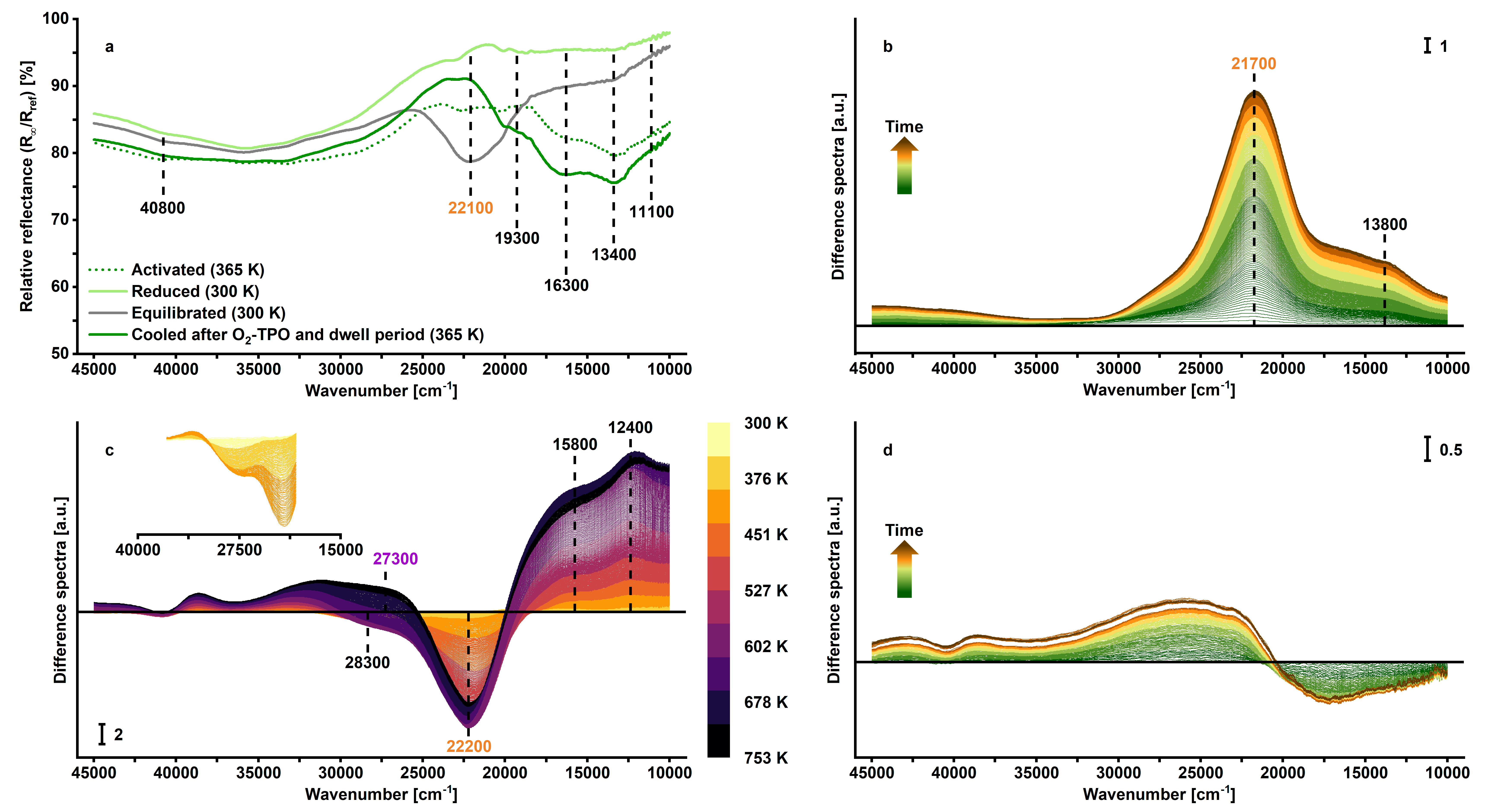
**

**Figure S28.** In situ UV-Vis relative reflectance spectra of Cu*_3.2_*CHA*_11.0_* after specific segments of the O_2_-TPO (a). Values in parenthesis correspond to the spectrum acquisition temperature. Spectra after activation, reduction, and cool down were recorded in vacuum. The spectrum after isothermal equilibration was recorded in O_2_. Difference spectra during isothermal equilibration at 300 K in O_2_ for 1 h (b), O_2_-TPO in the range from 300 to 753 K (c), and dwell at 753 K in O_2_ for 30 min (d). The inset in Fig. S27c shows an enlarged section of the spectra in the range from 300 to 426 K, potentially corresponding to the decomposition of the [Cu_2_(µ-η^2^:η^2^-O_2_)]^2+^ precursor. The characteristic bands of S4 and S5 are highlighted in purple and orange.

The spectra of the three different materials after the regular activation (Figs. S25a, S26a, and S27a) are characterized by the typical absorption bands associated with S1-S5. Reduction in CH_4_ induces an overall broad loss in the spectral intensity due to the conversion of Cu(II) to Cu(I). Admission of O_2_ onto the reduced samples at 300 K during the isothermal equilibration period causes an increase in the absorbance of features in the range from 27300 to 21000 cm^-1^, arising from the distinct signals of S4 and S5. This is also highlighted by the corresponding difference spectra recorded during this phase (Figs. S25b, S26b, and S27b). Compared to Cu*_3.2_*MOR*_10.0_* and Cu*_3.6_*MFI*_11.5_*, the increase in the intensity of the band arising from S4 in Cu*_3.2_*CHA*_11.0_* is less pronounced. This originates from the overall slower oxidation rate of this sample. As highlighted by Fig. S24a, a growth in the absorbance of the feature attributed to S4 can indeed be observed upon increasing the reaction duration to 38 h. As demonstrated by Figs. S25c, S26c, and S27c, the subsequent increase in temperature throughout the O_2_-TPO results in an initial loss in the intensity of signals emerging from S4 and S5 in each Cu-zeolite due to the progressive autoreduction of the Cu(II) dimers despite the presence of O_2_. This is accompanied by a decrease in the absorbance of a band in the range from 29500-28300 cm^-1^, which might stem from the decomposition of a [Cu_2_(µ-η^2^:η^2^-O_2_)]^2+^ precursor.^[20]^ At the same time, the intensity of features in the low-energy region of the spectra increases, which can be correlated to the formation of S1-S3 at elevated temperatures. Notably, the intensity of the signals arising from S4 and S5 re-increases again upon surpassing ~580 K. However, this rise in absorbance can only be identified for the bands originating from the framework-dependent, high-temperature stable, dimeric Cu(II) species. Consequently, the spectra of Cu*_3.2_*MOR*_10.0_* display an exclusive growth in the intensity of the feature arising from S4, whereas the spectra of Cu*_3.6_*MFI*_11.5_* show the opposite behavior. In Cu*_3.2_*CHA*_11.0_*, the signals corresponding to both S4 and S5 re-increase since both of these Cu(II) dimers are stabilized in this material. This alteration of the absorbance arises from the desorption of H_2_O from S4 and S5, which is generated by oxidation of hydrocarbon residues during the O_2_-TPO. The latter originate from the initial reduction of the samples in CH_4_. Throughout the final dwell period at 753 K in O_2_ (Figs. S25d, 26d, and 26c), no major spectral changes can be detected. After the subsequent cool down to 365 K in O_2_, the intensity of bands emerging from framework-dependent stable dimeric Cu(II) species is consistently lower than after the initial regular activation (Figs. S25a, S26a, and S27a). This is particularly pronounced in Cu*_3.6_*MFI*_11.5_* and Cu*_3.2_*CHA*_11.0_*. Moreover, a broad increase in the absorbance of the low-frequency features in Cu*_3.2_*MOR*_10.0_* and Cu*_3.2_*CHA*_11.0_* can be observed. These spectral modifications originate from the re-adsorption of the generated H_2_O on S4 and S5.^[12,21,22]^

**
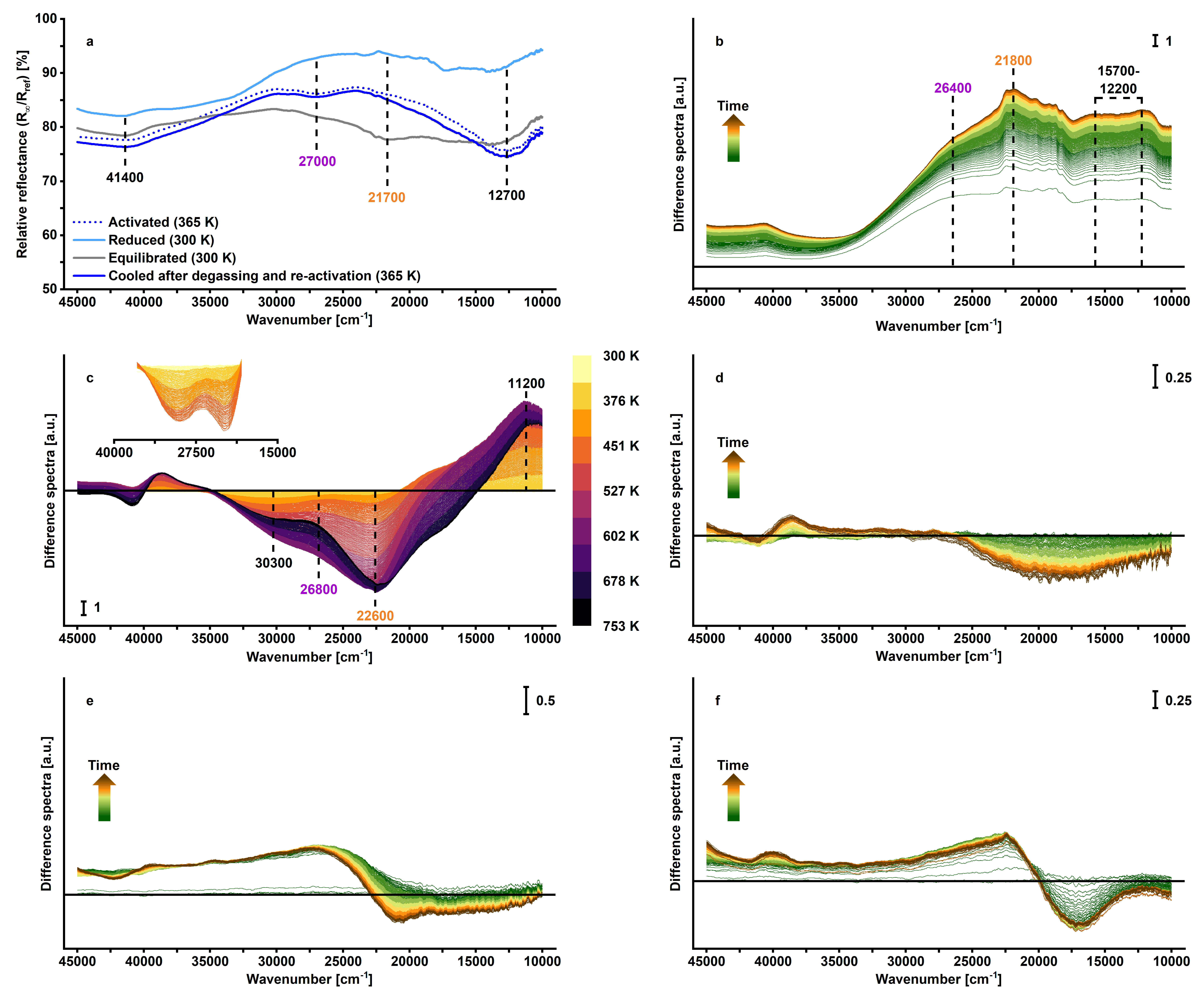
**

**Figure S29.** In situ UV-Vis relative reflectance spectra of Cu*_3.2_*MOR*_10.0_* after specific segments of the modified O_2_-TPO (a). Values in parenthesis correspond to the spectrum acquisition temperature. Spectra after activation, reduction, and cool down were recorded in vacuum. The spectrum after isothermal equilibration was recorded in O_2_. Difference spectra during isothermal equilibration at 300 K in O_2_ for 1 h (b), O_2_-TPO in the range from 300 to 753 K (c), dwell at 753 K in O_2_ for 30 min (d), evacuation at 753 K for 1 h (e), and re-activation at 753 K in O_2_ for 1 h (f). The inset in Fig. S28c shows an enlarged section of the spectra in the range from 300 to 449 K, potentially corresponding to the decomposition of the [Cu_2_(µ-η^2^:η^2^-O_2_)]^2+^ precursor. The characteristic bands of S4 and S5 are highlighted in purple and orange.

**
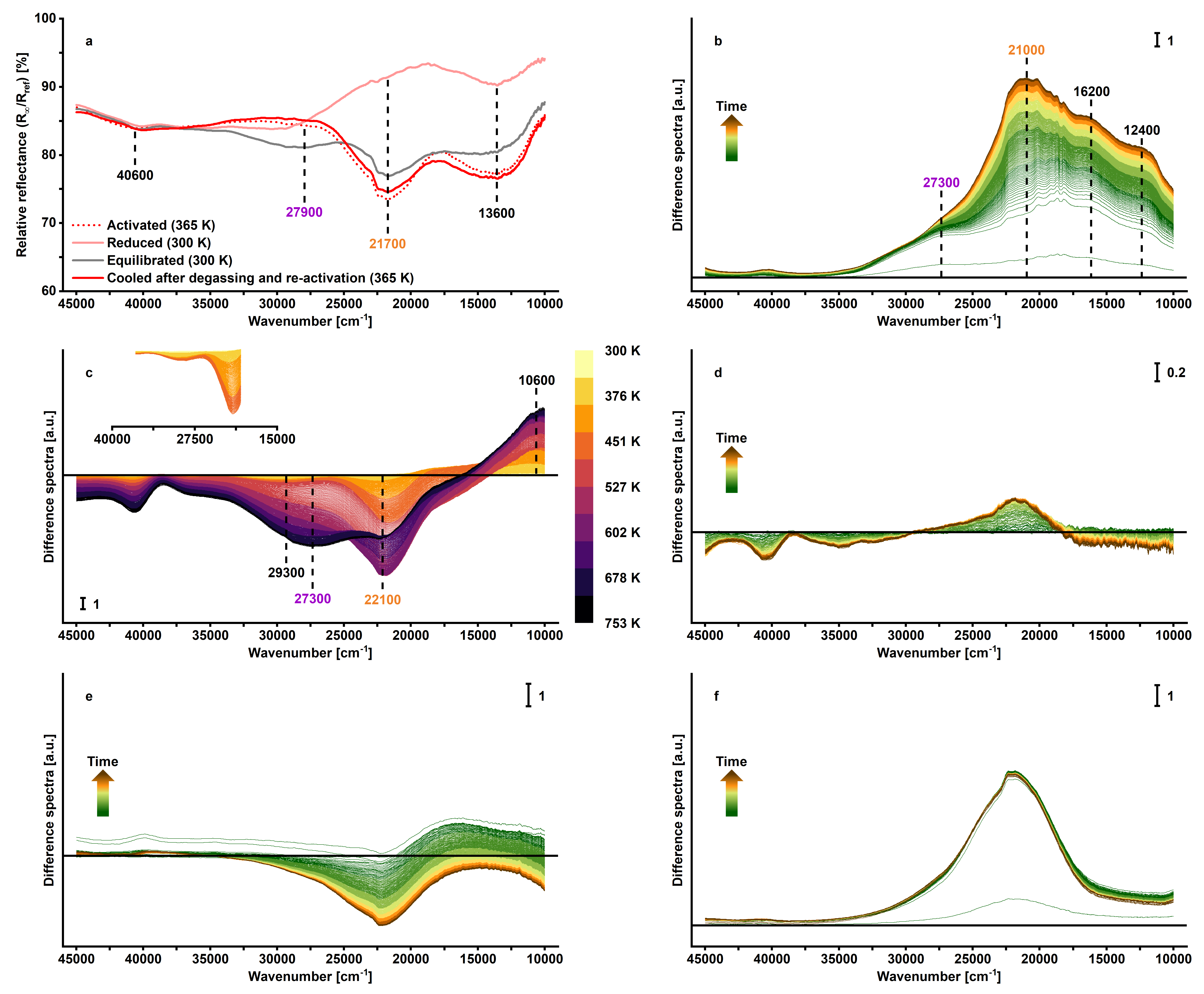
**

**Figure S30.** In situ UV-Vis relative reflectance spectra of Cu*_3.6_*MFI*_11.5_* after specific segments of the modified O_2_-TPO (a). Values in parenthesis correspond to the spectrum acquisition temperature. Spectra after activation, reduction, and cool down were recorded in vacuum. The spectrum after isothermal equilibration was recorded in O_2_. Difference spectra during isothermal equilibration at 300 K in O_2_ for 1 h (b), O_2_-TPO in the range from 300 to 753 K (c), dwell at 753 K in O_2_ for 30 min (d), evacuation at 753 K for 1 h (e), and re-activation at 753 K in O_2_ for 1 h (f). The inset in Fig. S29c shows an enlarged section of the spectra in the range from 300 to 475 K, potentially corresponding to the decomposition of the [Cu_2_(µ-η^2^:η^2^-O_2_)]^2+^ precursor. The characteristic bands of S4 and S5 are highlighted in purple and orange.


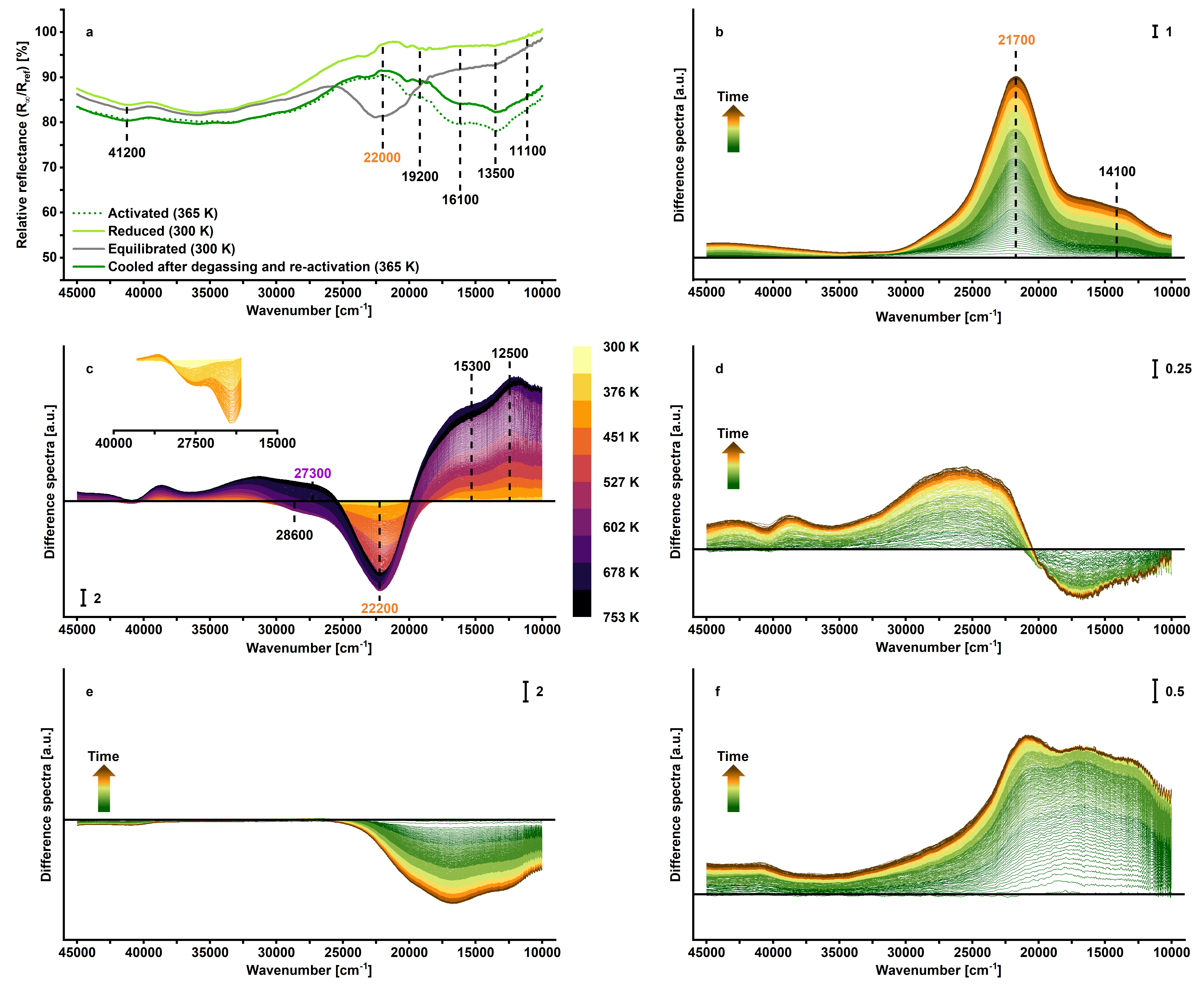


**Figure S31.** In situ UV-Vis relative reflectance spectra of Cu*_3.2_*CHA*_11.0_* after specific segments of the modified O_2_-TPO (a). Values in parenthesis correspond to the spectrum acquisition temperature. Spectra after activation, reduction, and cool down were recorded in vacuum. The spectrum after isothermal equilibration was recorded in O_2_. Difference spectra during isothermal equilibration at 300 K in O_2_ for 1 h (b), O_2_-TPO in the range from 300 to 753 K (c), dwell at 753 K in O_2_ for 30 min (d), evacuation at 753 K for 1 h (e), and re-activation at 753 K in O_2_ for 1 h (f). The inset in Fig. S30c shows an enlarged section of the spectra in the range from 300 to 426 K, potentially corresponding to the decomposition of the [Cu_2_(µ-η^2^:η^2^-O_2_)]^2+^ precursor. The characteristic bands of S4 and S5 are highlighted in purple and orange.

The spectra acquired throughout the isothermal equilibration, temperature ramp, and dwell period at 753 K of the modified O_2_-TPO procedure (Figs S28b-d, S29b-d, and 30b-d) behave analogous to the ones of the regular O_2_-TPO (Figs S25b-d, S26b-d, and 27b-d). The spectral evolution during the subsequent evacuation and re-activation period at 753 K (Figs. S28e and f, S29 e and f, and S30e and f) can be best described by an autoreduction and re-oxidation process of the different materials. Importantly, no significant decline in the intensity of bands corresponding to the framework-dependent high-temperature stable Cu(II) dimers can be observed after the cool down to 365 K in O_2_ following the modified O_2_-TPO, indicating that the generated H_2_O was effectively removed by the dehydration step at 753 K.


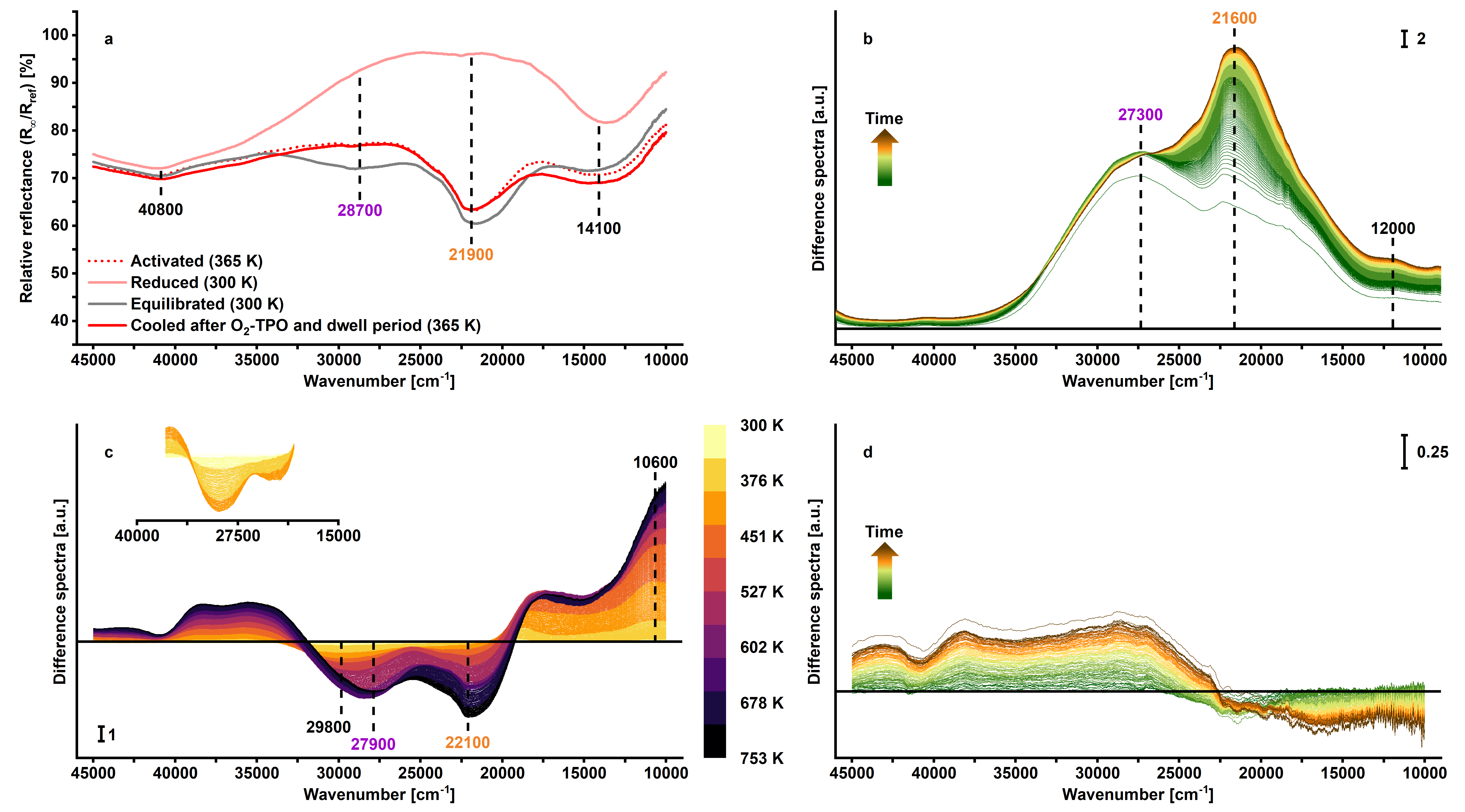


**Figure S32.** In situ UV-Vis relative reflectance spectra of Cu*_3.6_*MFI*_11.5_* after specific segments of the O_2_-TPO after material reduction in CO (a). Values in parenthesis correspond to the spectrum acquisition temperature. Spectra after activation, reduction, and cool down were recorded in vacuum. The spectrum after isothermal equilibration was recorded in O_2_. Difference spectra during isothermal equilibration at 300 K in O_2_ for 1 h (b), O_2_-TPO in the range from 300 to 753 K (c), and dwell at 753 K in O_2_ for 20 min (d). The inset in Fig. S31c shows an enlarged section of the spectra in the range from 300 to 423 K, potentially corresponding to the decomposition of the [Cu_2_(µ-η^2^:η^2^-O_2_)]^2+^ precursor. The characteristic bands of S4 and S5 are highlighted in purple and orange.

In order to provide further evidence for the formation of H_2_O via the oxidation of hydrocarbon species generated during the initial reduction of the Cu-zeolites with CH_4_, Cu*_3.6_*MFI*_11.5_* was exemplarily subjected to a regular O_2_-TPO after treatment in CO instead of CH_4_ since the reduction in CO should not yield hydrocarbon residues. The spectra behave similar to the ones recorded throughout the regular O_2_-TPO of Cu*_3.6_*MFI*_11.5_* following material reduction with CH_4_ (Fig. S26) with the crucial difference that the intensity of the signal arising from S5 is not characterized by a re-increase at > 580 K during the temperature ramp (Fig. S31c). Similarly, the absorbance of this band does not vanish throughout the subsequent cool down in O2 (Fig. S31a), demonstrating that no hydrocarbon deposits, which could have resulted in the generation of H_2_O, have been formed.





**Figure S33.** Normalized O_2_ pressure throughout the isothermal re-activation of Cu*_3.6_*MFI*_11.5_* (a) and Cu*_3.2_*CHA*_11.0_* (b) at temperatures in the range from 313 to
723 K.

**2.6 In Situ FTIR Spectroscopy**





**Figure S34.** Selected sections of the in situ FTIR spectra of adsorbed surface species after reaction with ~365 mbar CH_4_ for 1 h at 753 K and subsequent evacuation at 753 K for 2 h. The general presence of absorption bands indicates the existence of adsorbed hydrocarbon residues on the materials. However, a proper assignment of the observed signals is ambiguous due to their low overall intensity.

# 3. References

[1] J. W. A. Fischer, F. Buttignol, A. Brenig, D. Klose, D. Ferri, V. Sushkevich, J. A. van Bokhoven, G. Jeschke, *Catal. Today* **2023**, *429*, 114503–114512.

[2] G. Zichittella, Y. Polyhach, R. Tschaggelar, G. Jeschke, J. Pérez-Ramírez, *Angew. Chem. Int. Ed.* **2021**, *60*, 3596–3602.

[3] R. Czoch, *Appl. Magn. Reson.* **1996**, *10*, 293–317.

[4] A. Godiksen, O. L. Isaksen, S. B. Rasmussen, P. N. R. Vennestrøm, S. Mossin, *ChemCatChem* **2018**, *10*, 366–370.

[5] C. Negri, M. Signorile, N. G. Porcaro, E. Borfecchia, G. Berlier, T. V. W. Janssens, S. Bordiga, *Appl. Catal. A: Gen.* **2019**, *578*, 1–9.

[6] H. Zhang, J. Lv, Z. Zhang, C. Du, S. Wang, J. Lin, S. Wan, Y. Wang, H. Xiong, *ChemCatChem* **2022**, *14*, e202101609.

[7] V. L. Sushkevich, A. V. Smirnov, J. A. van Bokhoven, *J. Phys. Chem. C* **2019**, *123*, 9926–9934.

[8] M. A. Artsiusheuski, J. A. van Bokhoven, V. L. Sushkevich, *ACS Catal.* **2022**, *12*, 15626–15637.

[9] S. Bordiga, E. Groppo, G. Agostini, J. A. van Bokhoven, C. Lamberti, *Chem. Rev.* **2013**, *113*, 1736–1850.

[10] M. A. Artsiusheuski, O. Safonova, D. Palagin, J. A. van Bokhoven, V. L. Sushkevich, *J. Phys. Chem. C* **2023**, *127*, 9603–9615.

[11] V. W. Hu, S. I. Chan, G. S. Brown, *PNAS* **1977**, *74*, 3821–3825.

[12] A. Brenig, J. W. A. Fischer, D. Klose, G. Jeschke, J. A. van Bokhoven, V. L. Sushkevich, *Angew. Chem. Int. Ed.* **2024**, *63*, e202411662.

[13] A. Martini, E. Borfecchia, K. A. Lomachenko, I. A. Pankin, C. Negri, G. Berlier, P. Beato, H. Falsig, S. Bordiga, C. Lamberti, *Chem. Sci.* **2017**, *8*, 6836–6851.

[14] M. Mazur, *Analytica Chimica Acta* **2006**, *561*, 1–15.

[15] D. B. Lukyanov, T. Vazhnova, N. Cherkasov, J. L. Casci, J. J. Birtill, *J. Phys. Chem. C* **2014**, *118*, 23918–23929.

[16] J. Kliava, R. Berger, in *Smart Materials for Ranging Systems* (Eds.: J. Franse, V. Eremenko, V. Sirenko), Springer Netherlands, Dordrecht, **2006**, pp. 27–48.

[17] C. Carbone, F. Di Benedetto, P. Marescotti, C. Sangregorio, L. Sorace, N. Lima, M. Romanelli, G. Lucchetti, C. Cipriani, *Mineralogy and Petrology* **2005**, *85*, 19–32.

[18] R. Berger, J. Kliava, J.-C. Bissey, V. Baïetto, *J. Appl. Phys* **2000**, *87*, 7389–7396.

[19] Yu. A. Koksharov, D. A. Pankratov, S. P. Gubin, I. D. Kosobudsky, M. Beltran, Y. Khodorkovsky, A. M. Tishin, *J. Appl. Phys* **2001**, *89*, 2293–2298.

[20] P. J. Smeets, R. G. Hadt, J. S. Woertink, P. Vanelderen, R. A. Schoonheydt, B. F. Sels, E. I. Solomon, *J. Am. Chem. Soc.* **2010**, *132*, 14736–14738.

[21] J. W. A. Fischer, A. Brenig, D. Klose, J. A. van Bokhoven, V. L. Sushkevich, G. Jeschke, *Angew. Chem. Int. Ed.* **2023**, *135*, e202303574.

[22] V. L. Sushkevich, M. Artsiusheuski, D. Klose, G. Jeschke, J. A. van Bokhoven, *Angew. Chem. Int. Ed.* **2021**, *60*, 15944–15953.

**4. Author Contributions**

**Andreas Brenig:** data curation, formal analysis, investigation, validation, writing of original draft, equal; **Jörg W. A. Fischer:** data curation, formal analysis, investigation, validation, writing of original draft, equal; **Daniel Klose:** project administration; **Gunnar Jeschke:** funding acquisition, project administration, lead; **Jeroen A. van Bokhoven:** funding acquisition, project administration, lead; **Vitaly L. Sushkevich:** funding acquisition, project administration, lead.
